# Identification of amygdala-expressed genes associated with autism spectrum disorder

**DOI:** 10.1186/s13229-020-00346-1

**Published:** 2020-05-27

**Authors:** Maria Jesus Herrero, Dmitry Velmeshev, David Hernandez-Pineda, Saarthak Sethi, Shawn Sorrells, Payal Banerjee, Catherine Sullivan, Abha R. Gupta, Arnold R. Kriegstein, Joshua G. Corbin

**Affiliations:** 1 Center for Neuroscience Research, Children’s Research Institute, Children’s National Hospital, Washington, DC USA; 2 Eli and Edythe Broad Center of Regeneration Medicine and Stem Cell Research, University of California-San Francisco, San Francisco, CA USA; 3 Department of Neurology, University of California-San Francisco, San Francisco, CA USA; 4 Present Address: Department of Neuroscience, University of Pittsburgh, Pittsburgh, PA USA; 5 Center for Genetic Medicine, Children’s Research Institute, Children’s National Hospital, Washington, DC USA; 6 Department of Pediatrics and Child Study Center, Yale School of Medicine, New Haven, Connecticut USA

**Keywords:** Amygdala, Single nucleus RNA sequencing, Brain development, Autism spectrum disorder, ASD genes

## Abstract

**Background:**

Studies of individuals with autism spectrum disorder (ASD) have revealed a strong multigenic basis with the identification of hundreds of ASD susceptibility genes. ASD is characterized by social deficits and a range of other phenotypes, implicating complex genetics and involvement of a variety of brain regions. However, how mutations and mis-expression of select gene sets are associated with the behavioral components of ASD remains unknown. We reasoned that for genes to be associated with ASD core behaviors they must be: (1) expressed in brain regions relevant to ASD social behaviors and (2) expressed during the ASD susceptible window of brain development.

**Methods:**

Focusing on the amygdala, a brain region whose dysfunction has been highly implicated in the social component of ASD, we mined publicly available gene expression databases to identify ASD-susceptibility genes expressed during human and mouse amygdala development. We found that a large cohort of known ASD susceptibility genes is expressed in the developing human and mouse amygdala. We further performed analysis of single-nucleus RNA-seq (snRNA-seq) data from microdissected amygdala tissue from five ASD and five control human postmortem brains ranging in age from 4 to 20 years to elucidate cell type specificity of amygdala-expressed genes and their dysregulation in ASD.

**Results:**

Our analyses revealed that of the high-ranking ASD susceptibility genes, 80 are expressed in both human and mouse amygdala during fetal to early postnatal stages of development. Our human snRNA-seq analyses revealed cohorts of genes with altered expression in the ASD amygdala postnatally, especially within excitatory neurons, with dysregulated expression of seven genes predicted from our datamining pipeline.

**Limitations:**

We were limited by the ages for which we were able to obtain human tissue; therefore, the results from our datamining pipeline approach will require validation, to the extent possible, in human tissue from earlier developmental stages.

**Conclusions:**

Our pipeline narrows down the number of amygdala-expressed genes possibly involved in the social pathophysiology of ASD. Our human single-nucleus gene expression analyses revealed that ASD is characterized by changes in gene expression in specific cell types in the early postnatal amygdala.

## Background

As defined by the Diagnostic and Statistical Manual of Mental Disorders (DSM-5), autism spectrum disorder (ASD) is characterized by deficits in social communication and repetitive/restrictive behaviors [1]. Despite these two core defining features, phenotypes and comorbidities in individuals with ASD vary widely. This is likely due in large part to the complex etiology of ASD. Over recent years, a number of large-scale genomic sequencing efforts have been undertaken to uncover the genetic landscape of ASD [2–5]. To date, as cataloged by the Simons Foundation Autism Research Initiative (SFARI Gene) “(https://www.sfari.org/resource/sfari-gene/)”, there exists over 1000 genes linked to ASD. While the multigenic basis of non-syndromic cases of ASD is widely accepted, the underlying genetic load carried by each individual differs. Moreover, this genetic load likely represents a combination of both inherited, and to a large extent, de novo mutations [2–6]. Despite an emerging understanding of ASD genetics, the challenge remains how to identify which of these risk genes and mutations are associated with specific core symptoms.

Recent efforts by a number of groups have attempted to leverage this newly found genetic understanding of ASD to begin to parse the biological pathways, brain regions, and timing of gene expression linked to ASD. From these studies, a handful of convergent information has been distilled. First, using functional gene network analyses, perhaps not surprisingly, ASD genes are generally involved in various aspects of brain development, such as neuronal proliferation, cell migration, and synapse formation [7–9]. Second, by accessing information from relatively recently developed databases of temporal and spatial gene expression patterns in humans, analyses from a number of groups using different parameters and sets of input ASD genes have revealed that ASD gene expression is enriched in specific brain regions and during key developmental windows [10, 11]. Brain regions of interest most prominently include the cerebral cortex (notably frontal and temporal cortices), the amygdala, the cerebellum, and the striatum, with the overwhelming majority of ASD susceptibility genes expressed primarily during fetal to early post-natal stages of development [7, 10–15]. The timing of expression of ASD susceptibility genes during fetal development is in line with the early detection of core symptoms in individuals with ASD. Although the age at which ASD behavioral traits emerge varies, many of the signs, such as gaze aversion, are detectable by as early as 12 months [16, 17]. That early brain developmental deficits underlie ASD is further supported by postmortem studies of ASD brains which reveal cortical deficits explainable by alterations in prenatal development [18].

In addition to gene network analyses, more recent studies have taken advantage of gene expression profiling approaches, such as RNA sequencing (RNA-seq). These approaches have provided investigators with more precise tools to understand which gene expression patterns are altered in the brains of individuals with a variety of neurodevelopmental disorders, including ASD [13, 19, 20]. In ASD, these analyses have revealed consistent changes in both gene transcription and post-transcriptional RNA splicing. ASD gene expression changes appear to be biased toward genes/gene networks critical for maintaining neurotypical excitatory/inhibitory balance in neuronal communication. These include genes expressed in both inhibitory and excitatory neurons, as well as genes that regulate neuronal activity. Recently, gene expression analysis of ASD at the single-cell resolution using single-nucleus RNA sequencing (snRNA-seq) provided direct evidence of involvement of specific neuronal and glial subtypes, such as upper-layer cerebral cortical projection neurons [21].

While these studies have been critical for understanding how the molecular pathology of ASD affects the neocortex, how genetic perturbations affect neurodevelopmental trajectories of non-cortical ASD brain regions remains unknown. Here we focused on the amygdala, a structure whose altered function has been closely linked to ASD social phenotypes [22, 23], with the goal of establishing an “amygdala-developmental” list of genes whose potential expression is altered in ASD. To accomplish this, we combined the latest catalogs of ASD-associated genetic risk factors with the available human and mouse gene expression datasets from the BrainSpan project [http://hbatlas.org/] and Mouse Allen Brain Atlas [http://mouse.brain-map.org/]. We then leveraged our detailed knowledge of mouse pre- and early post-natal amygdala development to validate expression of human amygdala-expressed ASD genes and to provide amygdala subnuclei spatial resolution not available in current human databases. To test the predictability of this pipeline in human ASD tissue, we analyzed snRNA-seq data from postmortem amygdala tissue of individuals with ASD and matched neurotypical controls. Interestingly, despite the discrepancies in the ages of our pipeline (fetal stages) and human snRNA-seq analyses (first two decades of life), our pipeline predicted a cohort of genes whose expression is altered in the human amygdala in ASD. We further demonstrated that a majority of these human genes are expressed within distinct amygdala subnuclei in the mouse, implicating different components of the amygdala in ASD. Thus, by combining datamining with transcriptomic changes in the amygdala of individuals with ASD, our findings provide a new approach to potentially link complex gene expression patterns during neurodevelopment in ASD with later emerging social behavioral phenotypes.

## Methods

### Gene lists

To compile a list of ASD susceptibility genes, we used two datasets: the SFARI Gene list (https://gene.sfari.org/) and a recent list of germline mutations from the Autism Sequencing Consortium (gene list from Satterstrom et al., 2020 [5]). We utilized the most up to date and high confidence SFARI gene list (September, 2017). From the SFARI list, we only considered genes in category S (syndromic), 1 (high confidence), 2 (strong candidates), or 3 (suggestive evidence). This provided a total of 280 genes. The Satterstrom, et al., 2020 list represented 102 genes. We cross referenced these two lists, resulting in a total of 323 ASD-linked genes. Fifty-nine genes overlapped between the two datasets (results shown in Fig. 2).

### Human gene expression assessment

To investigate amygdala expression of this compiled list of 323 ASD-linked genes during human brain development (see Additional File 1), gene-level human brain expression data (Platform GPL5175, Affymetrix GeneChip Human Exon 1.0 ST Array), which were generated as part of the BrainSpan project (hbatlas.org) [24], were downloaded from the NCBI GEO database (accession number GSE25219) in the form of log_2_-transformed signal intensity values. The BrainSpan database collates data of gene expression levels in various regions of postmortem human brain that range in age from 5.7 post-conceptional weeks (PCW) to 82 postnatal years. Of the 323 genes, 295 were present in BrainSpan. To examine the amygdala expression levels, we made one expression value per sample by averaging each sample’s hemispheric values. To obtain an expression value per developmental period, we calculated the median expression value between all samples within that time period. We then calculated the average expression level across the developmental periods of interest. For the brain expression data, values > 6 in at least one sample are considered positive brain expression. We used Python, a high-level programming language to create a script (accessible at https://gist.github.com/saarthak24/8ffa73138258257e12002cfca6cb00b6), to parse the BrainSpan database and output only the ASD-related genes from the above list that fulfilled two criteria: (1) expressed in the amygdala anytime between early fetal (period 2, 8–10 PCW) to neonatal and early infancy (period 8, birth to 6 months, M) and (2) with a log2 signal index of greater than 6.0, which is considered positive gene expression [24]. A total of 271 genes out of 295 (91.9%) fulfilled these criteria.

### Mouse brain expression assessment

To investigate expression of the above list of 271 human amygdala-expressed ASD genes in the developing mouse brain, we datamined the Allen Developing Mouse Brain Atlas (http://developingmouse.brain-map.org), a detailed atlas of gene expression in the mouse as assessed by *in situ* hybridization, with temporal and region-specific expression of ~ 2000 genes functionally relevant to brain development or disorders of the brain. Of the 271 human amygdala-expressed ASD genes, 82 had data in the Allen Brain Atlas, the remaining 189 did not. We assessed these 82 genes in two phases: First, we conducted a broad regional assessment, including only genes that fulfilled all of the three following criteria: (1) using expression summary heat maps provided by Allen Brain expressed in either rostral secondary prosencephalon (RSP), telencephalic vesicle (Tel), and/or peduncular hypothalamus (PedHy), developing brain regions known to give rise to the amygdala, (2) an expression level of log 2 or higher (scale of log − 1.5 to log 3.5), and (3) expressed during at least one of five stages of development: embryonic day (E) 11.5, E13.5, E15.5, E18.5, or postnatal day (P) 4. Of the 82 genes, 80 (97.6%) met these criteria (Additional File 1). The second phase of assessment consisted of visual analysis of *in situ hybridization* images from the Allen Developing Brain Atlas at E11.5, E13.5, E15.5, E18.5, and P4 for each of the 80 genes that fulfilled the above criteria. Specifically, at each time point, all sections through the mouse brain were assessed for expression in developing brain regions previously identified by us and others [25–32] to give rise to different nuclei of the amygdala. At E11.5, E13.5, and E15.5, these regions consisted of the telencephalic/diencephalic border region, ventral hypothalamus, and ventral pallial (VP) region of the developing cerebral cortex. At E18.5 and P4, expression was assessed across the emerging amygdala nuclei, including the basolateral complex and the medial, cortical, and central nuclei. The level of expression—strong or scattered—was noted for each age (Additional File 1).

### Assessment of expression variance for the selected genes in the developing human amygdala

In order to estimate the variance of expression for the genes in the 271 and 80 gene lists, we obtained gene expression data from the human amygdaloid complex from PCW 8 to 40 years of age [33]. We then estimated variance in expression for all genes expressed in this dataset (mean expression > 0) and genes in the 271 and 80 gene lists.

### Heatmap

**Fig. 1:**
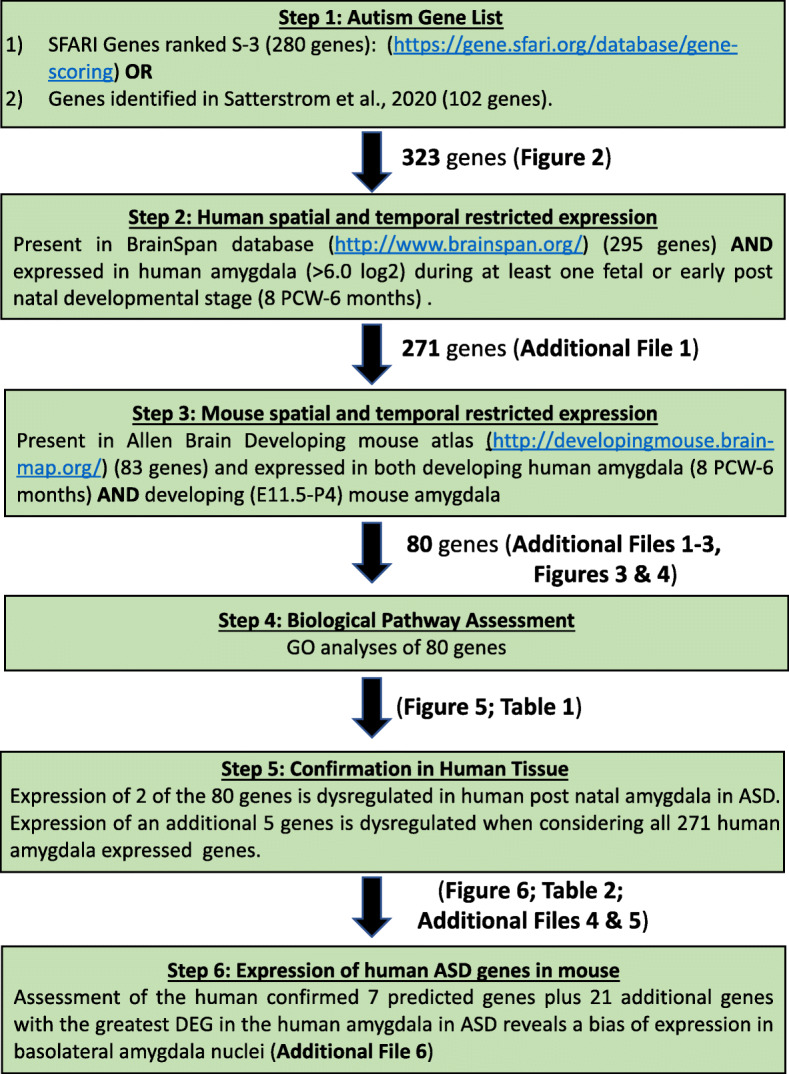
Each step in the pipeline to identify an ASD “amygdala-developmental” gene set. Description of each step in the pipeline describes the steps taken and figures, tables, and additional files where the results from each step are shown

Figure 4 was generated by heatmap.2 function in gplots package (version 3.0.3) along with RColorBrewer package (version 1.1.2) which provides color schemes for maps in R (version 3.5.2). The heatmap represents differences in expression over time of the 80 “amygdala-developmental” gene set from our pipeline (Fig. 1) genes based on the DESeq2 normalized gene expression with padj < 0.1. Rows correspond to genes and columns to the developmental periods defined in between early fetal (period 2, 8–10 PCW) and neonatal and early infancy (period 8, birth to 6 M). The genes with similar expression patterns are clustered together. The upregulated genes are in red, and the downregulated genes are in green.

### Gene ontology analysis

Gene ontology (GO) and functional analysis using the Ingenuity Pathway Analysis software (IPA, Qiagen vs. 2020), Genomatix (vs. 3.11), and the Cytoscape (vs. 3.5.1) plug-in ClueGO (vs. 2.5.5 ) was run in order to identify and plot the most prominent processes and pathways in which the 80 genes obtained with our pipeline are implicated. Genomatix function “GeneRanker” and ClueGO allowed characterization of gene sets and provided information on canonical pathways, biological processes, molecular functions, and diseases with a significant enrichment, listing the output with the respective *P* value by using annotation data from various sources like Gene Ontology or Genomatix proprietary annotation. *P* values < 0.05 were considered significant. We selected ClueGO to plot overrepresented biological processes with experimental evidence using very conservative thresholds (*P* values < 0.01, Kappa Score = 0.75, GO tree interval from 3 to 8), showing in the plot the related genes obtained with our pipeline (Fig. 5). Several of the most relevant processes and genes are summarized in Table 1.

**Table 1:** Enriched GO terms for the 80 ASD-genes expressed in amygdala

GO-Term	*P* value	List of observed genes
Telencephalon development	2.33E−17	ARX, AVPR1A, CTNNB1, FOXP2, GRIN1, LAMB1, NF1, PAX6, PLXNA4, PTEN, RELN, SLC1A2, TBR1, TSC1
Pallium development	2.39E−14	ARX, CTNNB1, FOXP2, GRIN1, LAMB1, NF1, PAX6, PTEN, RELN, TBR1, TSC1
Forebrain neuron differentiation	1.06E−04	ARX, PAX6, TBR1
Neural crest differentiation	2.37E−07	CTNNB1, GFAP, HDAC4, SOX5, TCF4
Regulation of behavior	2.24E−11	AHI1, CNR1, CNTNAP4, HDAC4, MTOR, NRXN1, RELN
Synaptic transmission, GABAergic	1.17E−10	CNR1, CNTNAP4, GABRB2, NF1, PTEN, SLC6A1
Ionotropic glutamate receptor activity	3.10E−11	GRIA2, GRIK2, GRIK5, GRIN1, GRIN2B, NRXN1, RELN
Synaptic transmission, glutamatergic	3.89E−14	CNR1, GRIA2, GRIK2, GRIK5, GRIN1, NF1, NRXN1, PRKN, RELN
Regulation of postsynaptic membrane potential	1.77E−14	CHRNA7, GABRB2, GABRB3, GRIK2, GRIK5, GRIN1, GRIN2B, NRXN1, PTEN, RELN
Positive regulation of axonogenesis	3.45E−07	ADNP, DSCAM, PLXNA4, PLXNB1, SEMA5A
Regulation of dendritic spine development	8.78E−06	FMR1, MTOR, PTEN, RELN
Regulation of neurotransmitter uptake	4.09E−06	GFAP, PER2, PRKN
Androgen receptor signaling pathway	6.28E−06	CREBBP, CTNNB1, PTEN, UBE3A
Dopamine transport	8.90E−05	CNR1, PRKN, SLC6A3
Corticotropin-releasing hormone signaling pathway	6.85E−06	CACNA1H, CTNNB1, PRKCB, TCF4
Multicellular organismal response to stress	2.61E−07	DEAF1, GRIK2, GRIK5, PTEN, RELN
Regulation of transcription regulatory region DNA binding	2.20E−06	CTNNB1, NSD1, PAX6, PER2
Circadian entrainment	3.41E−09	CACNA1H, GRIA2, GRIN1, GRIN2B, PER2, PRKCB
Wnt signaling pathway	4.50E−15	CTNNB1, MTOR, PRKCB, TCF4, TCF7L2, TEK, TSC1, TSC2
Epithelial cell apoptotic process	3.52E−08	DNMT3A, MTOR, SEMA5A, TCF4, TCF7L2, TEK
Long-term synaptic potentiation	9.54E−09	CHRNA7, GFAP, GRIN2B, NF1, PTEN, RELN

Selected terms and related metrics were reported using ClueGO. All terms had a *P* value < 0.10

### Annotation of human amygdala cell type signatures based on snRNA-seq data

To assess gene expression in human ASD probands, we interrogated a previously published dataset of 12,289 single nucleus RNA sequencing (snRNA-seq) profiles captured from postmortem amygdalar tissue of five ASD and five age- and sex-matched neurotypical controls [34]. The dataset contained 15 cell clusters, which we further annotated based on expression of known markers of neuronal and glial cell types (Fig. 6). In order to obtain a list of the enriched genes in each cell type, we performed marker analysis by comparing snRNA-seq profiles in each cluster to nuclei in all other clusters using MAST [35].

### Identification of ASD-associated differentially expressed genes in human amygdala cell types

Identification of genes differentially expressed in ASD compared to control in each amygdala cell type was performed as described previously [34]. Briefly, MAST was used to fit a linear mixed model (LMM) using nuclei profiles from each cluster and account for ASD diagnosis, age, sex, RNA integrity number (RIN), and postmortem interval, as well as to account for the fact that multiple nuclei were captured from each individual. To identify genes whose differential expression is associated with the ASD diagnosis in each cell type, likelihood-ratio test was performed by comparing the model described above to the model excluding the diagnosis factor. Genes with at least 10% gene expression level difference between ASD and control and FDR < 0.05 were considered differentially expressed. We additionally ran MAST with only the diagnosis factor in the model and filtered genes with fold change < 10% based on fitting this simplified model. This analysis identified 190 differential gene expression events across 15 cell types that were detected in 183 individual genes (Additional File 4, Fig. 6). Then, in order to identify which amygdala cell types human amygdala ASD genes are enriched in, we overlapped the lists of genes dysregulated in ASD in specific amygdala cell types with the list of 80 mouse/human and 271 human amygdala ASD genes (Additional File 5).

### Visualization of snRNA-seq data

R and ggplot2 package were used for snRNA-seq data visualization. To visualize expression of genes across cell types, normalized Unique Molecular Identifier (UMI) counts for the genes of interest in each cell were plotted on the cluster map. To visualize levels of differentially expressed genes in ASD and control nuclei in a specific cell type, normalized UMI counts were plotted as a violin plot in the ASD and control group.

## Results

### Pipeline analysis

**Fig. 2:**
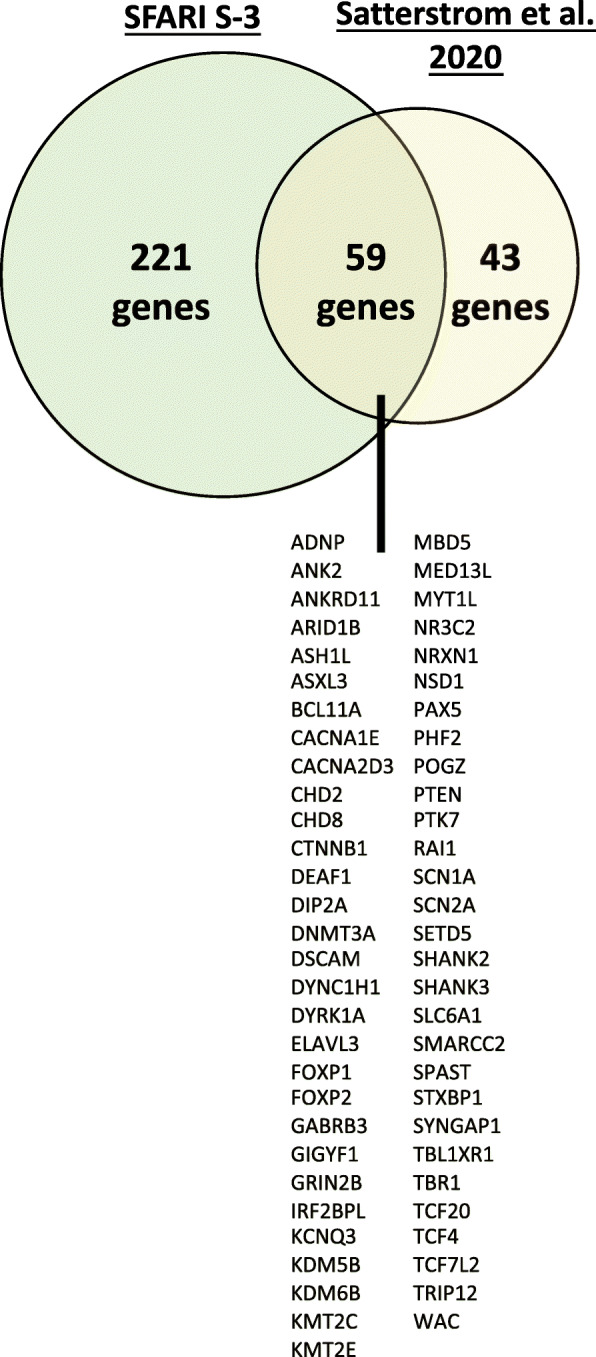
Venn Diagram representation of ASD datasets utilized. There is overlap of 59 genes between the datasets of SFARI ASD genes ranked S-3 and a recent list of germline mutations from the Autism Sequencing Consortium (Satterstrom et al. 2020). This overlap of 59 genes is highly significant (*p* value = 6 × 10^112^)

The first step in our pipeline was to compile a list of ASD susceptibility genes (pipeline workflow shown in Fig. 1). To accomplish this, we used two major ASD gene sets: the SFARI human gene list (https://gene.sfari.org/) collated by the Simons Foundation and the to date largest exome sequencing study of individuals with ASD (referred to herein as the “Satterstrom dataset”) [5]. The SFARI gene list represents the most comprehensive and up-to-date database of known autism susceptibility genes. SFARI scores genes according to their evidence toward contribution to ASD on a scale of S (syndromic) to 6 (evidence does not support a role) (see https://gene.sfari.org/about-gene-scoring/criteria/ for scoring criteria). We focused only on higher confidence genes scored between S (syndromic) and 3 (suggestive evidence). This represented 280 genes. The Satterstrom dataset consisted of 102 genes. Combining these two gene sets, we established a list of 323 genes with 59 genes overlapping between the two datasets (Fig. 2; Additional Figure 1).

**Fig. 3:**
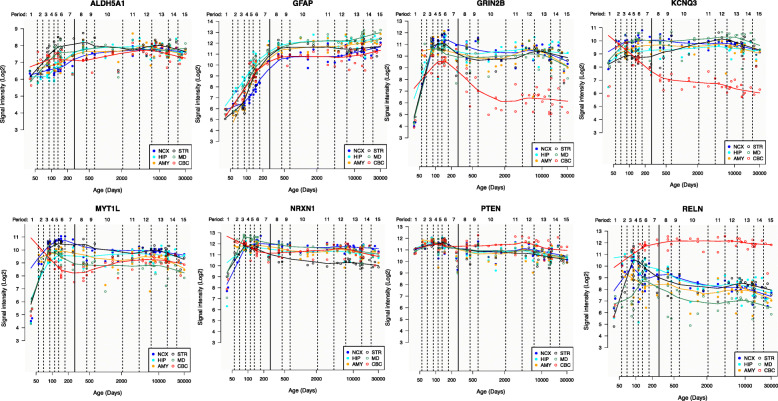
Plots of dynamic expression of 10 ASD genes in the human brain. Ten examples of the 271 ASD genes. Gene-level brain expression graphs were generated with data from the *BrainSpan* project (Kang et al. 2011) collected from postmortem human brain samples. All genes are expressed in the human amygdala (AMY) as well as the neocortex (NCX), Striatum (STR), Hippocampus (HIP), mediodorsal nucleus of the thalamus (MD), and cerebellar cortex (CBC). The x-axis shows age with the dark vertical line signifying birth between periods 7 and 8. Along the y-axis are values of median expression (Log2-Transformed Signal Intensity) for which values ≥ 6 signify high levels of expression in at least one postmortem brain sample

We next wanted to assess whether this combined 323 gene dataset is expressed in the human amygdala during the ASD susceptible window of early fetal development to 6 months postnatal age [9–11]. To accomplish this, we datamined postmortem human gene expression data from the BrainSpan project (http://hbatlas.org/) [36] from human fetal stages (8–10 PCW) through early life (0–6 months). From the 323 combined gene list, 295 had data in the BrainSpan database. Datamining the BrainSpan database, we found that 271 of the 295 gene set (91.9%) are expressed in the human developing amygdala during at least one of the human developmental stages examined (Additional File 1; Fig. 3). In addition, by analyzing bulk tissue RNA-seq from developing human amygdala [34], we observed that the 271 human amygdala genes have higher variance of expression compared to all genes expressed in the human amygdala (16 for the 271 genes compared to 4.6 for all expressed genes). This observation further suggested that the genes we identified are dynamically expressed during human amygdala development.

While the BrainSpan database is a highly valuable resource for assessing dynamic gene expression patterns through the human lifespan, the resolution of tissue identity comprising a specific brain region, especially during fetal stages, maybe limited and imprecise. This can be overcome in part by assessment of gene expression in animal models, especially the mouse, where there currently exist very high resolution gene expression atlases of the developing brain, with the Allen Developing Mouse Brain Atlas (http://developingmouse.brain-map.org/) the premier of these. Furthermore, considering the conservation of the majority of brain developmental processes across higher vertebrate species, the gene expression patterns found in the Allen Developing Mouse Brain Atlas can likely be leveraged to provide a precise and detailed level of understanding of exactly where in the human developing brain genes of interest are expressed.

### Gene expression analysis

**Fig. 4:**
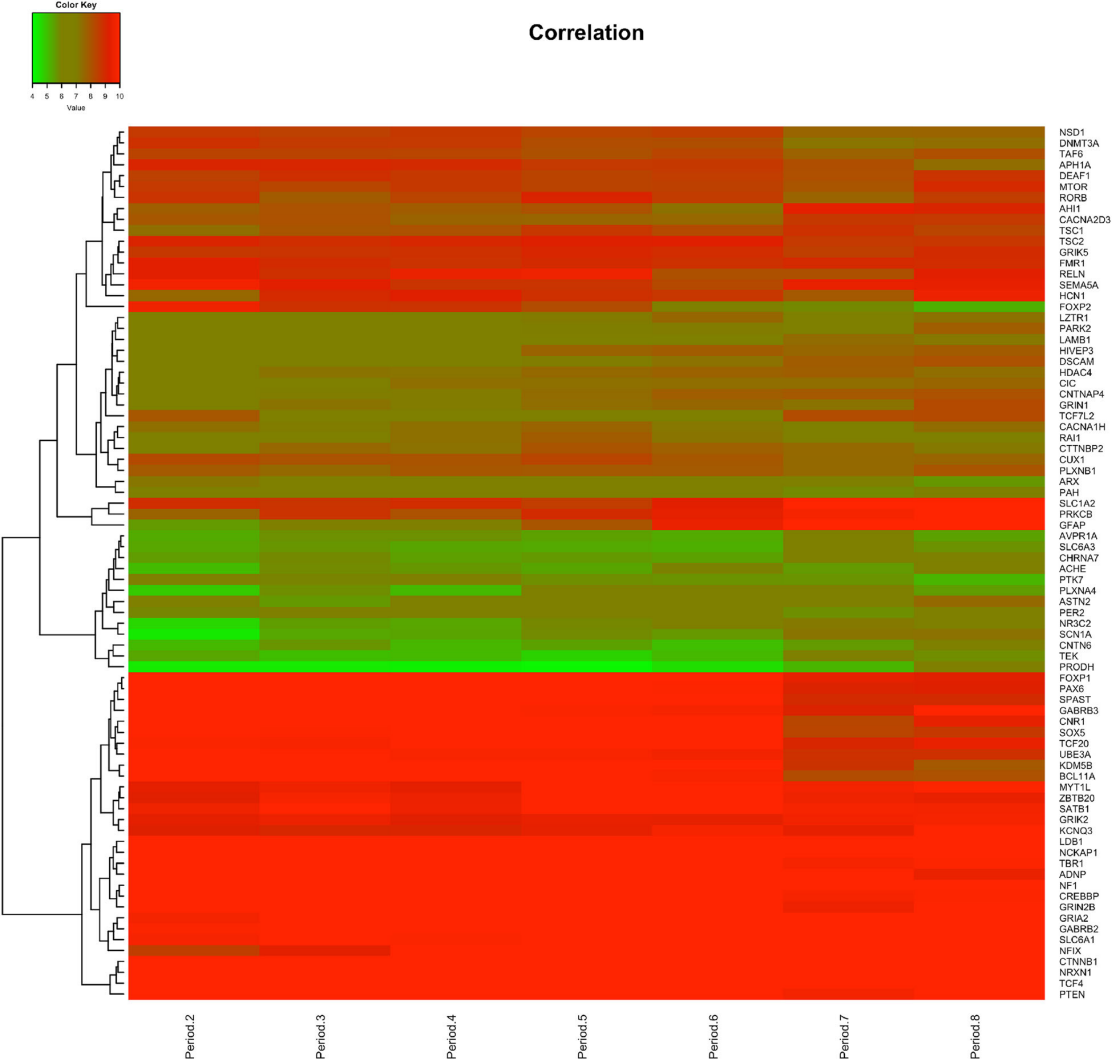
Heat map of amygdala expression in the human brain of the 80 genes identified as the “amygdala-developmental” gene set. Period of development is shown in the x-axis, with gene names of all 80 genes on the y-axis on the right. The majority of genes maintained similar levels of expression throughout development, while others were dynamic

A number of studies over recent years by us and others [25–32] have revealed the developmental origin of the majority, if not all, cells that will populate the amygdala. This work has revealed that amygdala cells derive from progenitor zones located in distinct subcompartments (niches) of the embryonic telencephalon and diencephalon. These progenitor zones include regions of the developing brain termed as follows: rostral secondary prosencephalon (RSP), telencephalic vesicle (Tel), and/or peduncular hypothalamus (PedHy) (Additional File 2). Based on this criteria, we datamined the Allen Developing Mouse Brain Atlas and found that of the 271 human amygdala-expressed genes, data for 189 genes were not available in the Allen Brain database (Additional File 1). Of the 82 genes that were found in the Allen Developing Mouse Brain Atlas, 80 genes (97.6%) were expressed in the developing brain regions in mouse that contribute to the amygdala or in the developing amygdala itself at either E11.5, E13.5, E15.5, E18.5, or P4, ages corresponding to human mid fetal to early postnatal stages (Additional File 1 and 3**)**. We further show that expression of many of these 80 genes undergoes dynamic changes during human fetal to early stages of postnatal of amygdala development, coinciding with the ASD susceptible window of development (Fig. 4). Indeed, as with the 271 human amygdala genes, expression of the 80 genes varied more during human amygdala development compared to all expressed genes (variance 18.1 for the 80 genes vs 4.6 for expressed genes). Thus, having passed through the filters of expression in both the human and mouse developing amygdala, we consider these 80 genes as a two species validated “developing amygdala ASD-susceptibility gene set.”

### Biological pathway analysis

**Fig. 5:**
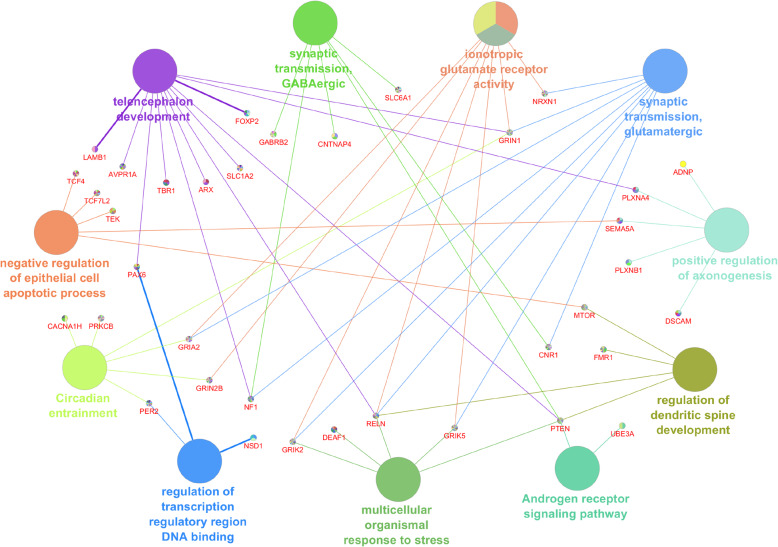
Interactomes of top GO terms. Top gene networks of the 80 genes obtained by our pipeline include a variety of biological and developmental processes. Some of the genes were common to different GO processes and pathways; thus, deficiencies in any of these genes could cause multiple effects

We next wanted to assess whether these 80 genes are preferentially associated with specific biological pathways and are expressed in specific neuronal cell types. To assess biological pathways, we performed functional and GO enrichment analysis and found genes within a number of pathways related to different aspects of brain development including excitatory and inhibitory synaptic transmission and hormonal control (androgen, corticotropin-releasing hormone) (Fig. 5, Table 1).

### Human RNAseq

**Fig. 6:**
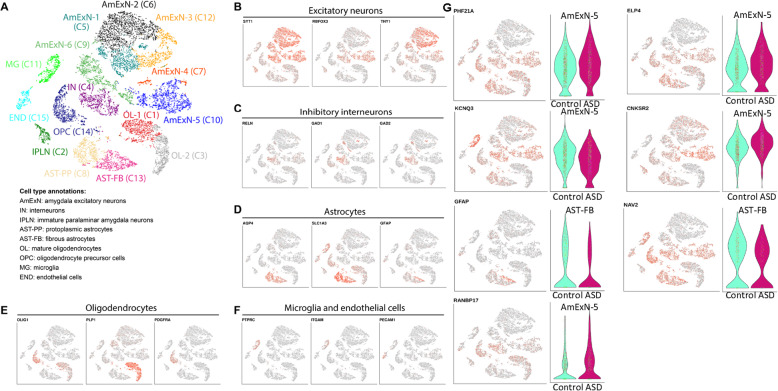
snRNA-seq analysis of ASD-associated gene expression changes in the cell types of human amygdala. **a** Cell types in the human amygdala identified based on unbiased clustering of single nucleus transcriptional profiles. **b**–**f** Feature plots for markers of excitatory neurons (**b**), interneurons (**c**), astrocytes (**d**), cells of oligodendrocyte lineage (**e**), as well as microglial and endothelial cells (**f**). **g** Genes differentially expressed in ASD amygdala in a cell type-specific manner. Violin plots display expression of genes that were significantly dysregulated in ASD compared to control in a particular amygdala cell type. Feature plots show expression of the same genes across all cell types

One expectation is expression of genes of interest would be dysregulated in the amygdala in individuals with ASD. To study this, as well as examine amygdala gene expression changes in the human ASD amygdala, we analyzed a single-nucleus RNA sequencing (snRNA-seq) dataset generated from dissected human amygdala tissue from five individuals with ASD ranging in age from 4 to 20 years and five age- and sex-matched neurotypical controls [34]. This analysis yielded 183 genes dysregulated in the ASD amygdala compared to controls containing 15 unbiased cell clusters representing different cell types of the human amygdala (Additional File 4, Additional File 5, and Fig. 6). We annotated each of the clusters by performing unbiased marker gene identification (see “Methods” section) and interrogating known markers of neuronal and glial cell types. We then performed differential gene expression analysis by comparing nuclei profiles from the ASD and control samples in each cluster (see “Methods” section) and found that the majority of these genes are expressed in excitatory neuron clusters (Additional File 5 and Fig. 6).

Comparing the snRNA-seq data of human amygdala genes dysregulated in ASD with our human/mouse, we found that two of the 80 genes (*GFAP* and *KCNQ3*) had altered expression in individuals with ASD (Table 2 and Fig. 6). When we expanded the dataset to include the entire 271 autism susceptibility genes that are expressed in the human amygdala, we found an additional five genes (*PHF21A*, *RNABP17*, *ELP4*, *CNKSR2*, and *NAV2*) with altered expression in individuals with ASD (Table 2 and Fig. 6). We further assessed which of the 15 unbiased cell clusters representing different cell types of the human amygdala contained most of the 80 and 271 gene datasets. We found that genes in both of these datasets were enriched in excitatory neurons (clusters C7), fibrous astrocytes (cluster 13), followed by OPCs (cluster 14), and interneurons (C4). Genes from the larger dataset of 271 genes were most enriched in the same cell types (Additional File 5). This suggests that the genetic burden of ASD has the potential to impact multiple cell types in the amygdala, predominantly excitatory neurons but also inhibitory neurons, oligodendrocyte precursors, and astrocytes.

**Table 2:** Detailed information of 7 genes identified from our human amygdala snRNA-seq screen and found in the 80 (GFAP, KCNQ3) and 271 (all 7) amygdala gene lists

Symbol	Cluster	Cluster ID	Description	Additional references
GFAP	**C13**	Fibrous astrocytes	**Glial Fibrillary Acidic Protein** encodes one of the major intermediate filament proteins of mature astrocytes. It is used as a marker to distinguish astrocytes from other glial cells during development. Mutations in this gene cause Alexander disease, a rare disorder of astrocytes in the central nervous system. Involved in responses to brain injury and disease states.	Edmonson et al., 2014. Altered glial marker expression in in autistic post-mortem prefrontal cortex and cerebellum. *Molecular Autism.*
KCNQ3	**C10**	Excitatory neurons	**Potassium Voltage-Gated Channel Subfamily Q Member 3** gene encodes a protein involved in the regulation of neuronal excitability and responsiveness to synaptic inputs by contributing to the slow component of synaptic AHP (afterhyperpolarization) that determines the firing pattern of a neuron. Diseases associated with KCNQ3 include seizures, Benign Familial Neonatal convulsions type 2, and Benign Familial Neonatal Epilepsy.	Sands et al., 2019. Autism and developmental disability caused by KCNQ3 gain-of-function variants. *Ann Neurol.*
PHF21A	**C10**	Excitatory neurons	**PHD Finger Protein 21A** gene encodes BHC80, a component of a BRAF35 (MIM 605535)/histone deacetylase (HDAC; see MIM 601241) complex (BHC) that represses transcription of neuron-specific genes in non-neuronal cells through the cis-regulatory element known as repressor element-1 (RE1) or neural restrictive silencer (NRS). Disorders associated with PHF21A include Potocki-Shaffer Syndrome and Parietal Foramina.	Kim et al., 2019. Disruption of PHF21A causes syndromic intellectual disability with craniofacial anomalies, epilepsy, hypotonia, and neurobehavioral problems including autism. *Molecular Autism.*
RANBP17	**C10**	Excitatory neurons	**RAN Binding Protein 17** is a nuclear pore-associated transport receptor, member of the importin-beta superfamily. The small GTPase RAN plays a key role in the import of proteins with a nuclear localization signal (NLS). RANBP17 expression decreases with aging and in human fetal brain exhibited higher expression in females. Lower expression may be linked to altered nuclear pore function.	Mertens et al., 2015. Directly reprogrammed human neurons retain aging-associated transcriptomic signatures and reveal age-related nucleocytoplasmic defects. *Cell Stem Cell.*
ELP4	**C10**	Excitatory neurons	**Elongator Acetyltransferase Complex Subunit 4** encodes a component of the elongator complex, a histone acetyltransferase complex that associates directly with RNA polymerase II during transcriptional elongation. Elongators may play a role in chromatin remodeling and regulate the maturation of cortical projection neurons. This gene has also been associated with Rolandic epilepsy.	Reinthaler et al., 2014. Analysis of ELP4, SRPX2, and interacting genes in typical and atypical rolandic epilepsy. *Epilepsia.*
CNKSR2	**C10**	Excitatory neurons	**Connector Enhancer of Kinase Suppressor of Ras 2** encodes a synaptic protein that functions as a scaffold in the postsynaptic density (PSD). CNKSR2 regulates Ras signaling, which controls neuronal proliferation, migration, differentiation, apoptosis and synaptogenesis. Diseases associated with CNKSR2 include: mental retardation, language deficits, attentional problems/hyperactivity, and brief childhood epilepsy.	AK Vaags, S Bowdin, ML Smith et al., 2014. Absent CNKSR2 causes seizures and intellectual, attention, and language deficits. *Ann Neurol.*
NAV2	**C13**	Fibrous astrocytes	**Neuron Navigator 2** encodes a member of the neuron navigator family. Highly expressed in fetal and adult brain, Nav2 is a retinoic acid-responsive gene involved in nervous system development. Nav2 may play a role in cellular growth, migration and neuronal development of different sensory organs. It possesses helicase activity and exonuclease activity. Diseases associated with NAV2 include neuroblastoma.	Marzinke MA, Mavencamp T, Duratinsky J, Clagett-Dame M. 2013. 14-3-3ε and NAV2 interact to regulate neurite outgrowth and axon elongation. *Arch Biochem Biophys*.

We next wanted to assess the extent to which these genes whose expression is dysregulated in the human ASD amygdala are expressed in the mouse amygdala and in which nuclei. To accomplish this, we again utilized the Mouse Allen Brain Atlas, which provides high resolution gene expression data within specific amygdala subnuclei (http://mouse.brain-map.org/). Of the 183 genes dysregulated in the human ASD amygdala identified by snRNA-seq, we selected 27 genes for this detailed expression analysis. Seven of these genes were found in the 271 gene dataset (Table 2), and the remaining 20 represented a sampling of genes across clusters with the greatest DEG in human ASD. We found most of these genes are expressed in the mouse amygdala, with the majority expressed in the basolateral complex of amygdala nuclei and to a lesser extent in the medial amygdala nucleus (Additional File 6).

## Discussion

In the overwhelming majority of cases, ASD results from a complex mix of genetic mutations which give rise to neuroatypical trajectories of brain development [2–4, 6, 9, 37]. The advent of next-generation sequencing technologies over recent years has led to a deeper understanding of the genetic landscape of ASD, resulting in, to date, the identification of scores of susceptibility genes with varying contribution to ASD “(https://www.sfari.org/resource/sfari-gene/)”. Gaining a genetic and biological handle in ASD is an extremely daunting task, considering both complexity of the underlying genetics and that currently diagnosis is solely based on behavioral traits, criteria which also change with updates to the DSM. Additionally, while ASD is defined by shared behavioral traits [1], each individual with ASD is unique in the presentation of their disorder [2]. Thus, there exists a highly pressing need to understand and classify ASD by biological criteria. Central to this understanding is to develop methodologies and approaches that link an individual’s genotype to their unique ASD behavioral phenotype. This is reliant on detailed knowledge as to where and when ASD susceptibility genes are expressed in both the human brain and in animal models of ASD and brain development.

In this study, we performed cross species human and mouse gene expression analyses by first mining two of the most utilized and validated resources for human and mouse gene expression (BrainSpan and the Allen Brain Atlas), respectively, to develop a deeper understanding of the expression patterns of ASD susceptibility genes in the developing amygdala, a highly relevant brain region in relation to ASD social phenotypes. It is important to note that there are numerous other newly generated gene expression databases that can also be leveraged [38–40]. However, many of these do not have the same spatial resolution as the Allen Brain Atlas. The advent of more precise fetal human brain gene expression maps and expression data on primate brain sections should be very informative for revealing fine detailed anatomical localization of disease-related genes in the developing and mature human brain.

Employing state of the art single-cell genomics approaches, we further assessed gene expression patterns in human ASD tissue and, to our knowledge, uncovered for the first time changes in gene expression in the amygdala in ASD. Using these approaches, we reveal three major novel and impactful findings: (1) the majority of high ranking ASD susceptibility genes are expressed in the developing human amygdala, and via cross-species analyses these genes are also expressed in regions of the developing mouse brain that generate amygdala neurons, (2) the early postnatal human amygdala in ASD is characterized by dynamic changes in gene expression, and (3) that amygdala excitatory neurons appear to be a major site of expression of ASD susceptibility genes, and dynamic gene expression changes in the human ASD amygdala.

The rationale for our focus on the amygdala stems from a combination of evidence in both humans and animal models implicating this complex brain structure as critical for regulating social-emotional responses and processing of sensory stimuli with social relevance, processes which are disrupted in ASD. Consistent with this critical role in information processing, the amygdala has emerged as a key site of dysfunction in ASD as evidenced by cellular, structural, and functional imaging studies [22, 23, 41–44]. For example, fMRI studies reveal altered amygdala activation in face recognition and/or in response to social or emotional cues in individuals with ASD [45–48]. Thus, the amygdala is one of a likely handful of brain regions that define the “where” in ASD. Network and gene expression analyses of ASD-susceptible genes have also helped to define the “when” in ASD. These studies have strongly pointed to stages of mid-late fetal development as the major window of susceptibility for altered trajectories of brain development leading to ASD [8, 10–15]. Here, we find that, perhaps not surprisingly, a large number of high ranking ASD-susceptibility genes are expressed in the developing amygdala in humans. We initially hypothesized that a number of these genes may be expressed exclusively in the amygdala, which would help to explain why specific brain regions are affected in ASD, while others are spared. In contrast to this expectation, by parsing the human gene expression databases, we found that all of these are expressed across multiple brain regions without specificity for the developing amygdala. Thus, region-specific gene expression patterns of ASD susceptibility genes are likely not a mechanism that leads to region-specific deficits in ASD.

Our snRNA-seq analysis of ASD amygdala revealed a relatively large gene set (183 genes) with altered expression compared to control. This data provides, to our knowledge, the first insight into gene expression changes in the amygdala in ASD, revealing a putative strong role for dysfunction of amygdala excitatory neurons. If validated in larger cohorts, these gene expression changes may provide a genetic signature for understanding how altered gene function in relevant brain regions may be causal for behavioral deficits. It is important to note that, as our tissue samples were from postnatal humans (ages 4–20 years), these altered gene expression patterns may or may not represent causal changes occurring during fetal development, but instead may represent homeostatic compensatory mechanisms. Such changes may reflect a common mechanism across disorders to compensate for initial genetic and/or environmental insults [49, 50]. In addition, our snRNA-seq approach represents a tradeoff between cell type specificity of analyzing gene expression changes in ASD and sample size. Other approaches, such as bulk tissue RNA-seq and transcriptome-wide association analysis (TWAS) [51, 52] are able to profile larger number of samples but either do not possess single-cell resolution of snRNA-seq or did not analyze changes in the brain of ASD patients directly. As single-cell RNA-seq becomes more scalable and cheap and more ASD postmortem tissue samples are banked, we expect that the sample size used in such studies as ours will expand and will incorporate use of complementary approaches, such as TWAS. We also expect that a number of computational and/or machine learning approaches can be leveraged to tackle the complexity of genotype-phenotype correlations [53, 54].

Our finding of the majority of dysregulated genes to be in excitatory neurons is particularly intriguing. Excitatory/inhibitory imbalance in neuronal communication in ASD is well supported by studies in animal [48–50] and in vitro models derived from human tissue [55]. Though whether this imbalance is causal or compensatory remains to be resolved. The amygdala is comprised of numerous subtypes of excitatory neurons, which receive input from a variety of brain regions, including the prefrontal cortex and output to other limbic system nuclei such as the hypothalamus and bed nucleus of the stria terminalis [56]. In animal models, these input/out pathways through amygdala excitatory neurons have been shown to be key mediators of modulating social-emotional and anxiety-like behaviors [57–59]. Thus, dysfunction of amygdala excitatory neurons may underlie ASD behavioral phenotypes. Interestingly, deficits in cerebral cortical excitatory neurons in ASD have also been hypothesized to be a central component in ASD [5, 11, 12, 21].

## Limitations

1. We were limited by the ages in which we were able to obtain postmortem human tissue; therefore, the results from our datamining pipeline approach will require validation, to the extent possible, in human tissue from developmental stages.
2. Our human snRNA sequencing experiments were from five individuals with ASD and five age-matched controls across ages 4–20 years. These numbers were limiting and therefore validation in larger cohorts will be necessary as well as more samples at a given age.

## Conclusions

In summary, in this study, we leveraged the ease of access of existing databases of gene expression in both human and mouse to begin to understand the dynamic gene expression patterns of ASD susceptibility genes during development of the amygdala. From this analysis we provide a list of genes whose dysfunction may contribute to the social pathophysiology in ASD. We further link these findings to data obtained from snRNA-seq analysis of the human ASD amygdala and preliminarily establish that our datamining pipeline may provide a level of predictability for genes whose expression is altered in ASD. We believe that our approach will serve as a template for ongoing efforts to establish genotype-to-phenotype correlations in complex disorders such as ASD.

## Supplementary information

**Additional file 1.** List of genes.

**Additional file 2.** Regions of interest.

**Additional file 3.** Expression of 10 select genes.

**Additional file 4.** DEG human autism amygdala.

**Additional file 5.** Overlap between the 271 amygdala candidate ASD genes and markers of human amygdala cell types.

**Additional file 6.** Human genes in Allen Brain.

## Data Availability

All data generated or analyzed during this study are included in this published article [and its supplementary information files]. To parse the BrainSpan database we created a script (accessible at https://gist.github.com/saarthak24/8ffa73138258257e12002cfca6cb00b6).

## Abbreviations

ASD: Autism spectrum disorder
DEG: Differential gene expression
snRNA-seq: Single nucleotide RNA sequencing
