# Supplementary material for: Identification of amygdala-expressed genes associated with autism spectrum disorder
Source: Mol Autism. 2020 May 27;11:39. doi: 10.1186/s13229-020-00346-1 (PMC7251751; doi:10.1186/s13229-020-00346-1)
Supplement: Supplementary file 2 — Additional file 2. Regions of interest. [file 13229_2020_346_MOESM2_ESM.docx]

In-Situ Amygdala Expression Regions of Interest

| NOTE: The regions of interest designated as “Amygdala Progenitor Zones” vary across ages but can include the POA, TelA, LPall, VPall,and CSPall. | | | |
| --- | --- | --- | --- |
| Age | Region | Allen Map (Sagittal) | In-Situs (Sagittal) |
| E11.5 | Overview | 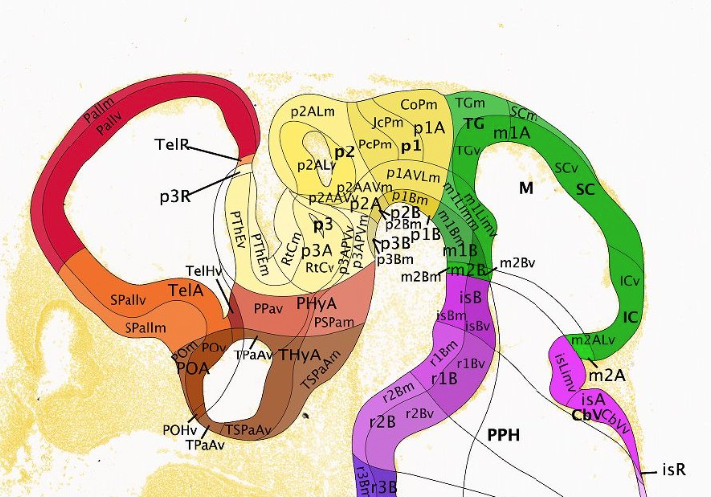 | 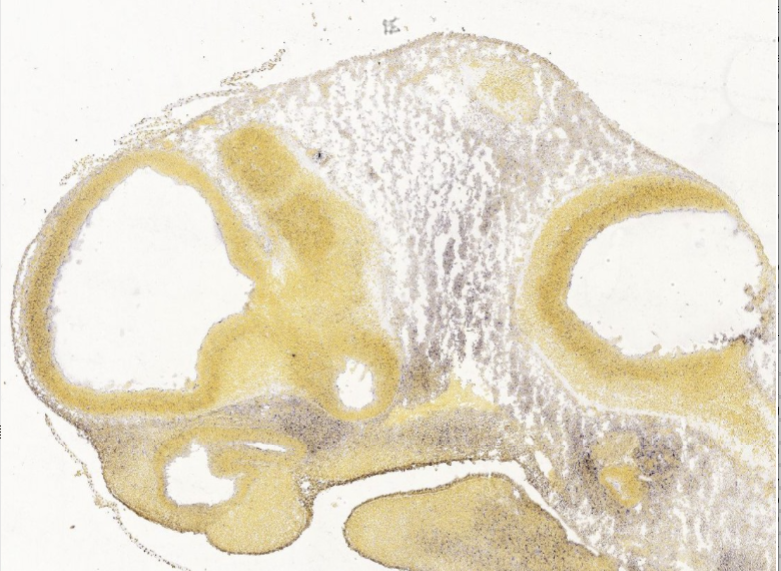 |
|  | POA | 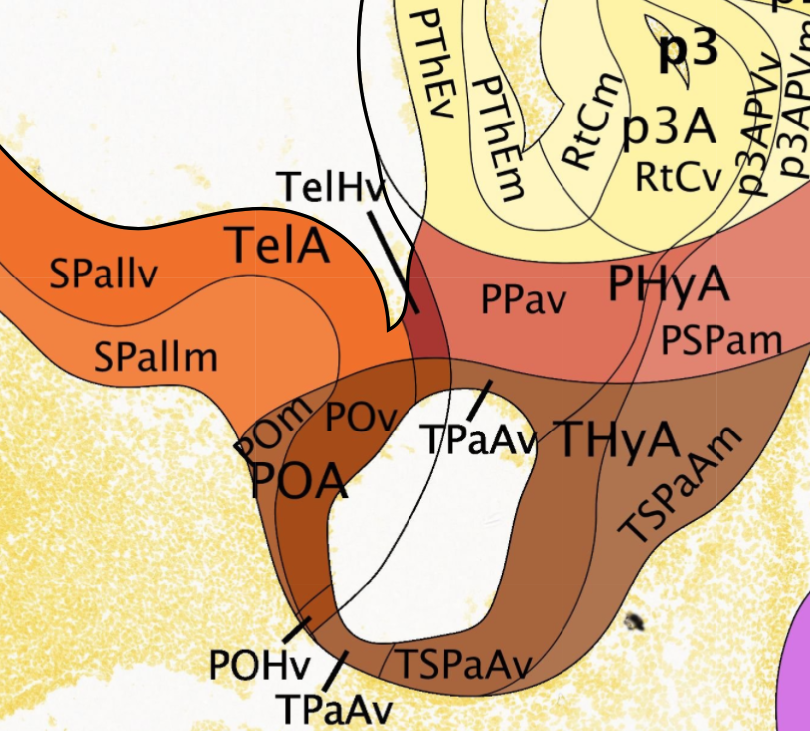 | 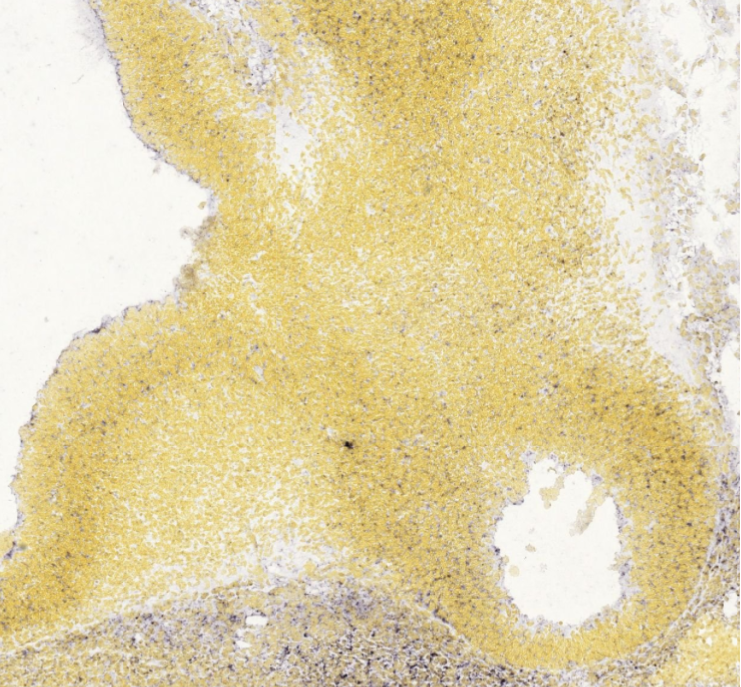 |
|  | TelA | 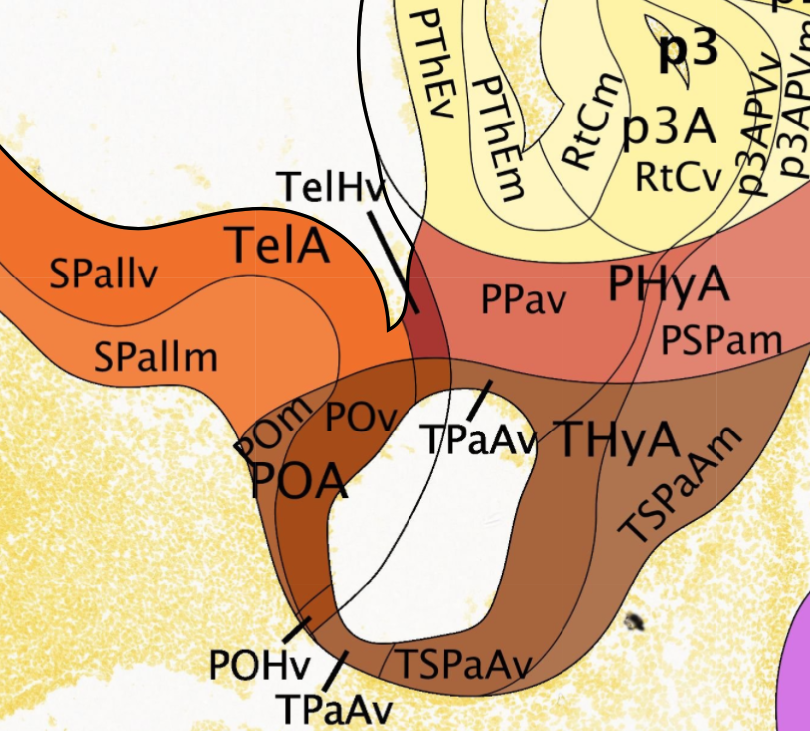 | 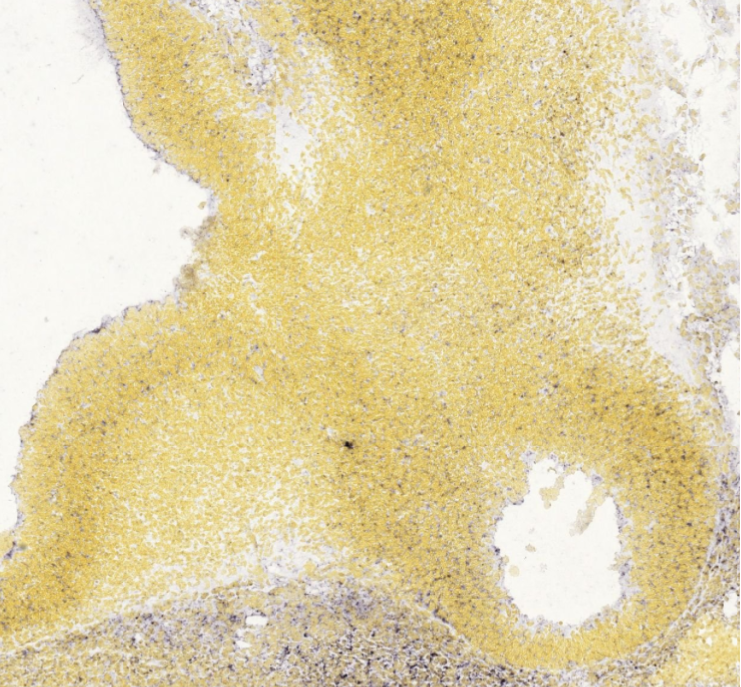 |
| E13.5 | Overview | 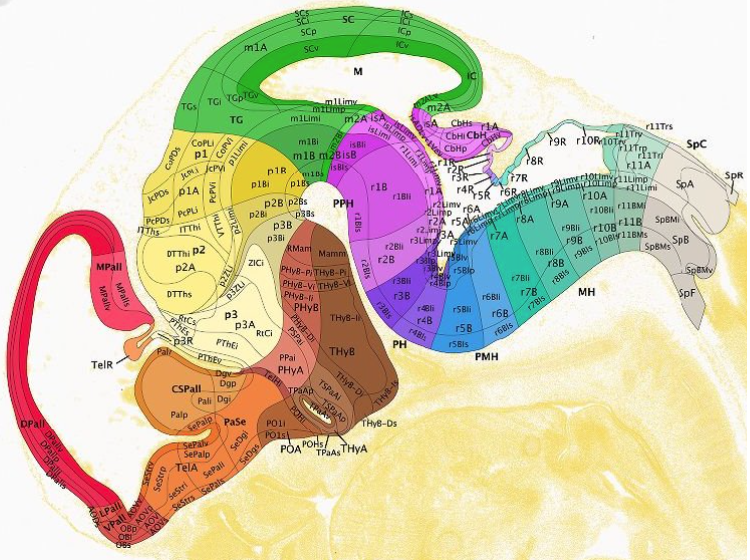 | 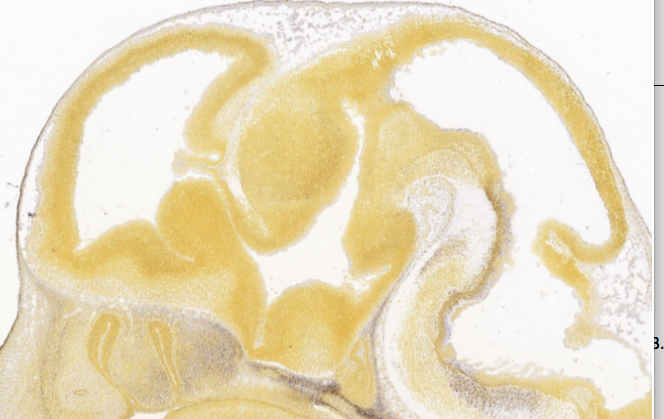 |
|  | POA | 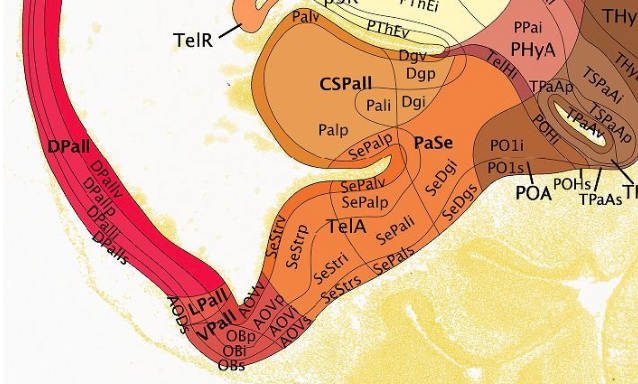 | 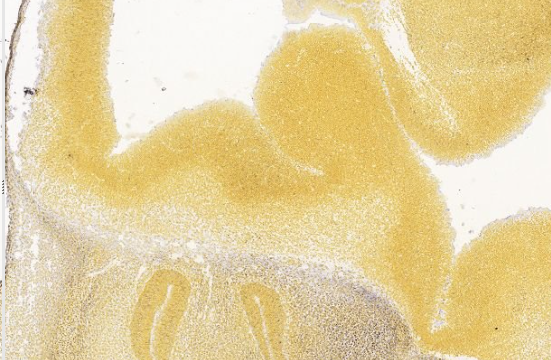 |
|  | TelA | 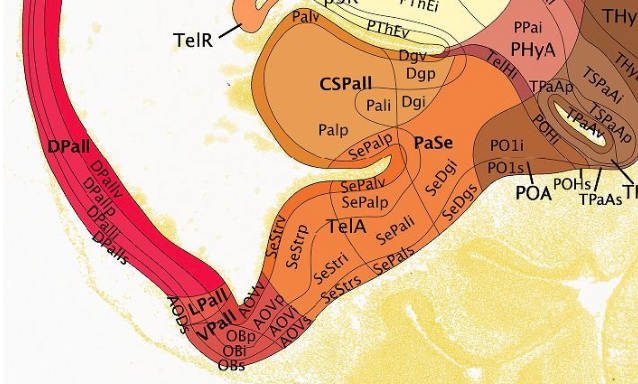 | 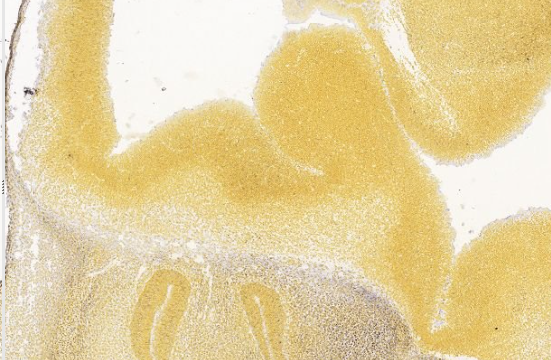 |
|  | LPall | 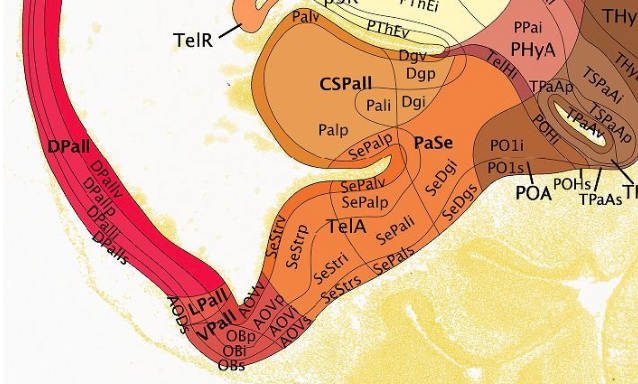 | 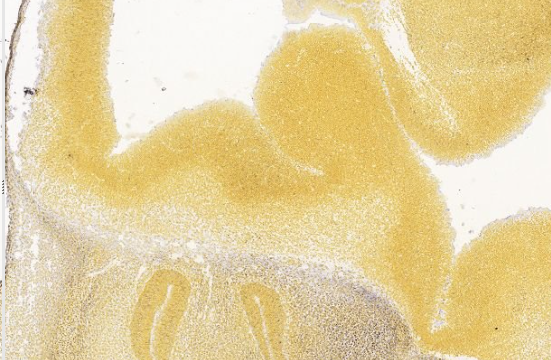 |
|  | CSPall | 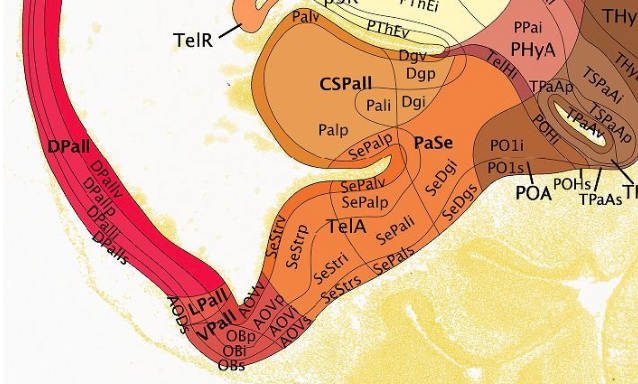 | 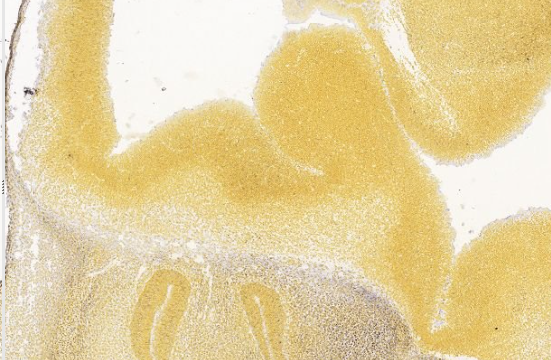 |
|  | VPall | 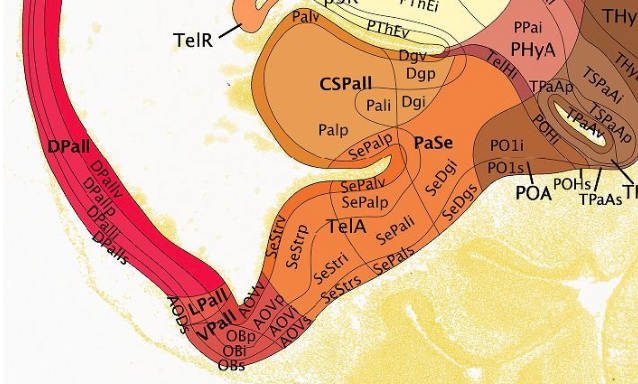 | 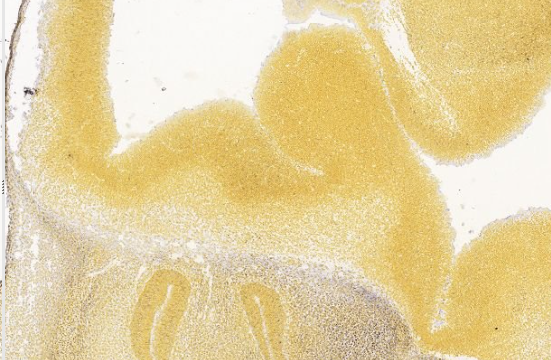 |
| E15.5 | Overview | 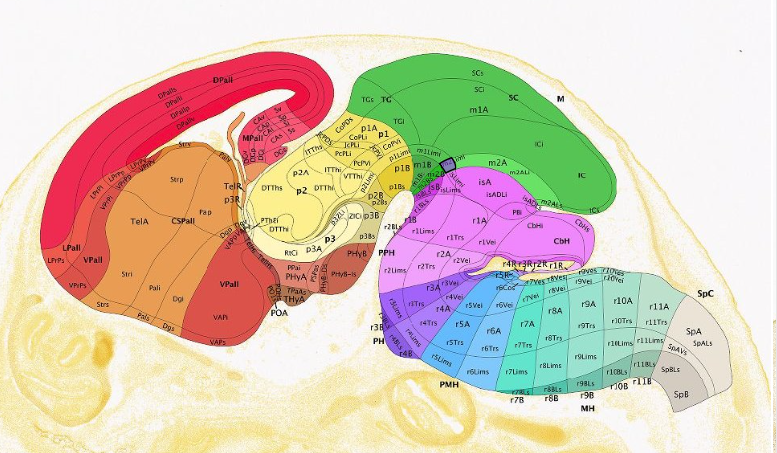 | 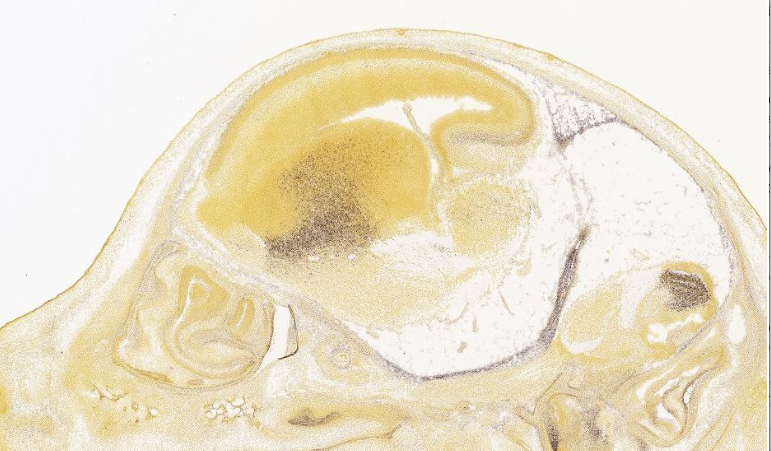 |
|  | POA | 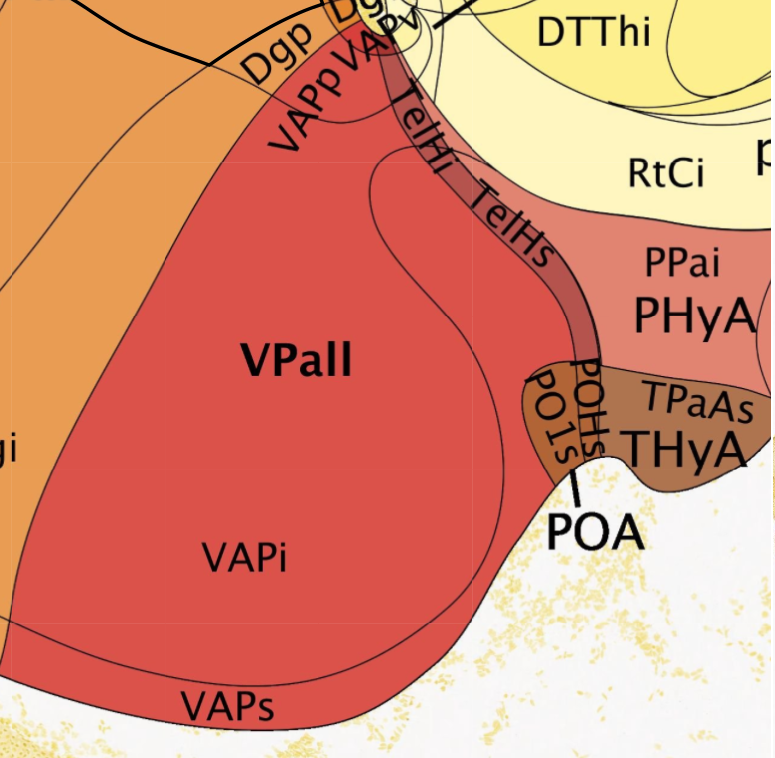 | 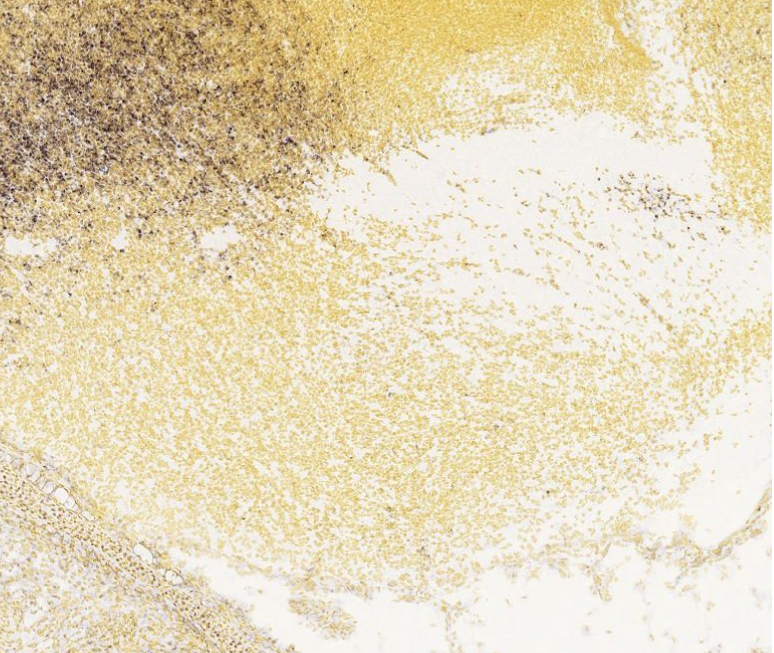 |
|  | TelA | 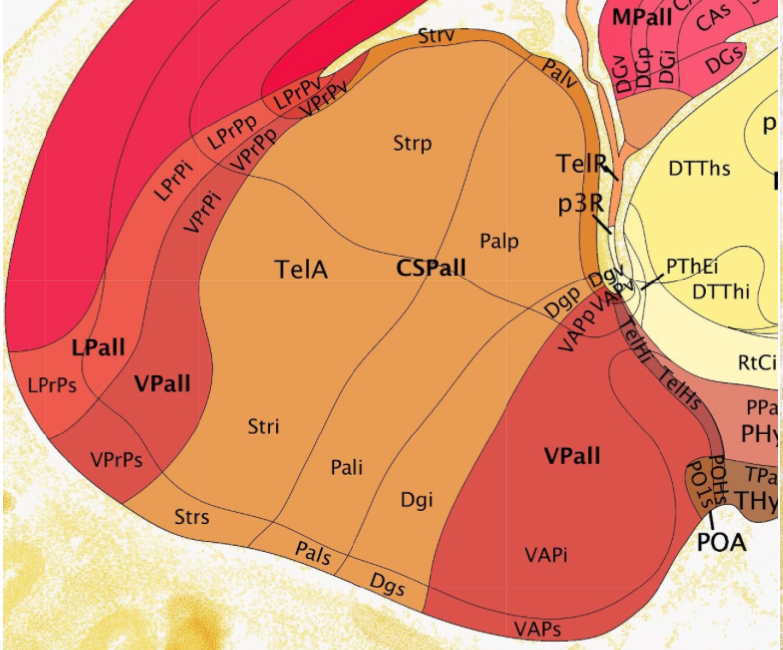 | 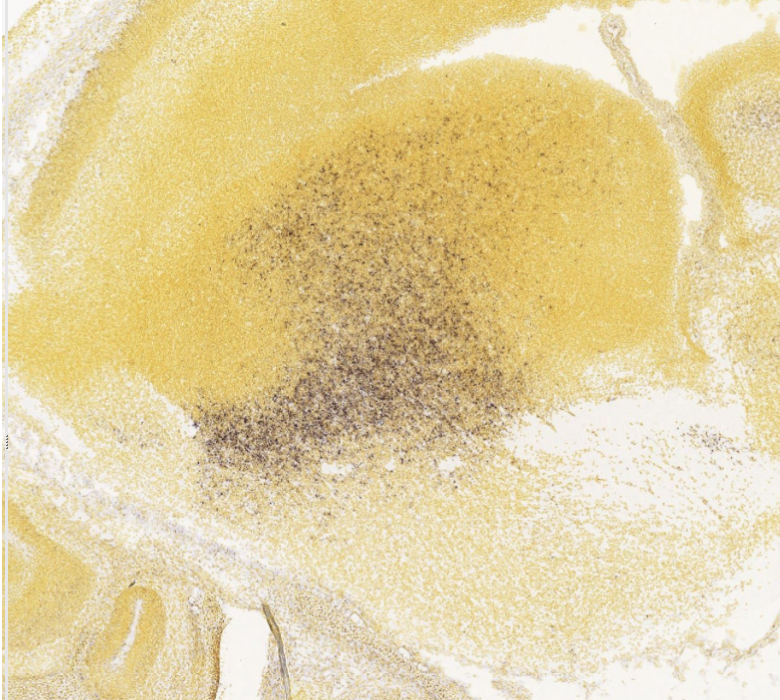 |
|  | LPall | 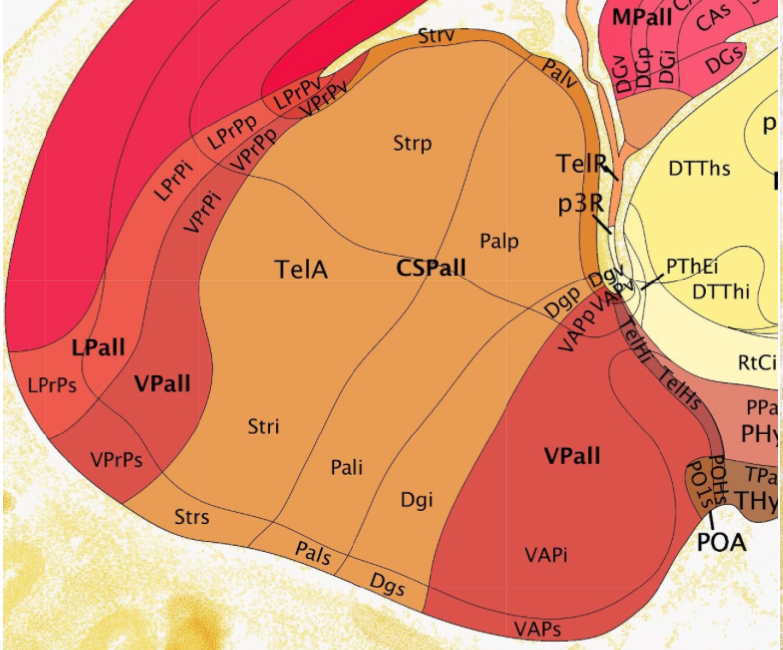 | 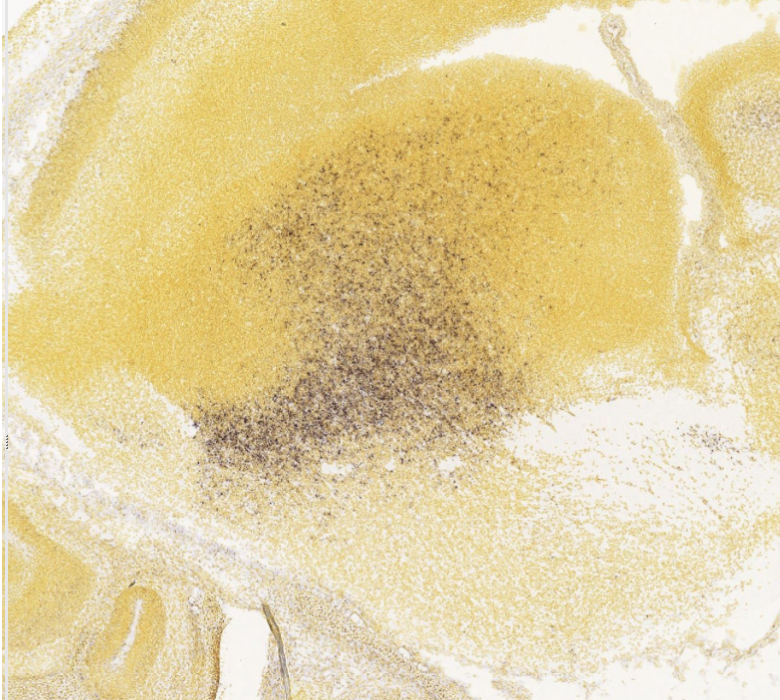 |
|  | CSPall | 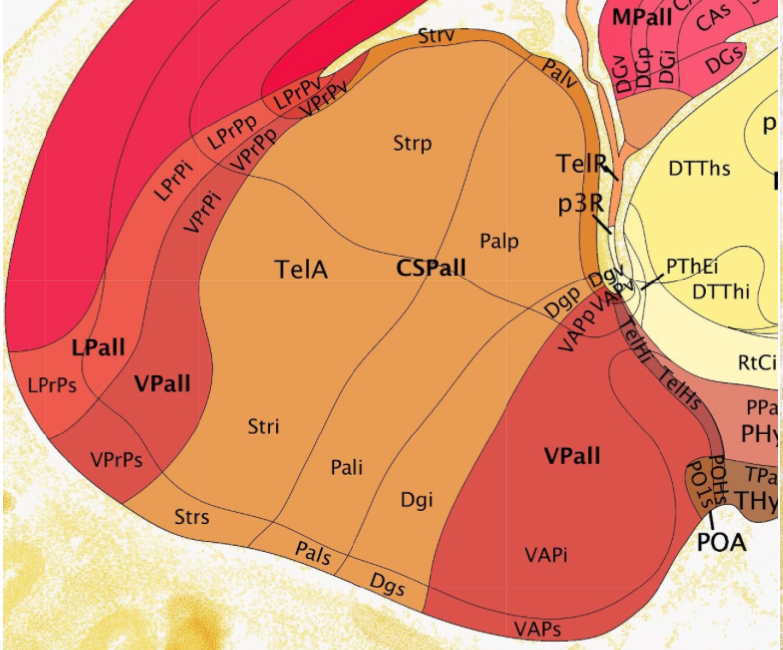 | 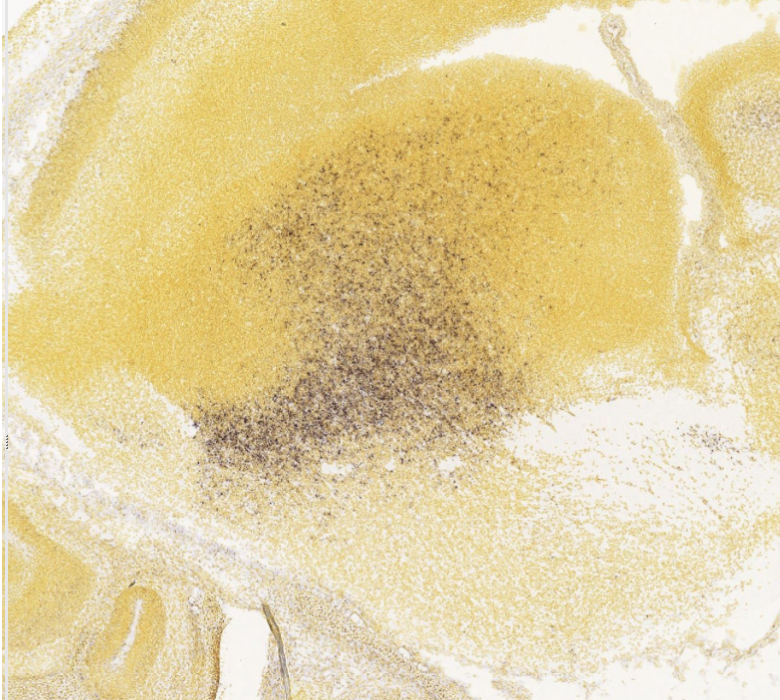 |
|  | VPall | 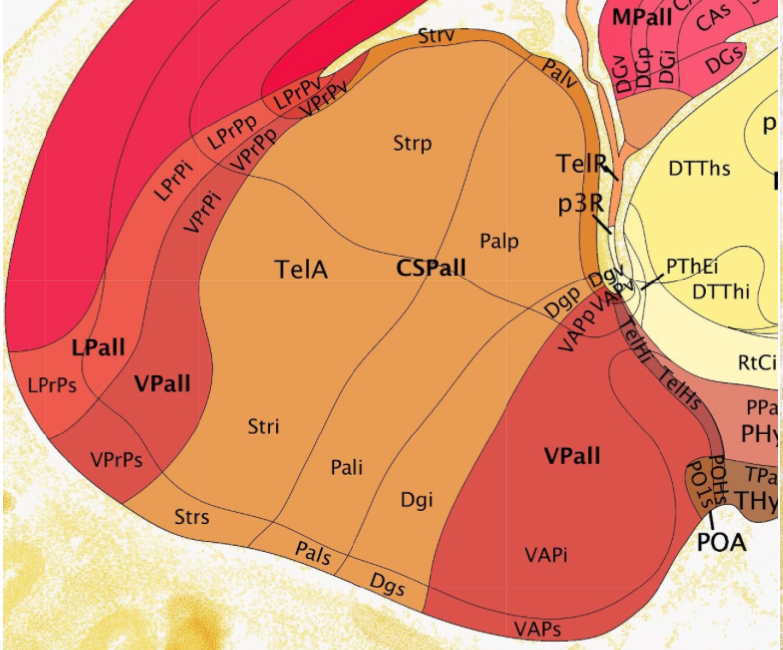 | 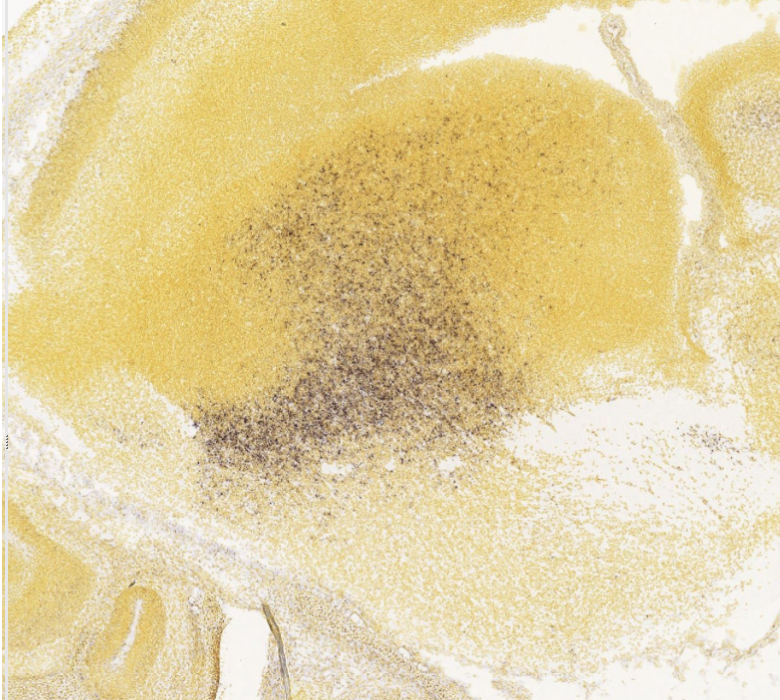 |
| E18.5 | Overview | 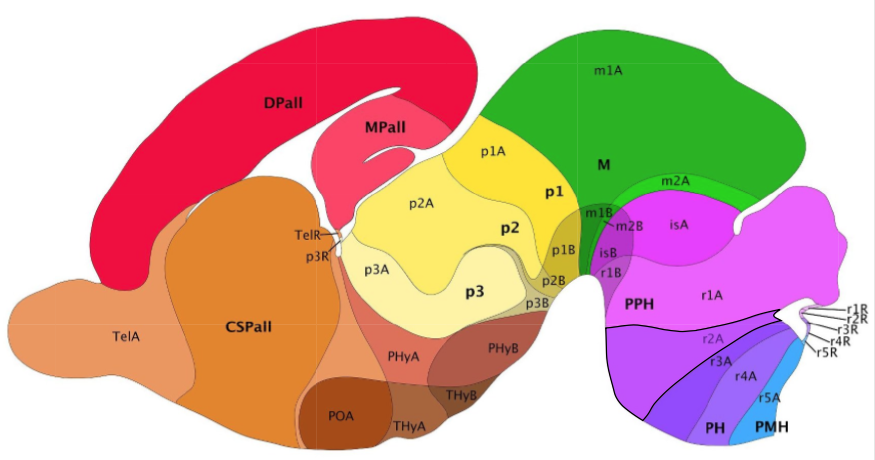 | 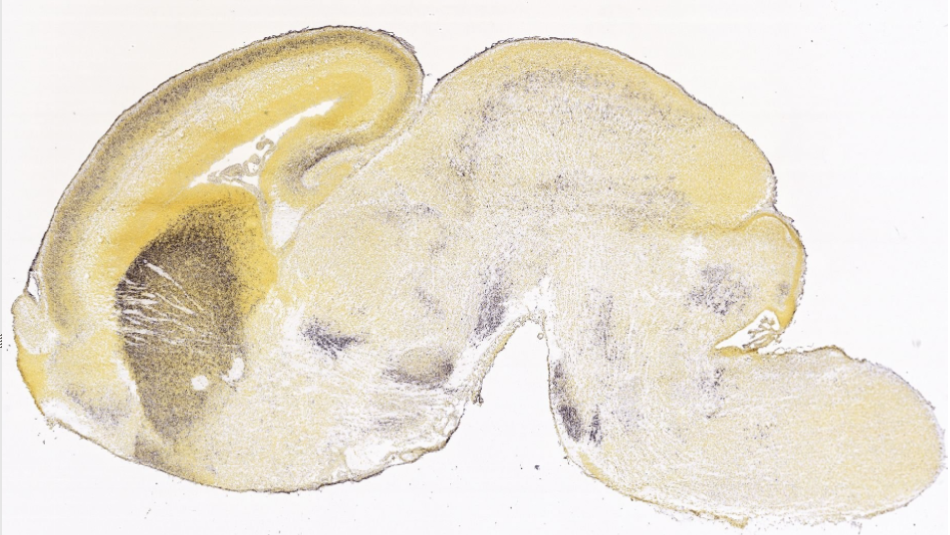 |
|  | POA | 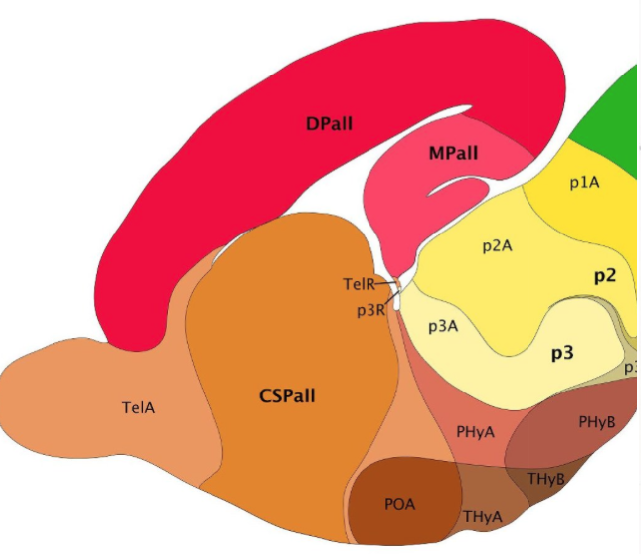 | 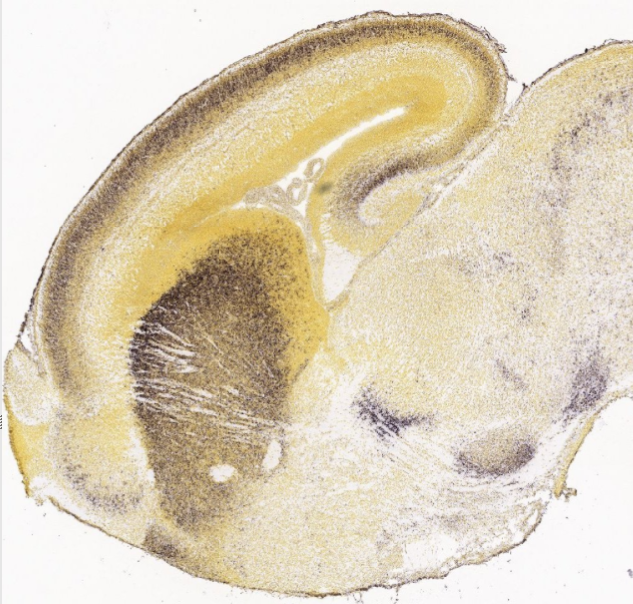 |
|  | TelA | 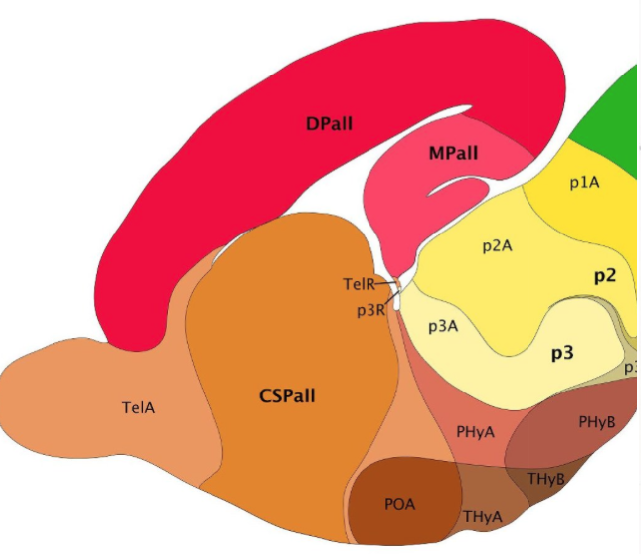 | 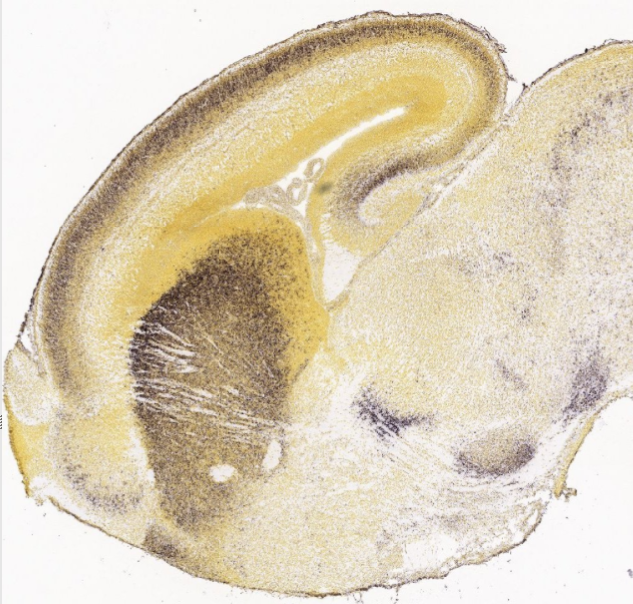 |
|  | CSPall | 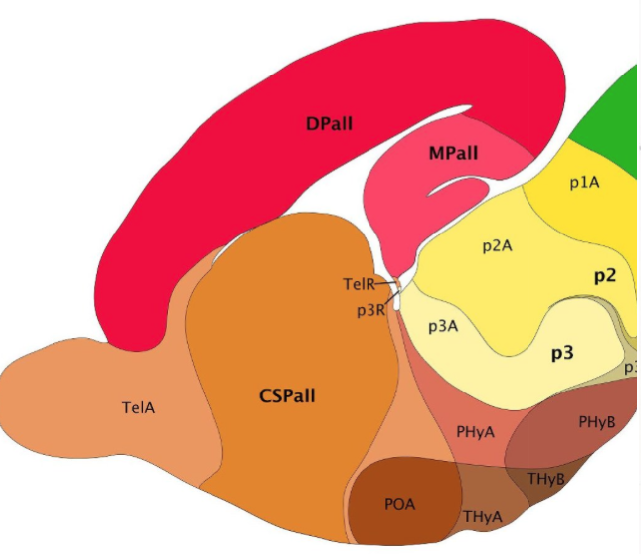 | 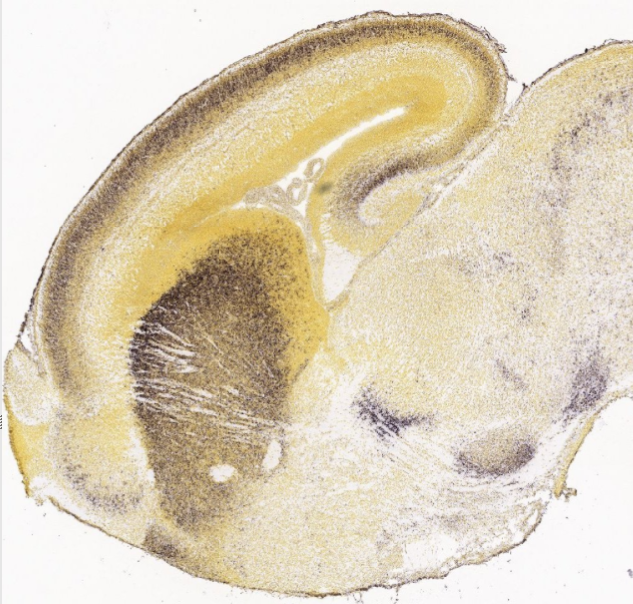 |
| P4 | Overview | 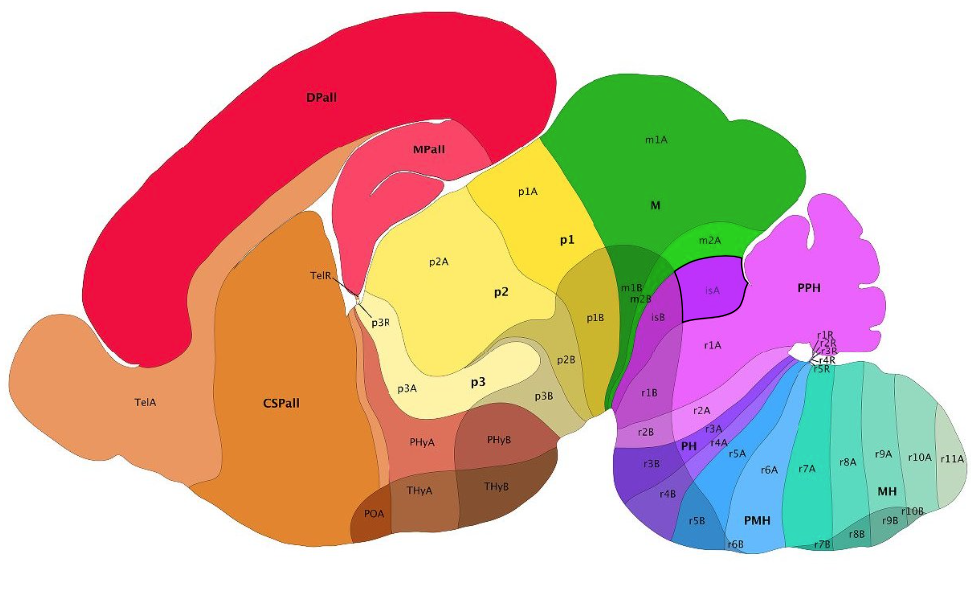 | 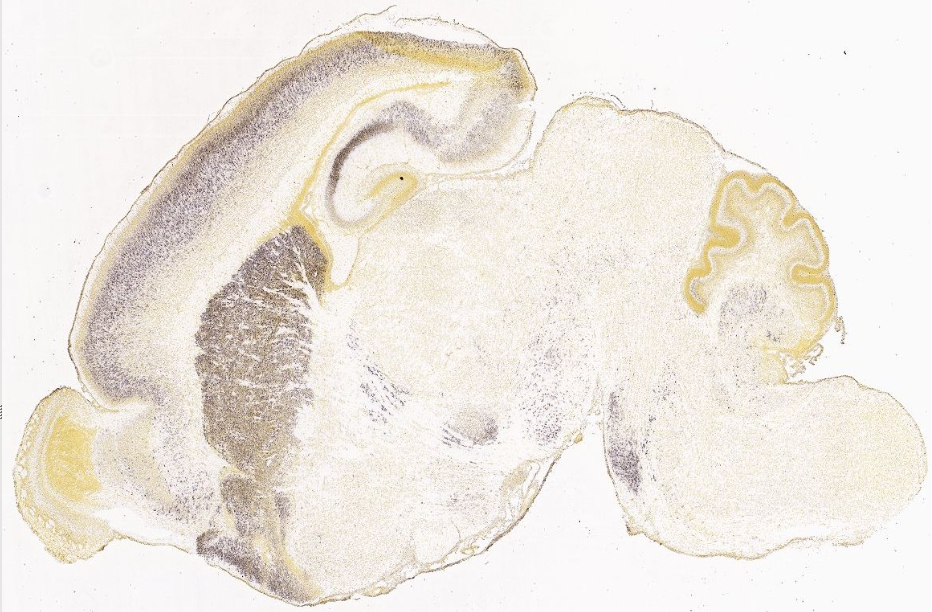 |
|  | POA | 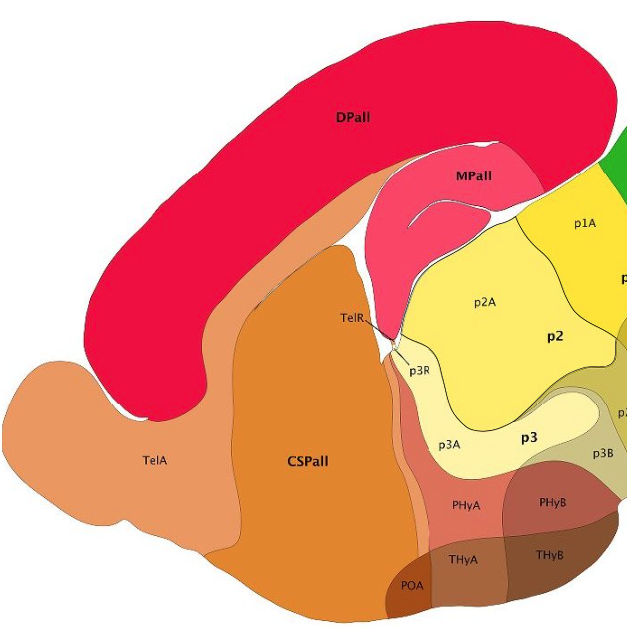 | 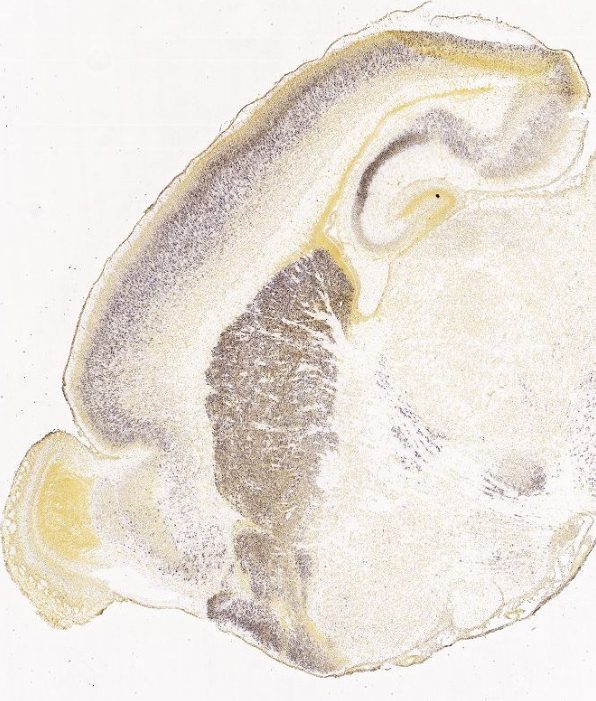 |
|  | TelA | 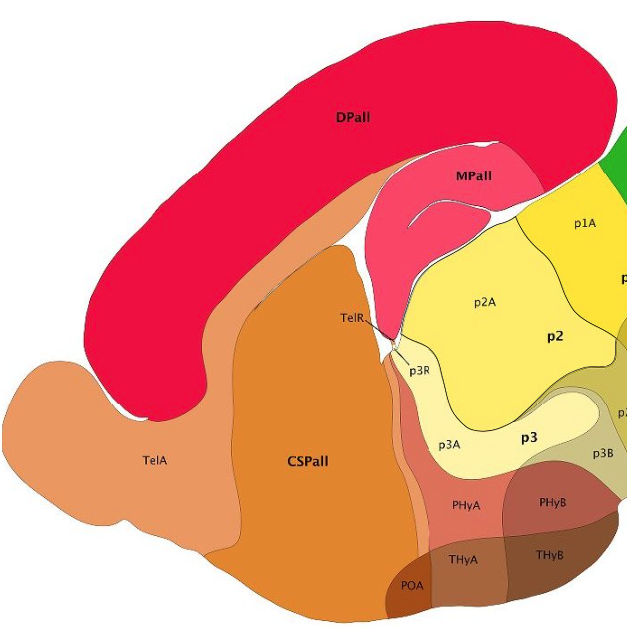 | 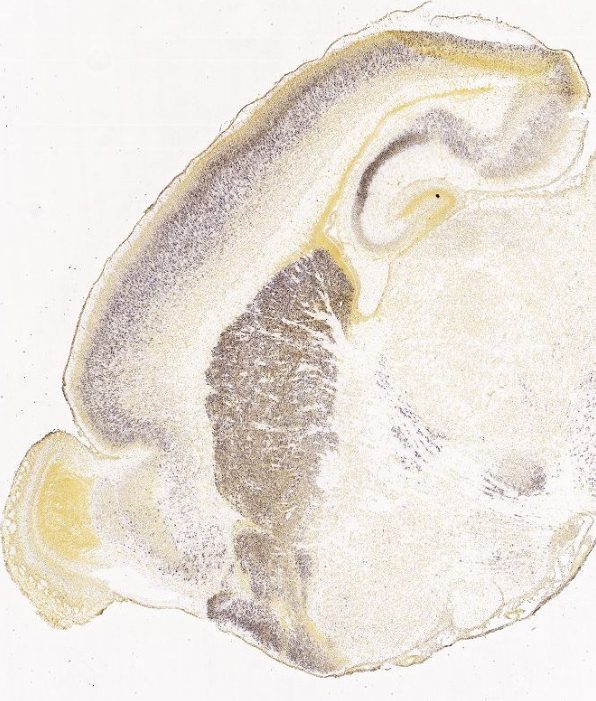 |
|  | CSPall | 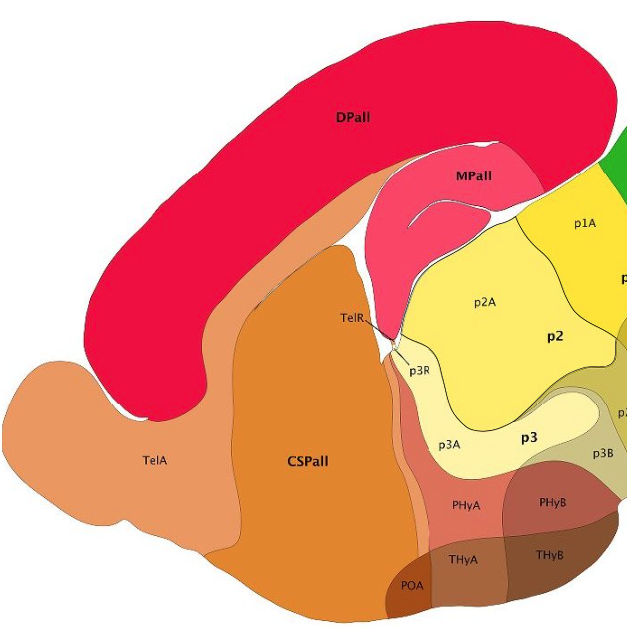 | 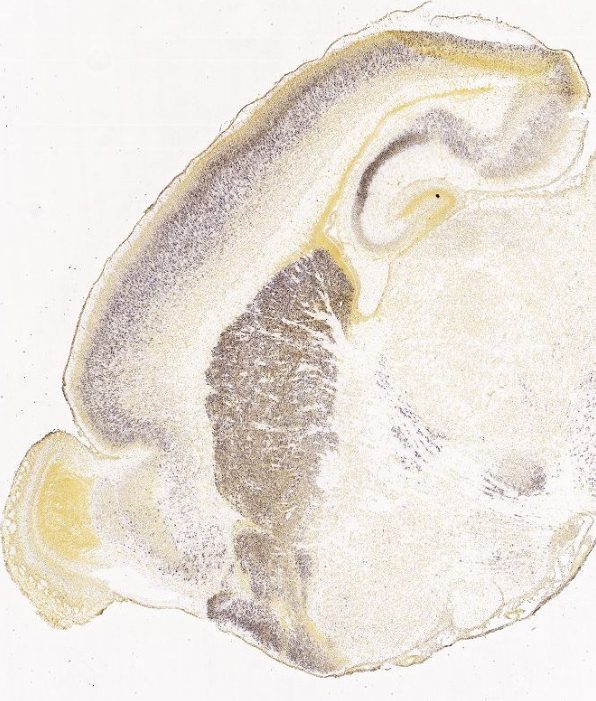 |
