# Supplementary material for: Identification of amygdala-expressed genes associated with autism spectrum disorder
Source: Mol Autism. 2020 May 27;11:39. doi: 10.1186/s13229-020-00346-1 (PMC7251751; doi:10.1186/s13229-020-00346-1)
Supplement: Supplementary file 3 — Additional file 3. Expression of 10 select genes. [file 13229_2020_346_MOESM3_ESM.docx]

| **Gene** | **ALLEN DEVELOPING MOUSE BRAIN DATA** | | | | |
| --- | --- | --- | --- | --- | --- |
| ALDH5A1 | HEAT MAP | | | [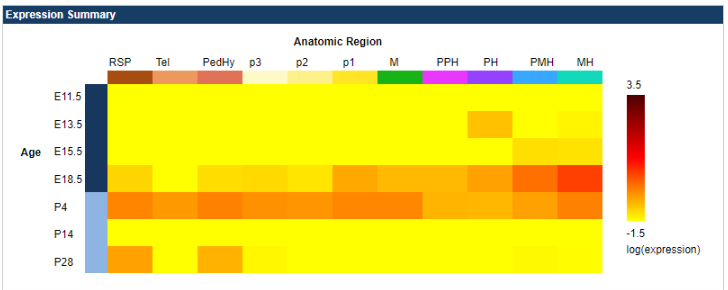](http://developingmouse.brain-map.org/gene/show/84449) | |
|  | IN SITUS | E11.5 | | No Expression | 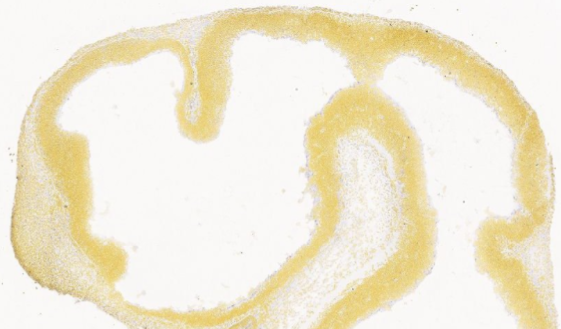 |
|  |  | E13.5 | | No Expression | 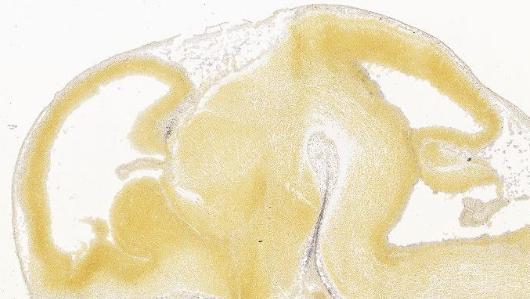 |
|  |  | E15.5 | | No Expression | 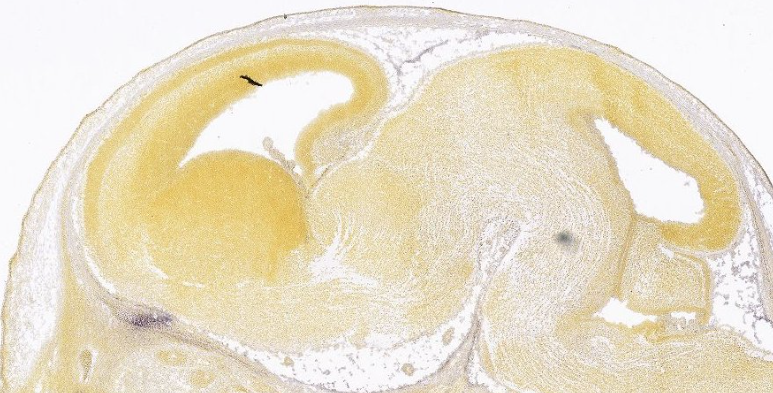 |
|  |  | E18.5 | | No Expression | 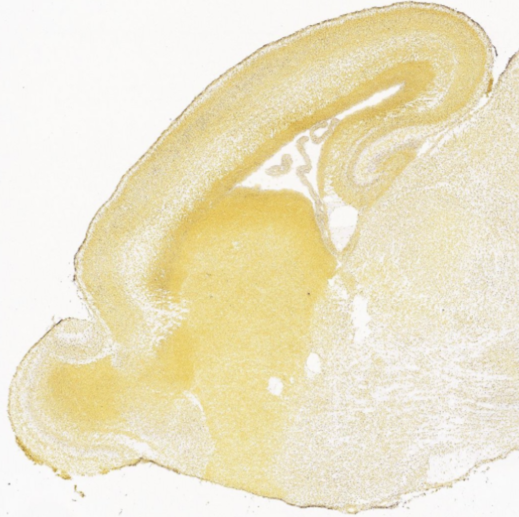 |
|  |  | P4 | | Low Expresion | 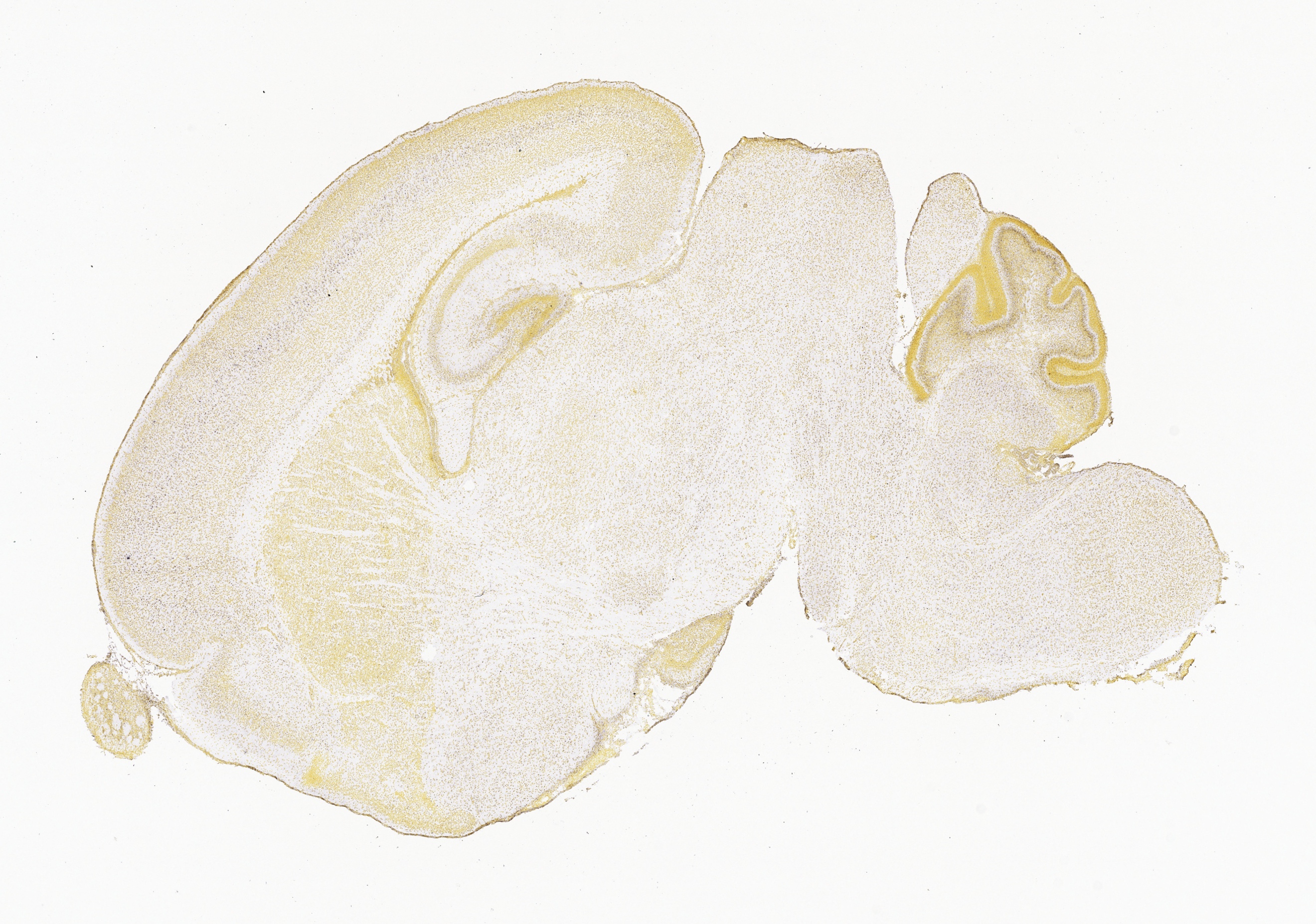 |
| KCNQ3 | HEAT MAP | | | [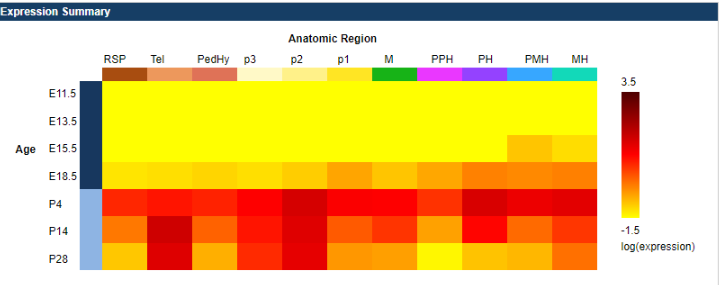](http://developingmouse.brain-map.org/gene/show/75016) | |
|  | IN SITUS | | E11.5 | No Expression | 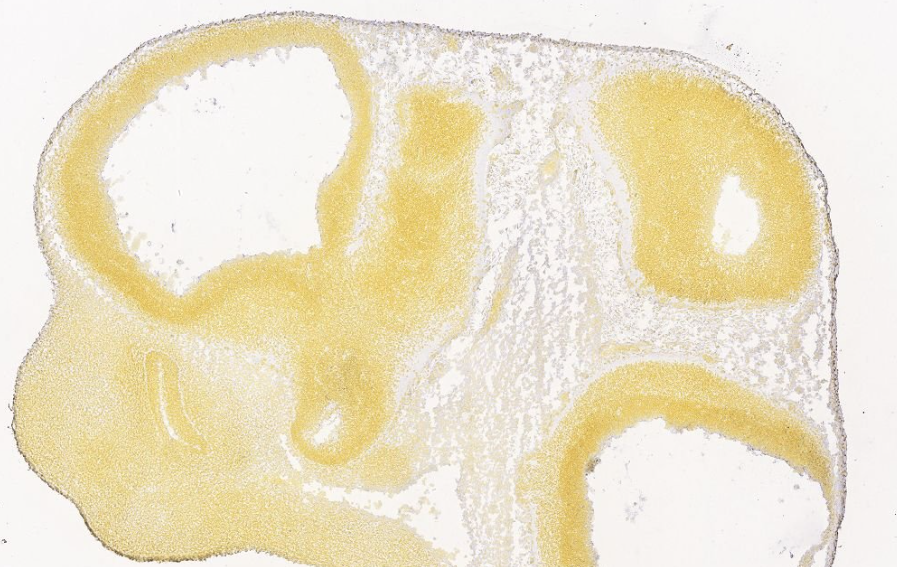 |
|  |  |  | E13.5 | No Expression | 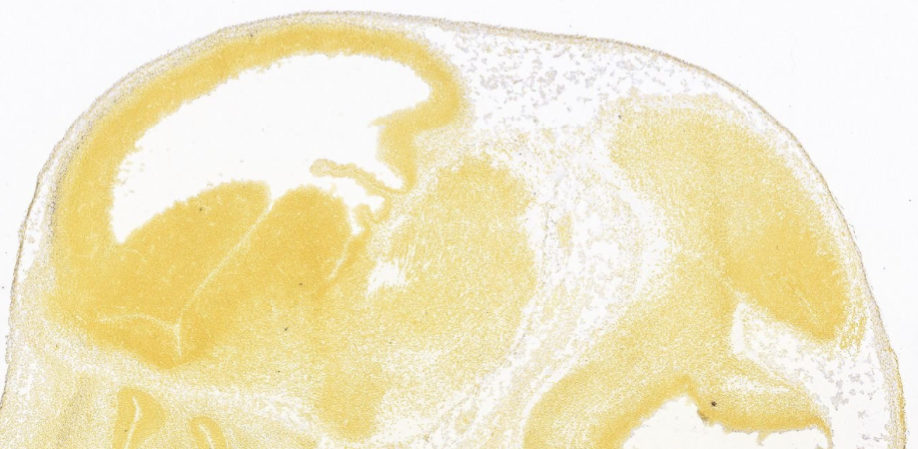 |
|  |  |  | E15.5 | No Expression | 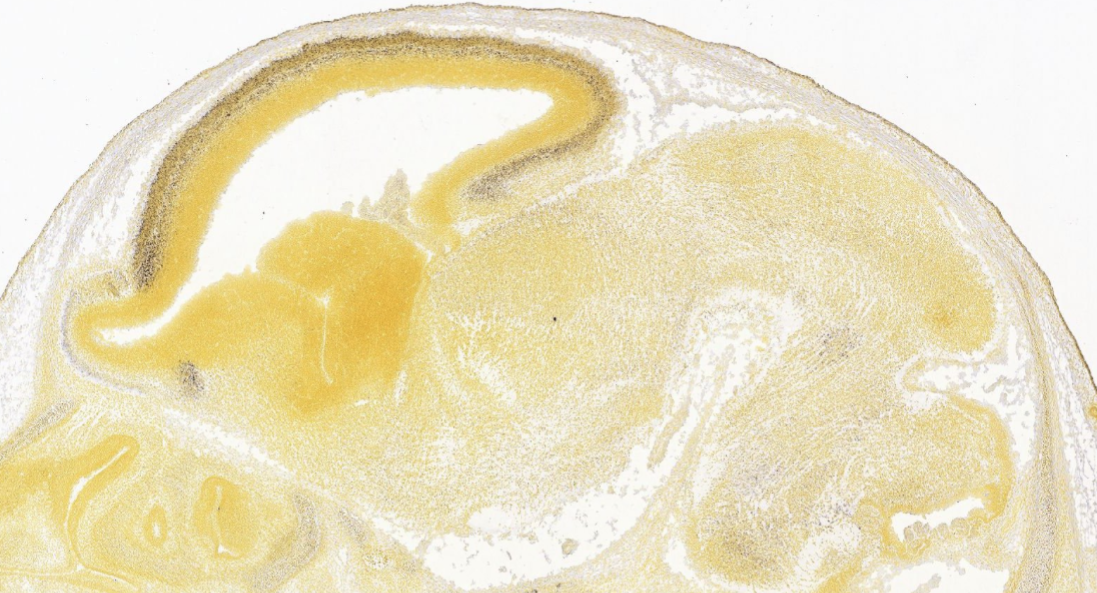 |
|  |  |  | E18.5 | No Expression | 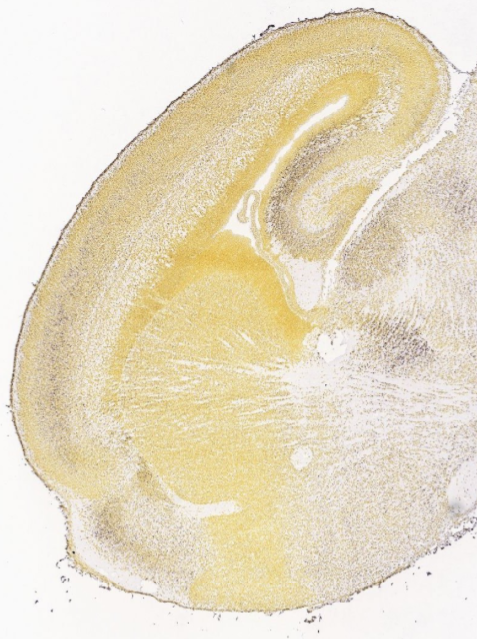 |
|  |  |  | P4 | High Expression | 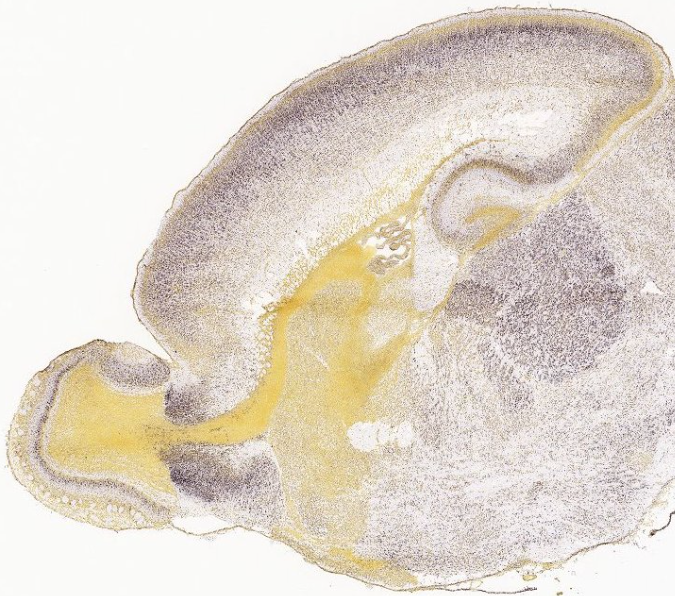 |
| GFAP | HEAT MAP | | | 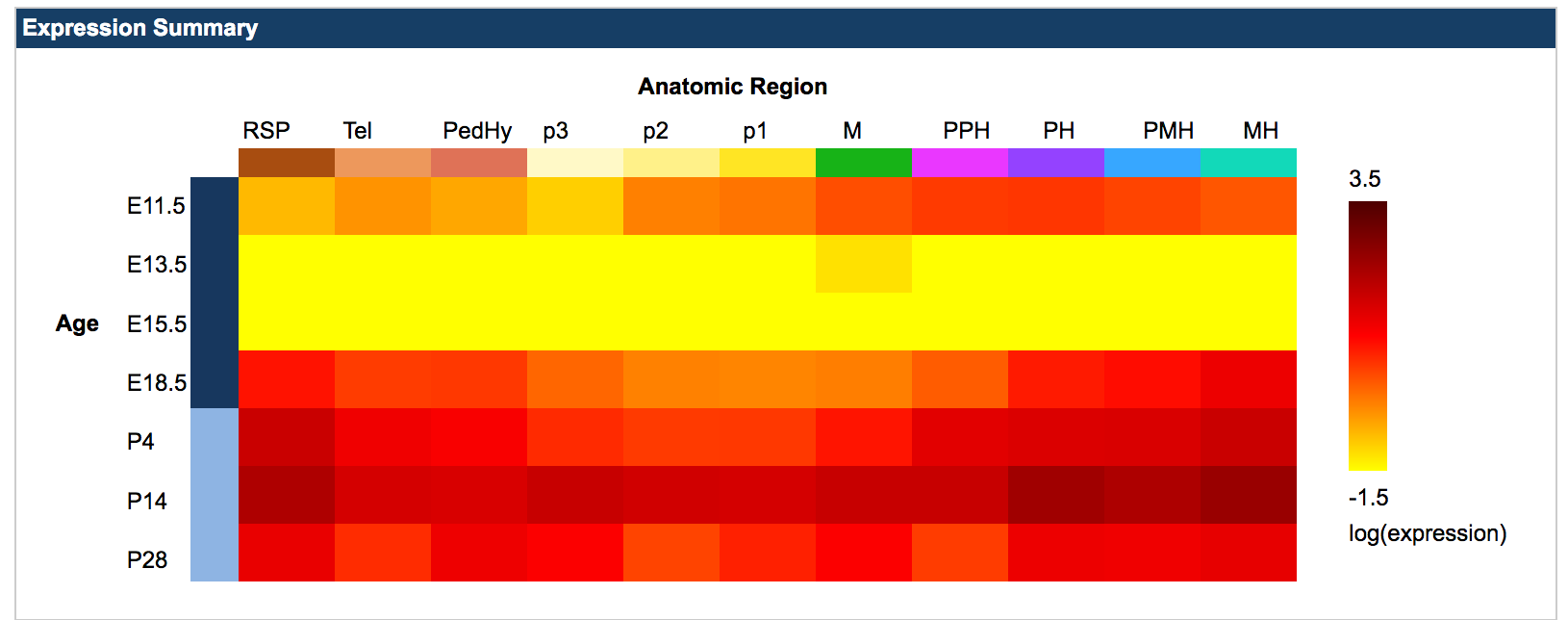 | |
|  | IN SITUS | | E11.5 | Low Expression | 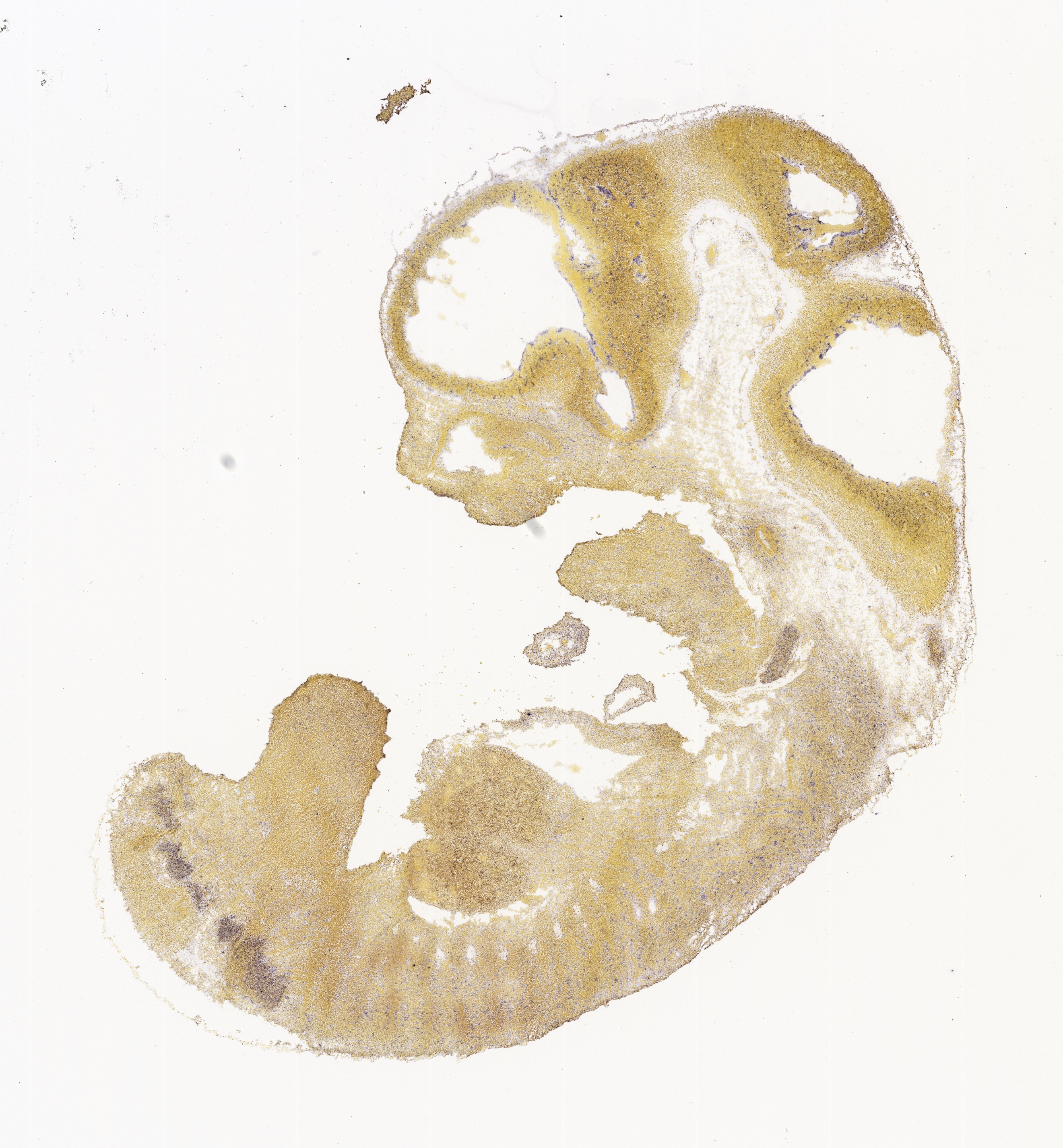 |
|  |  |  | E13.5 | No  Expression | 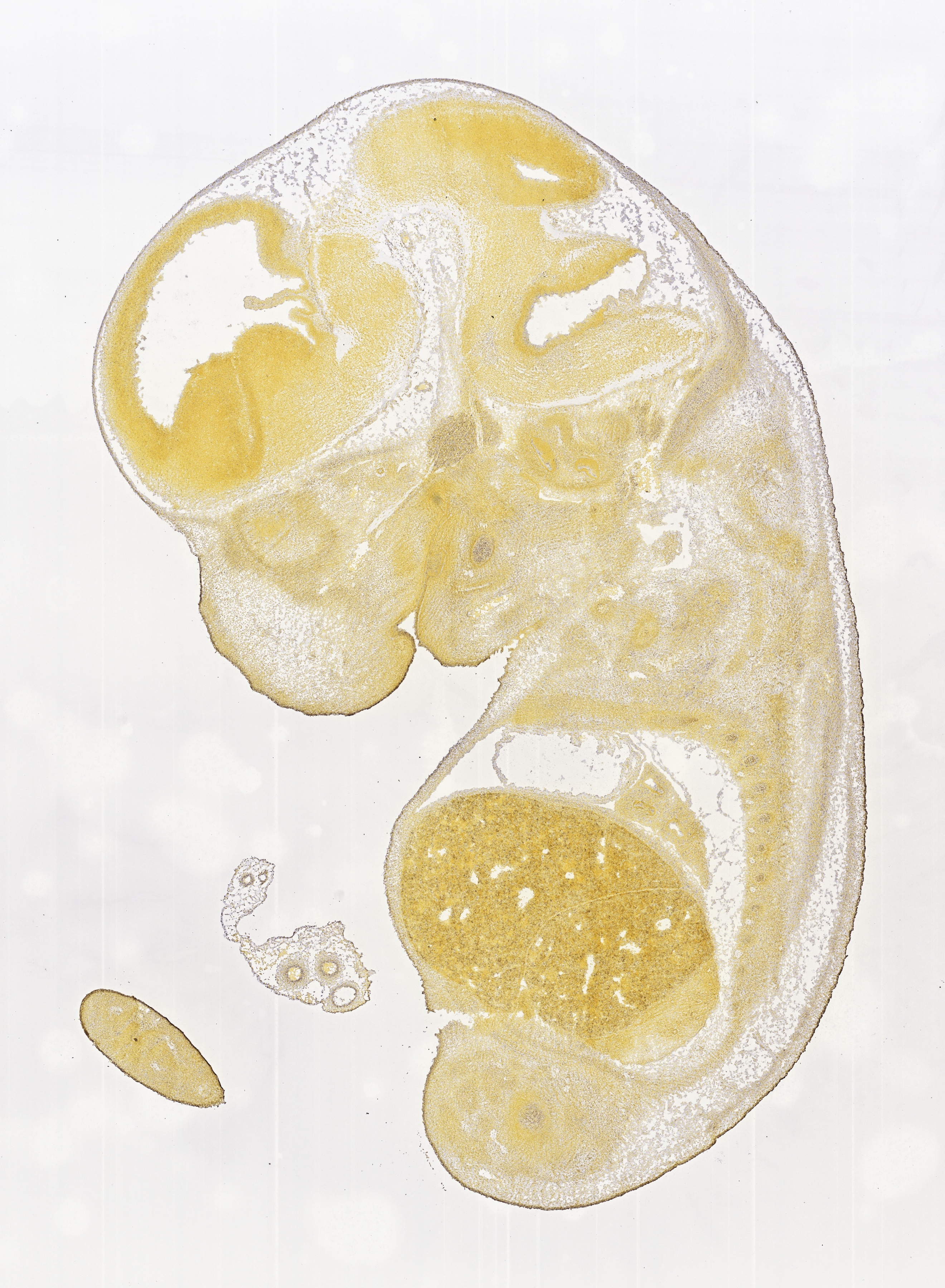 |
|  |  |  | E15.5 | No  Expression | 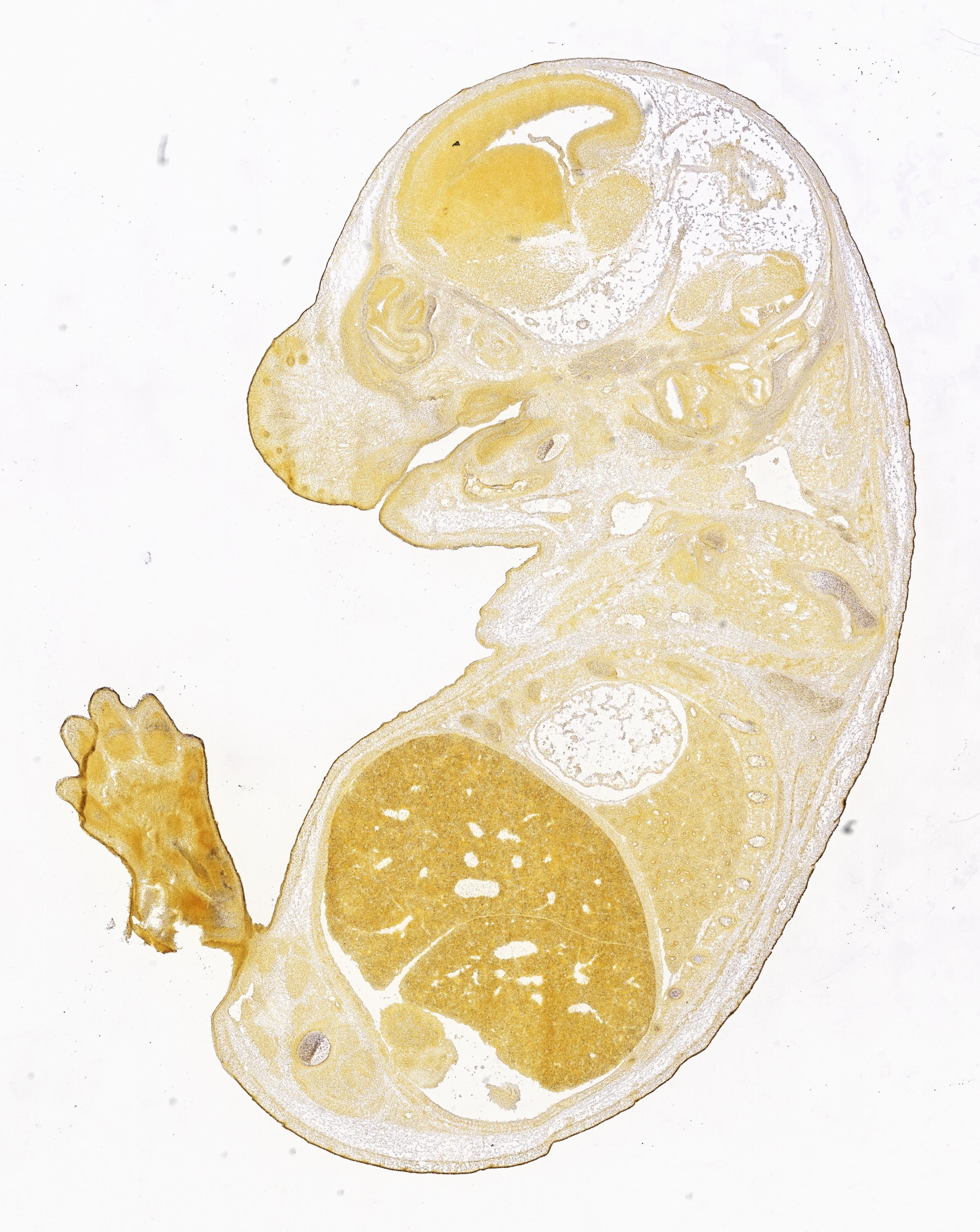 |
|  |  |  | E18.5 | High Expression | 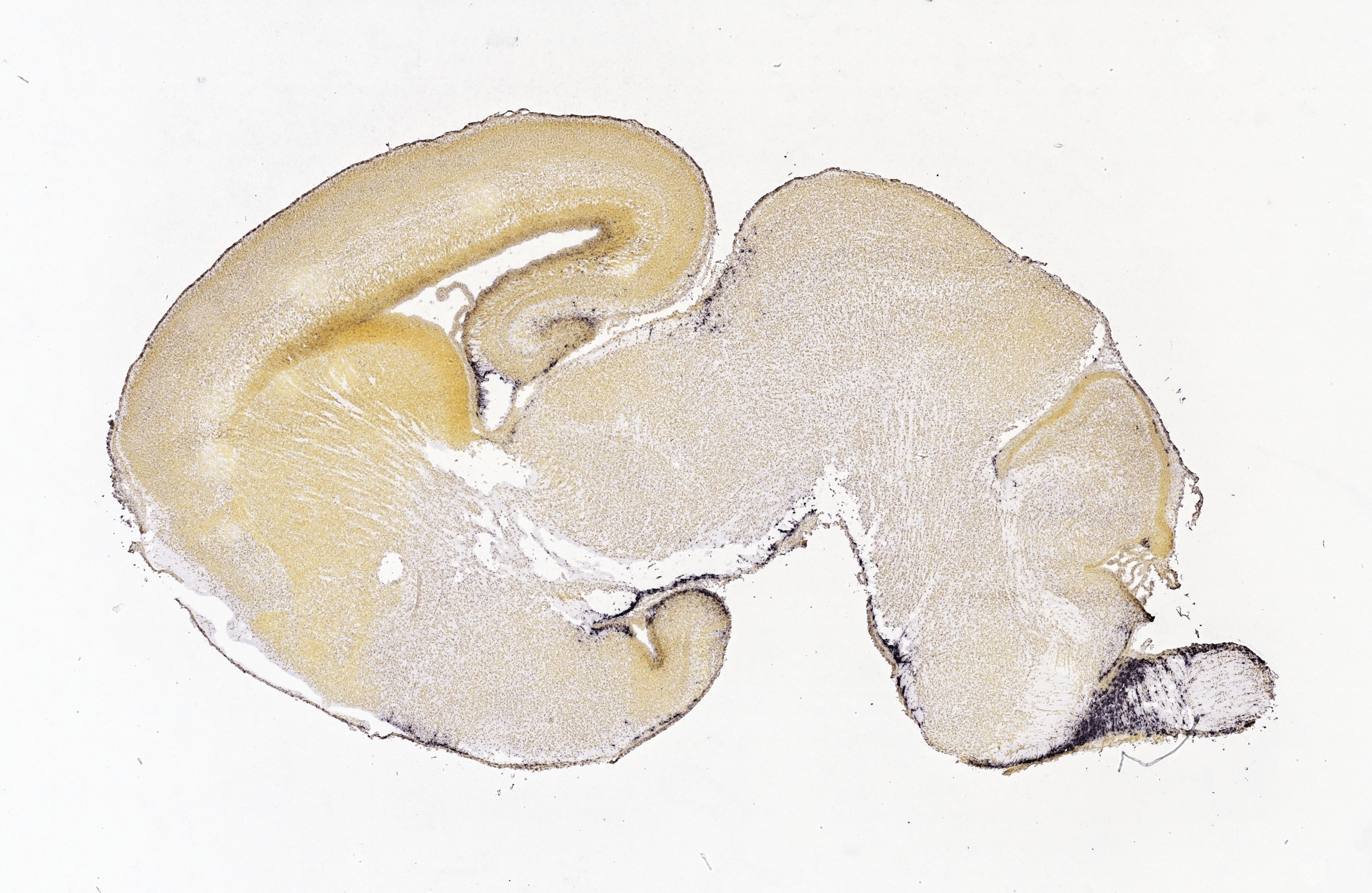 |
|  |  |  | P4 | High  Expression | 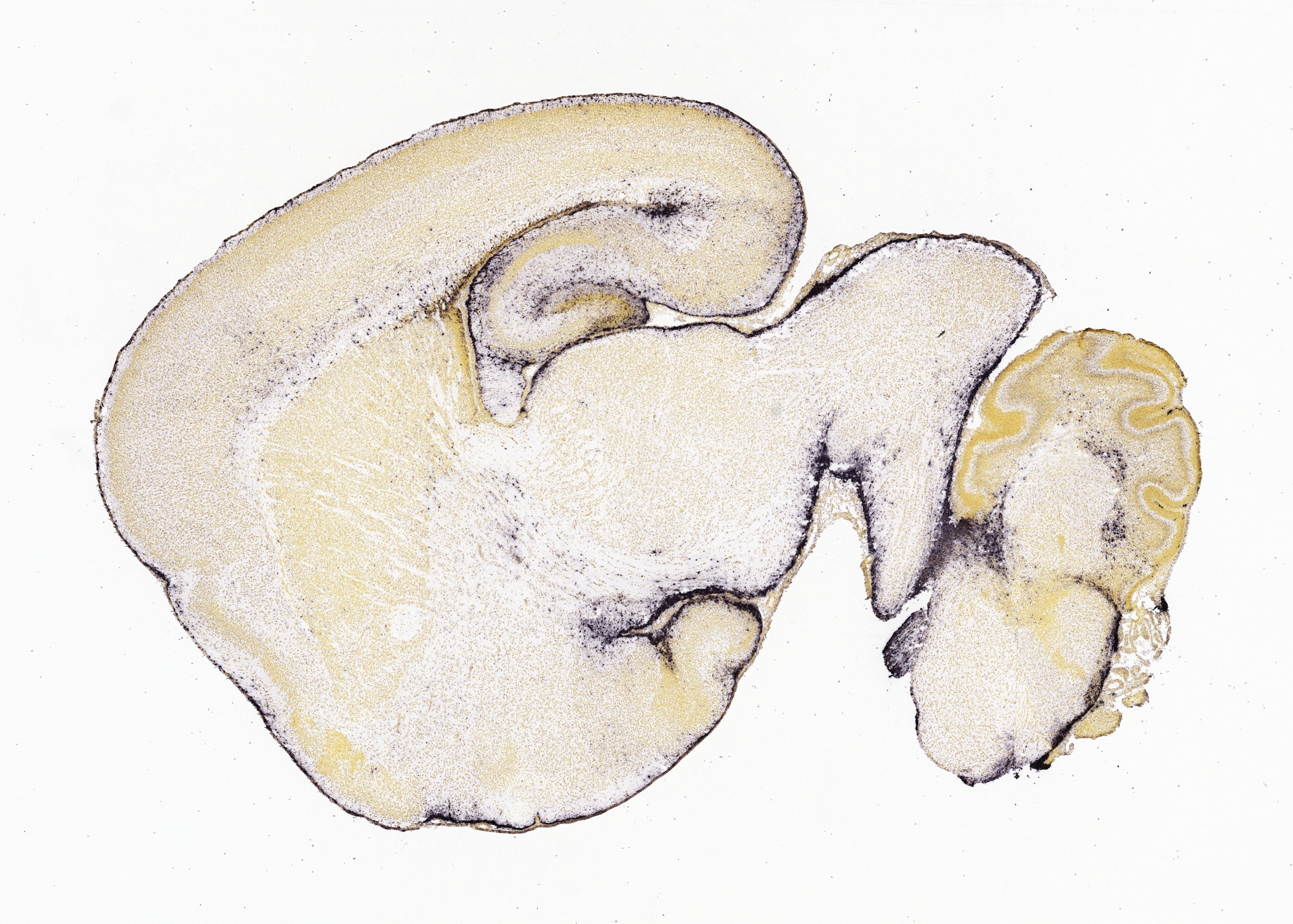 |
| RELN | HEAT MAP | | | [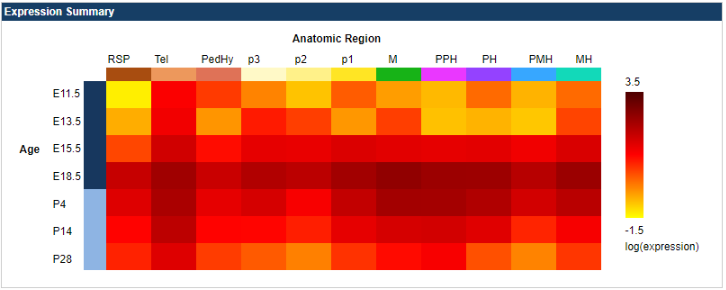](http://developingmouse.brain-map.org/gene/show/19462) | |
|  | IN SITUS | | E11.5 | Low  Expression | 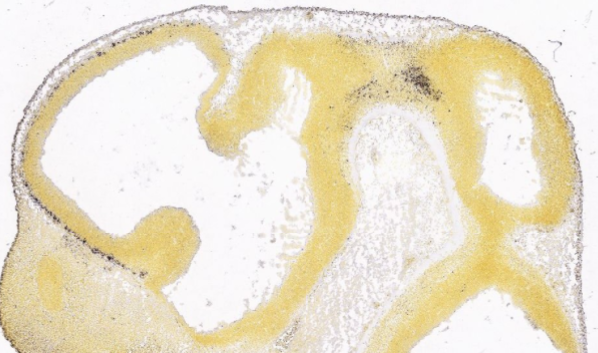 |
|  |  |  | E13.5 | High  Expression | 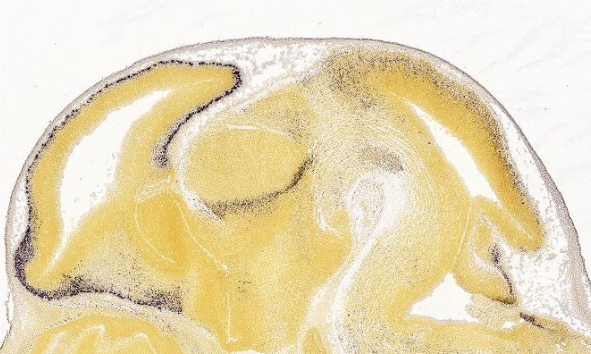 |
|  |  |  | E15.5 | High  Expression | 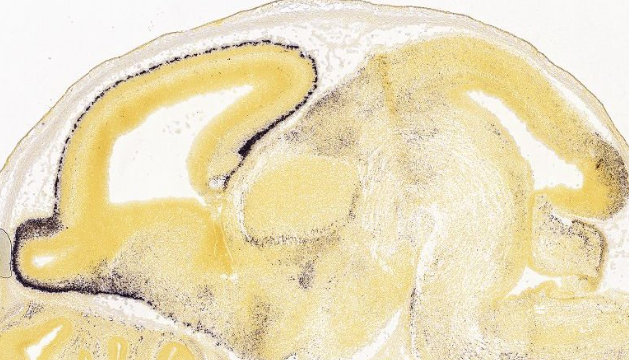 |
|  |  |  | E18.5 | High Expression | 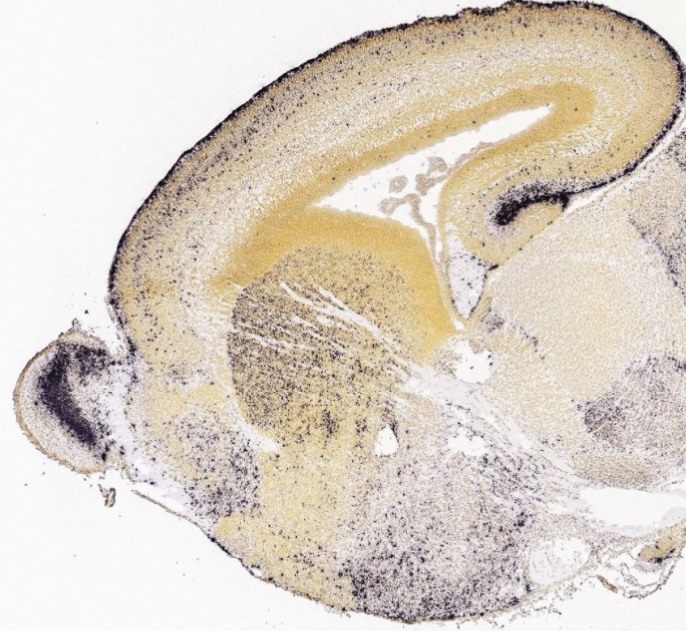 |
|  |  |  | P4 | High  Expression | 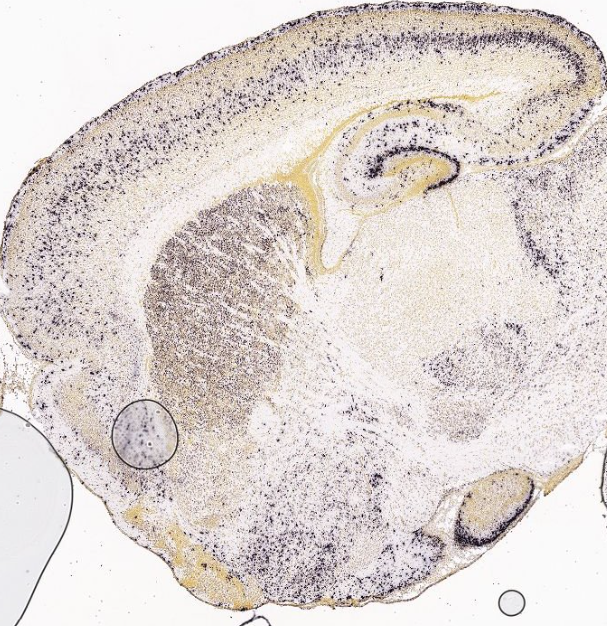 |
| NRXN1 | HEAT MAP | | | [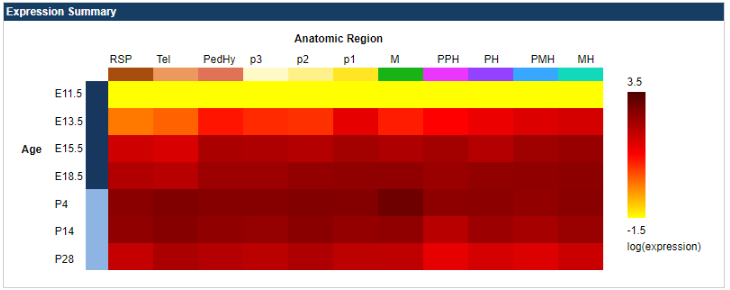](http://developingmouse.brain-map.org/gene/show/17956) | |
|  | IN SITUS | | E11.5 | No Expression | 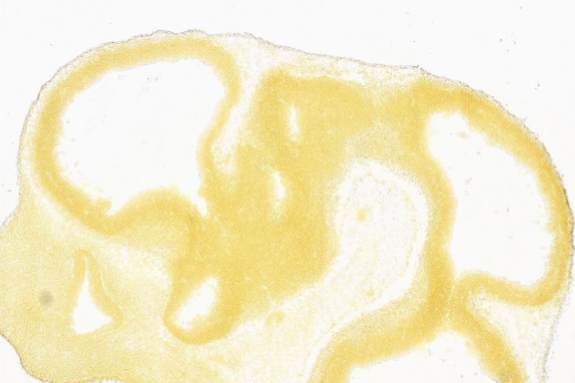 |
|  |  |  | E13.5 | LowExpression | 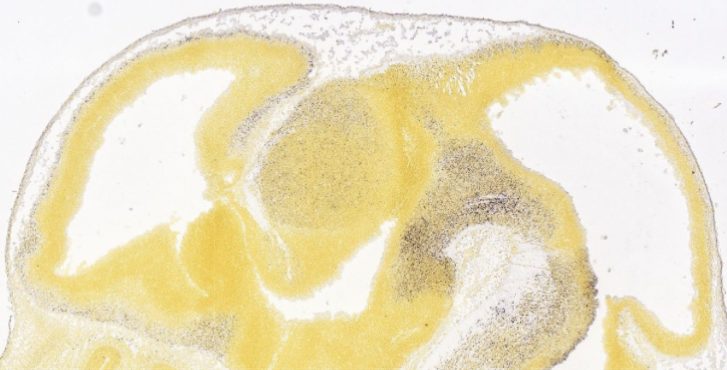 |
|  |  |  | E15.5 | High  Expression | 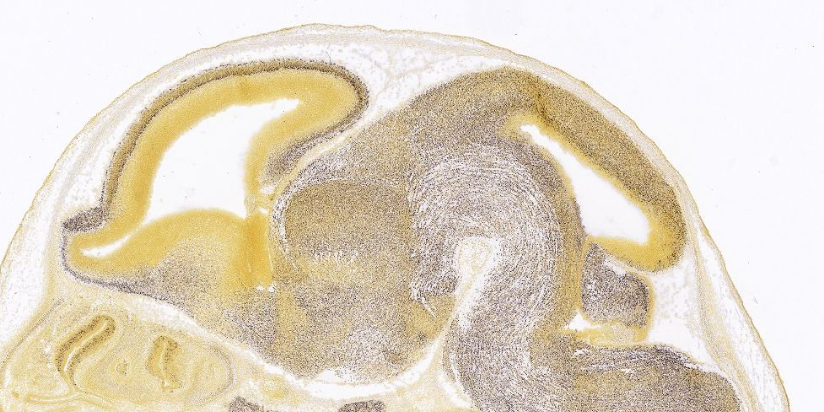 |
|  |  |  | E18.5 | High Expression | 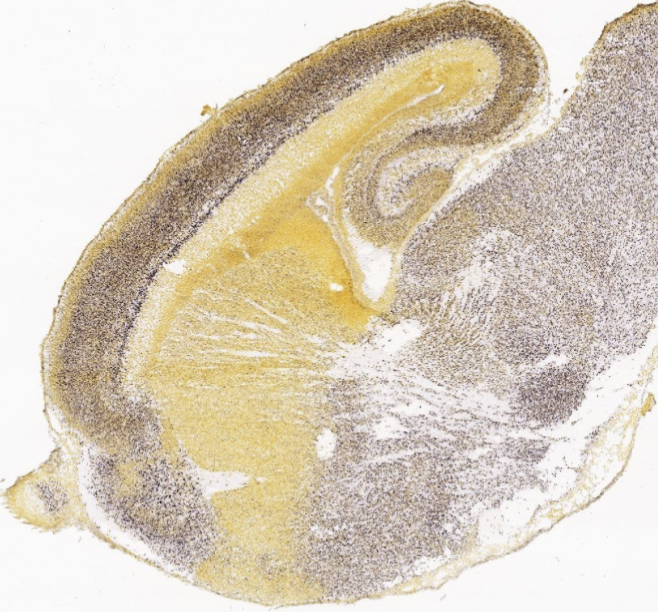 |
|  |  |  | P4 | High  Expression | 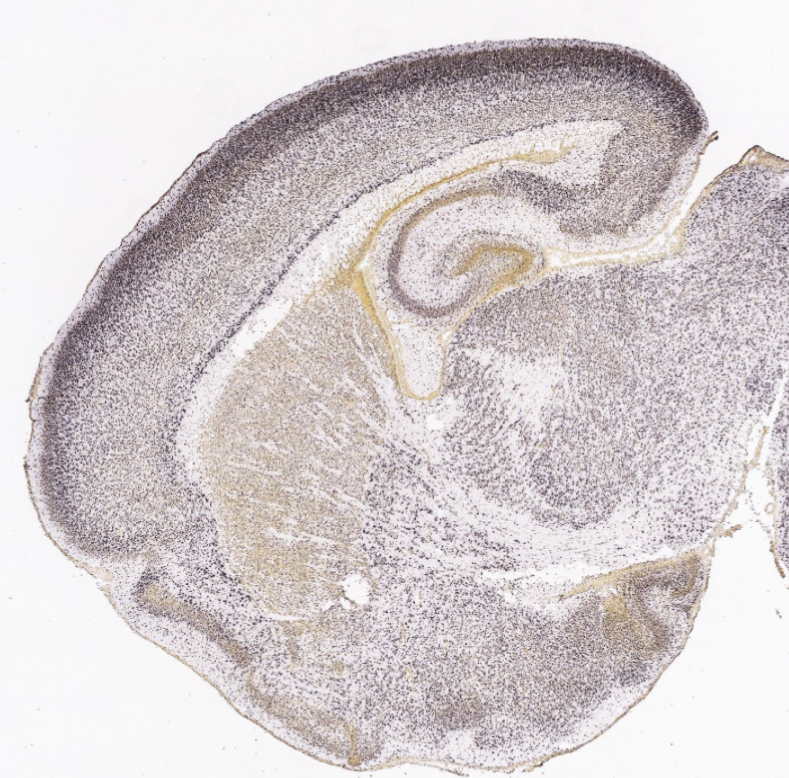 |
| GRIN2B | HEAT MAP | | | [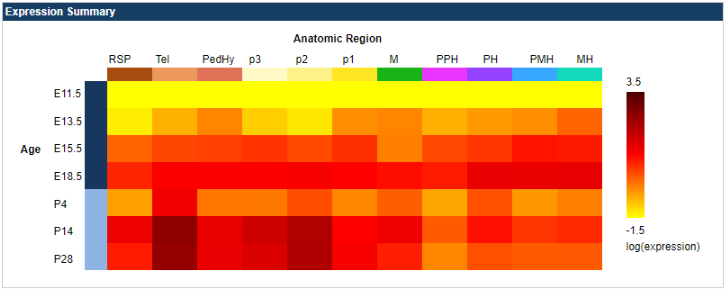](http://developingmouse.brain-map.org/gene/show/14588) | |
|  | IN SITUS | | E11.5 | No Expression | 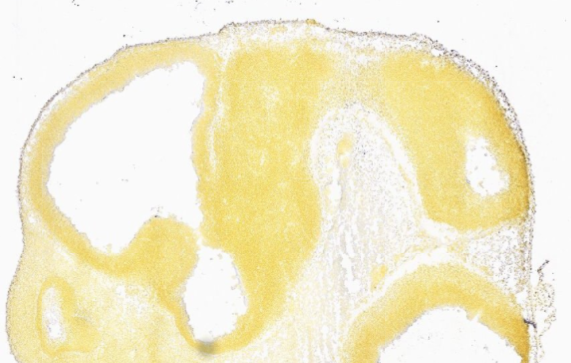 |
|  |  |  | E13.5 | Low  Expression | 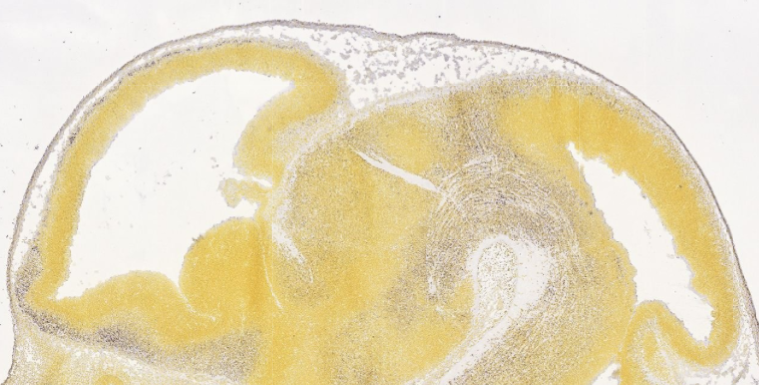 |
|  |  |  | E15.5 | Low  Expression | 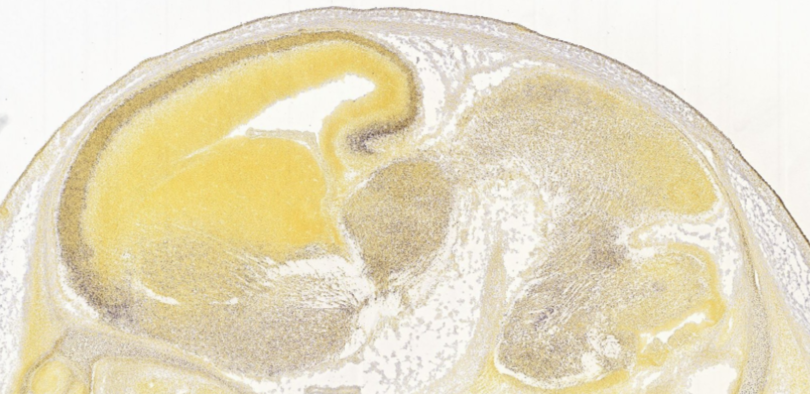 |
|  |  |  | E18.5 | High  Expression | 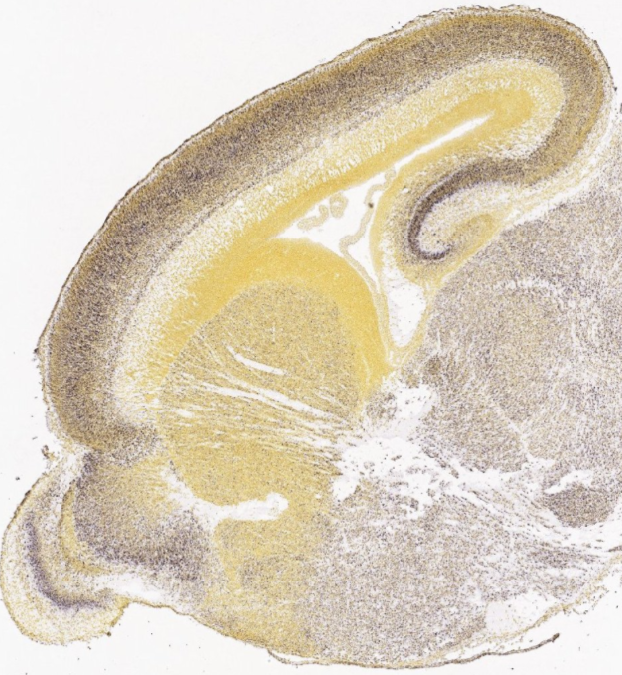 |
|  |  |  | P4 | High  Expression | 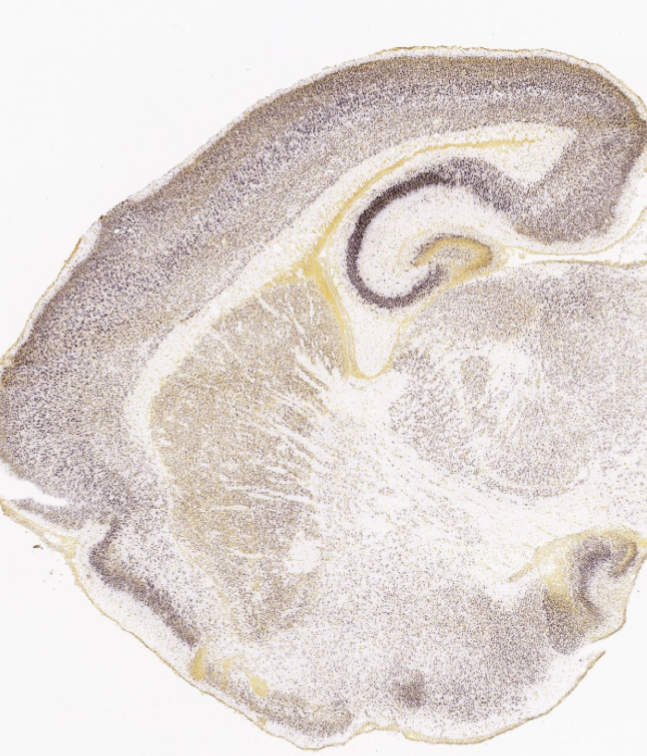 |
| GRIN1 | HEAT MAP | | | 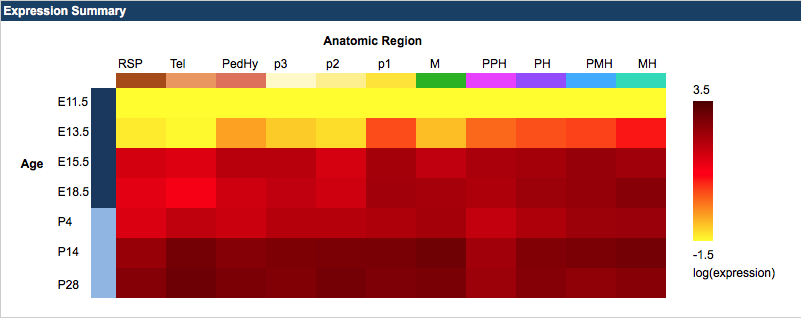 | |
|  | IN SITUS | | E11.5 | No Expression | 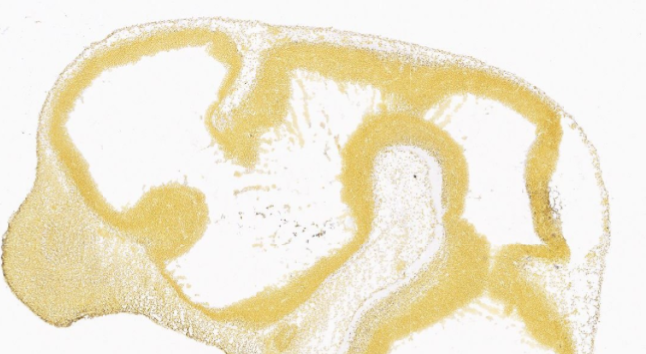 |
|  |  |  | E13.5 | Low  Expression | 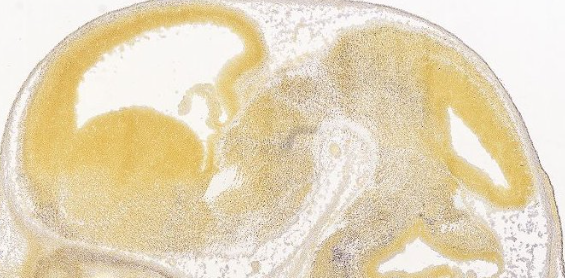 |
|  |  |  | E15.5 | High Expression | 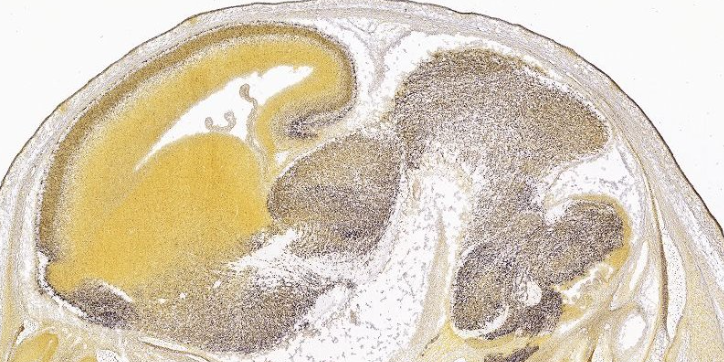 |
|  |  |  | E18.5 | High Expression | 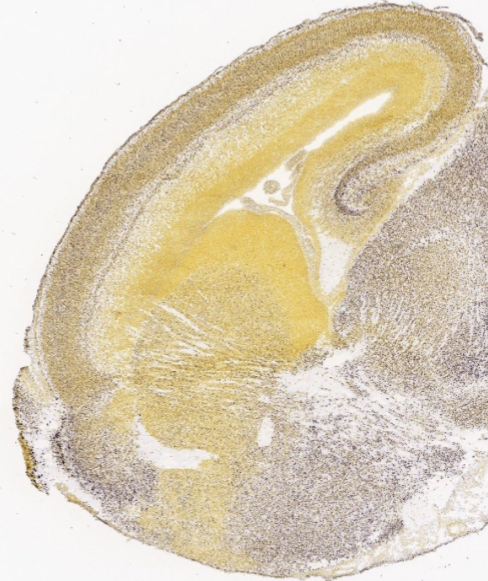 |
|  |  |  | P4 | High Expression | 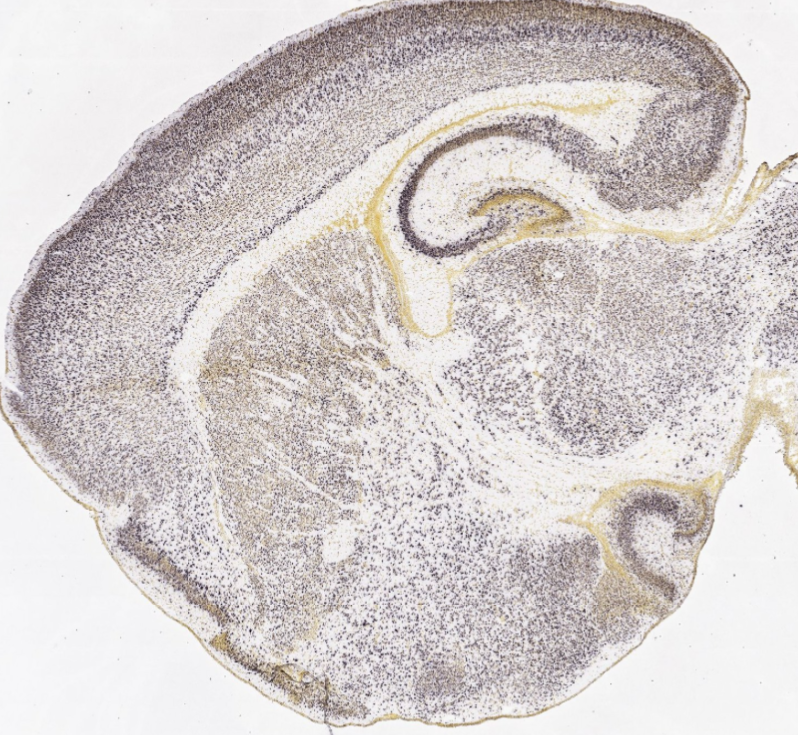 |
| ACHE | HEAT MAP | | | [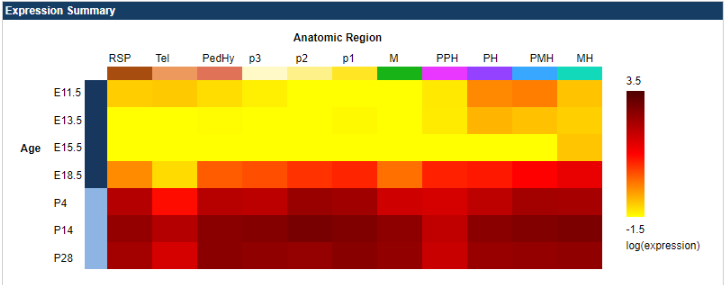](http://developingmouse.brain-map.org/gene/show/11212) | |
|  | IN SITUS | | E11.5 | No Expression | 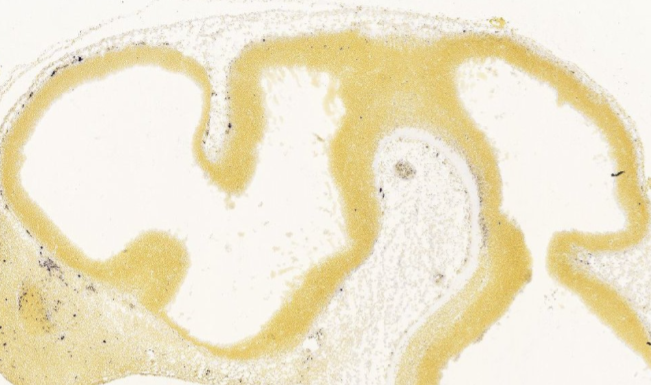 |
|  |  |  | E13.5 | Low  Expression | 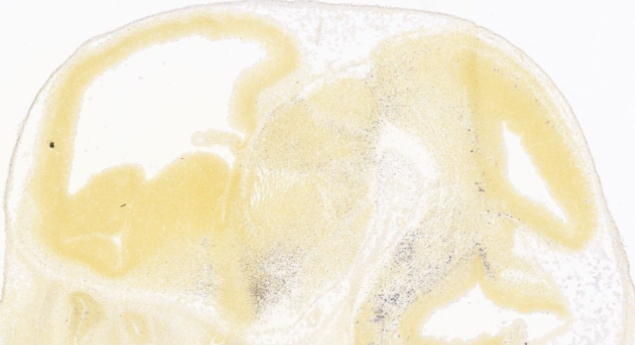 |
|  |  |  | E15.5 | No Expression | 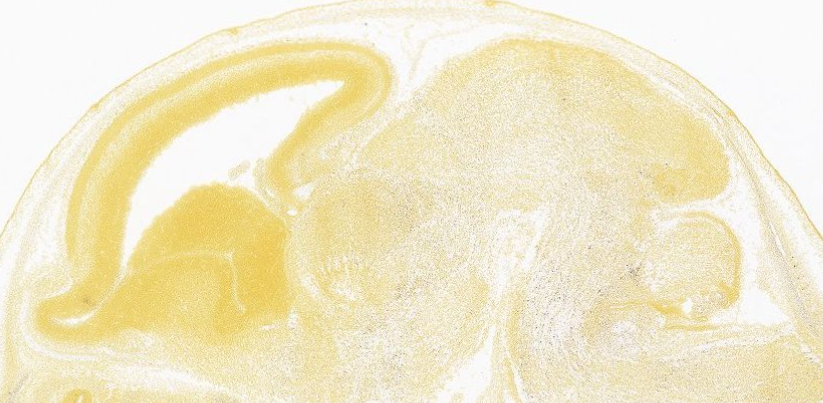 |
|  |  |  | E18.5 | Low  Expression | 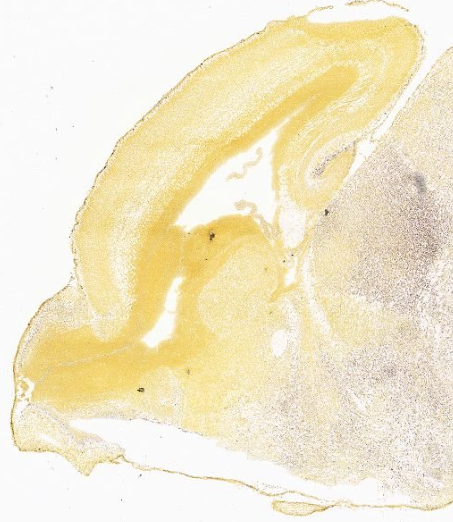 |
|  |  |  | P4 | High  Expression | 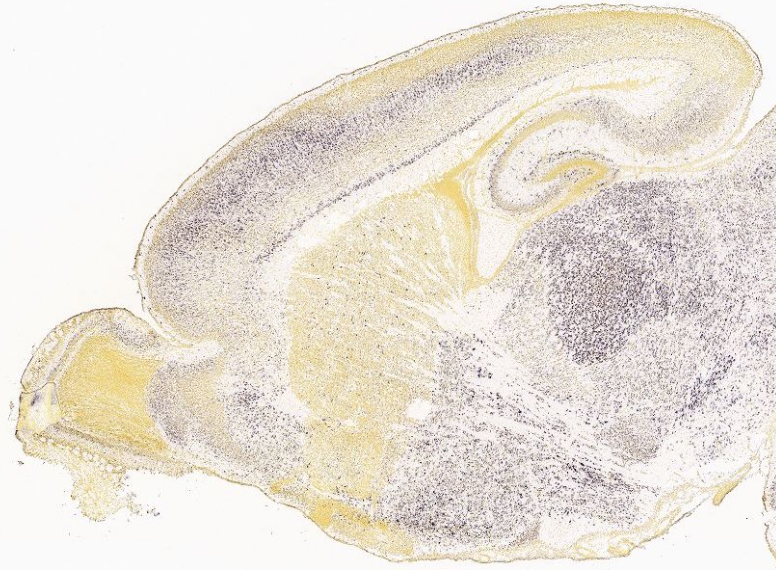 |
| MYT1L | HEAT MAP | | | [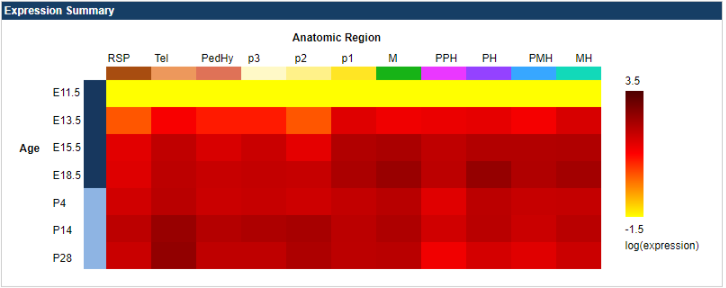](http://developingmouse.brain-map.org/gene/show/17700) | |
|  | IN SITUS | | E11.5 | Low  Expression | 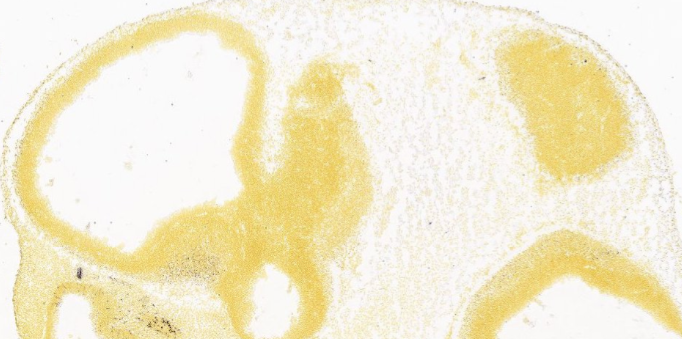 |
|  |  |  | E13.5 | High Expression | 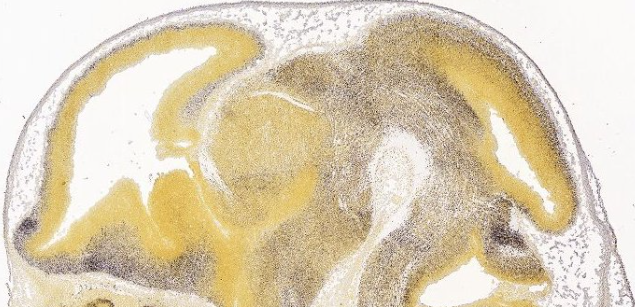 |
|  |  |  | E15.5 | High Expression | 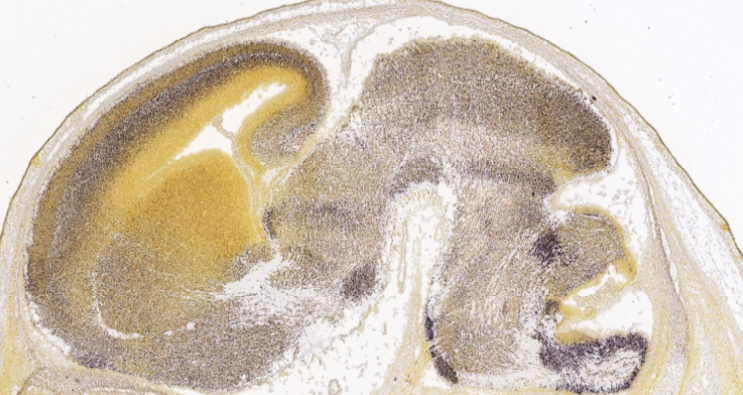 |
|  |  |  | E18.5 | High Expression | 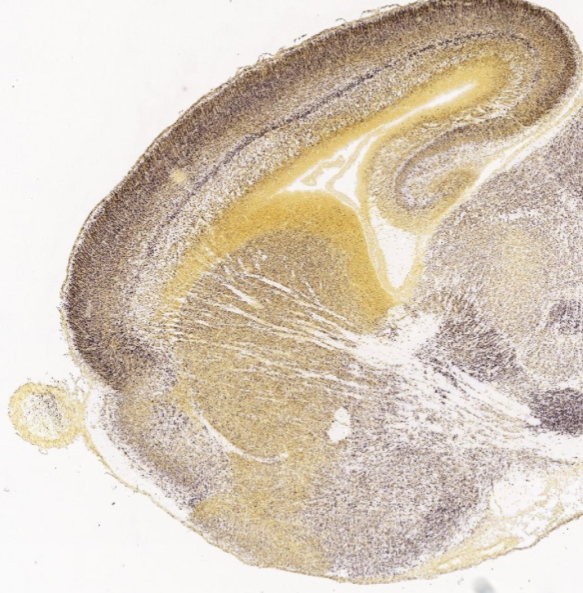 |
|  |  |  | P4 | High Expression | 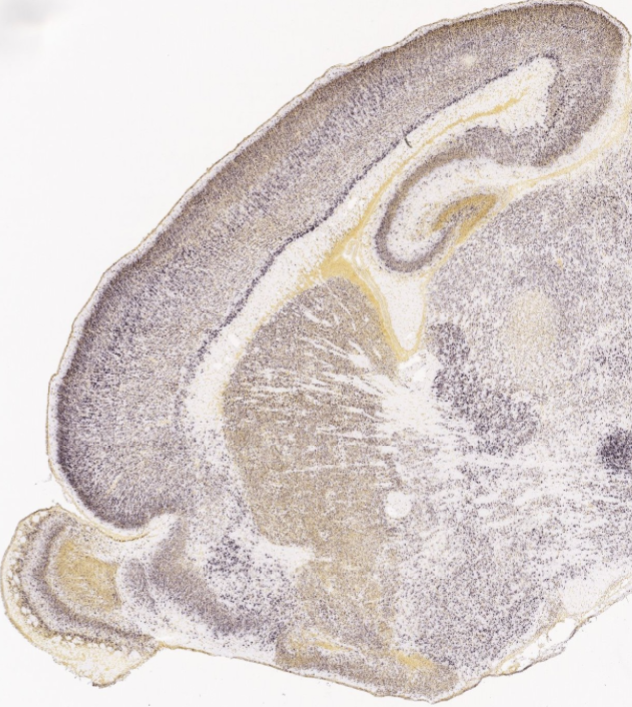 |
| PTEN | HEAT MAP | | | [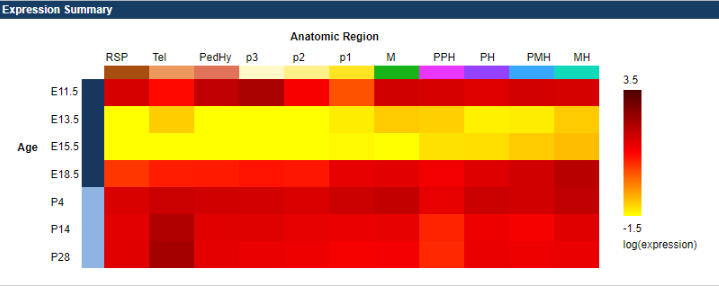](http://developingmouse.brain-map.org/gene/show/18974) | |
|  | IN SITUS | | E11.5 | High Expression | 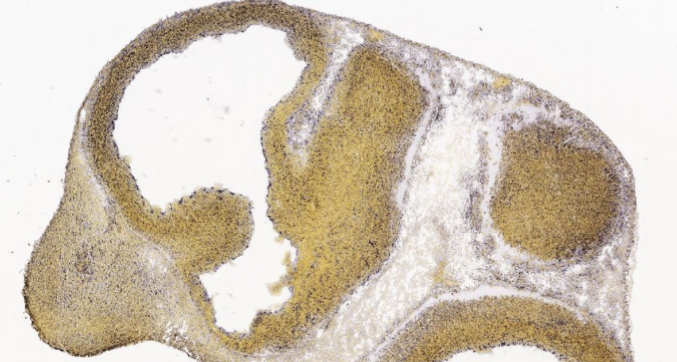 |
|  |  |  | E13.5 | No Expression | 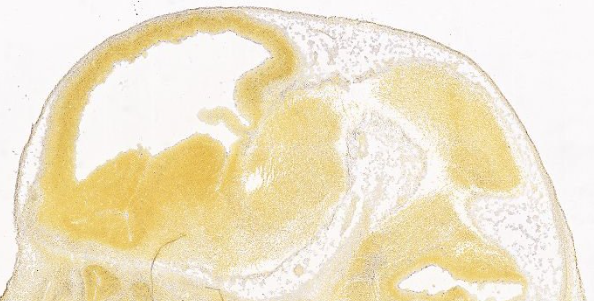 |
|  |  |  | E15.5 | No Expression | 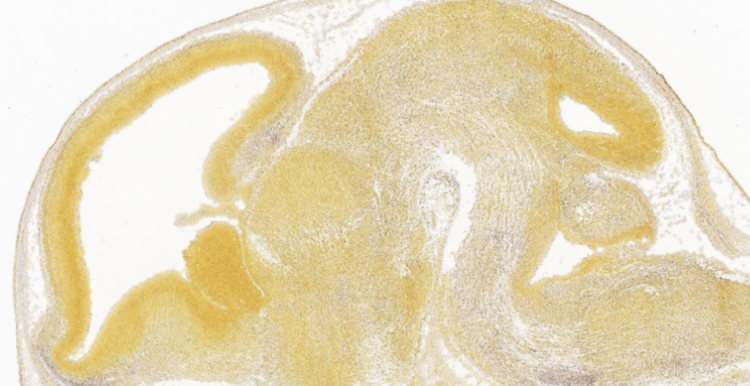 |
|  |  |  | E18.5 | Low  Expression | 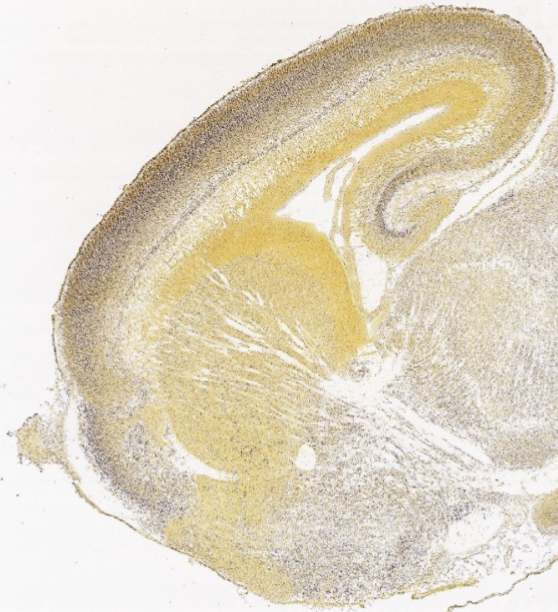 |
|  |  |  | P4 | High  Expression | 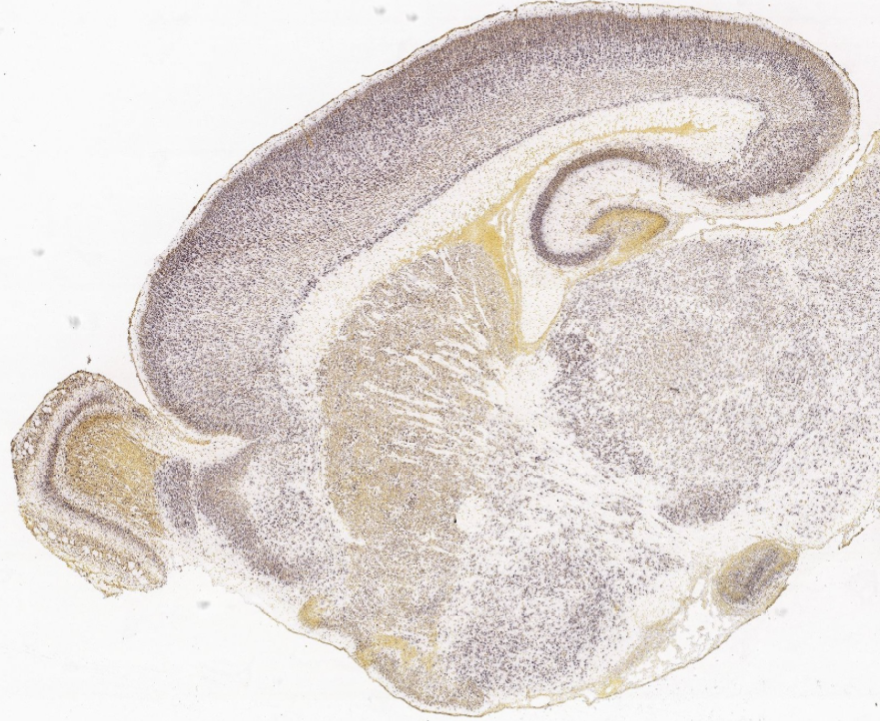 |
