# Supplementary material for: Identification of amygdala-expressed genes associated with autism spectrum disorder
Source: Mol Autism. 2020 May 27;11:39. doi: 10.1186/s13229-020-00346-1 (PMC7251751; doi:10.1186/s13229-020-00346-1)
Supplement: Supplementary file 6 — Additional file 6. Human genes in Allen Brain. [file 13229_2020_346_MOESM6_ESM.docx]

# Table of Contents

Page Content

1. ……………………………………………………………………………………….……..Table of Contents
2. ………………………………………………………………………………………………. Legends
3. ………………………………………………………………………………………………. PHF21A
4. ………………………………………………………………………………………………. KCNQ3
5. ………………………………………………………………………………………………. GPC5
6. ………………………………………………………………………………………………. RANBP17
7. ………………………………………………………………………………………………. [ELP4](#_ELP4)
8. ………………………………………………………………………………………………. [CNKSR2](#_CNKSR2)
9. ………………………………………………………………………………………………. [NAV2](#_NAV2)
10. ………………………………………………………………………………………………. [GDI2](#_GDI2)
11. ………………………………………………………………………………………………. [EIF2B3](#_EIF2B3)
12. ………………………………………………………………………………………………. [ATP6VE1](#_ATP6V1E1)
13. ………………………………………………………………………………………………. [PGAM1](#_PGAM_1)
14. ………………………………………………………………………………………………. [CCK](#_CCK)
15. ………………………………………………………………………………………………. [ATP6V1D](#_ATP6V1D)
16. ………………………………………………………………………………………………. [RSRC2](#_RSRC2)
17. ………………………………………………………………………………………………. [SAP18](#_SAP18)
18. ………………………………………………………………………………………………. [GFAP](#_GFAP)
19. ………………………………………………………………………………………………. [ARPP19](#_ARRP19)
20. ………………………………………………………………………………………………. [EIF1B](#_EIF1B)
21. ………………………………………………………………………………………………. [PLP1](#_PLP1)
22. ………………………………………………………………………………………………. [PCP4](#_PCP4)
23. ………………………………………………………………………………………………. [GRID2](#_GRID2)
24. ………………………………………………………………………………………………. [PCDH7](#_PCDH7)
25. ………………………………………………………………………………………………. [SLC2A13](#_SLC2A13)
26. ………………………………………………………………………………………………. [RASGEF1B](#_RASGEF1B)
27. ………………………………………………………………………………………………. [LRP1B](#_LRP1B)
28. ………………………………………………………………………………………………. [CCSER1](#_CCSER1)
29. ………………………………………………………………………………………………. [SLC26A3](#_SLC26A3)

Coronal Slices


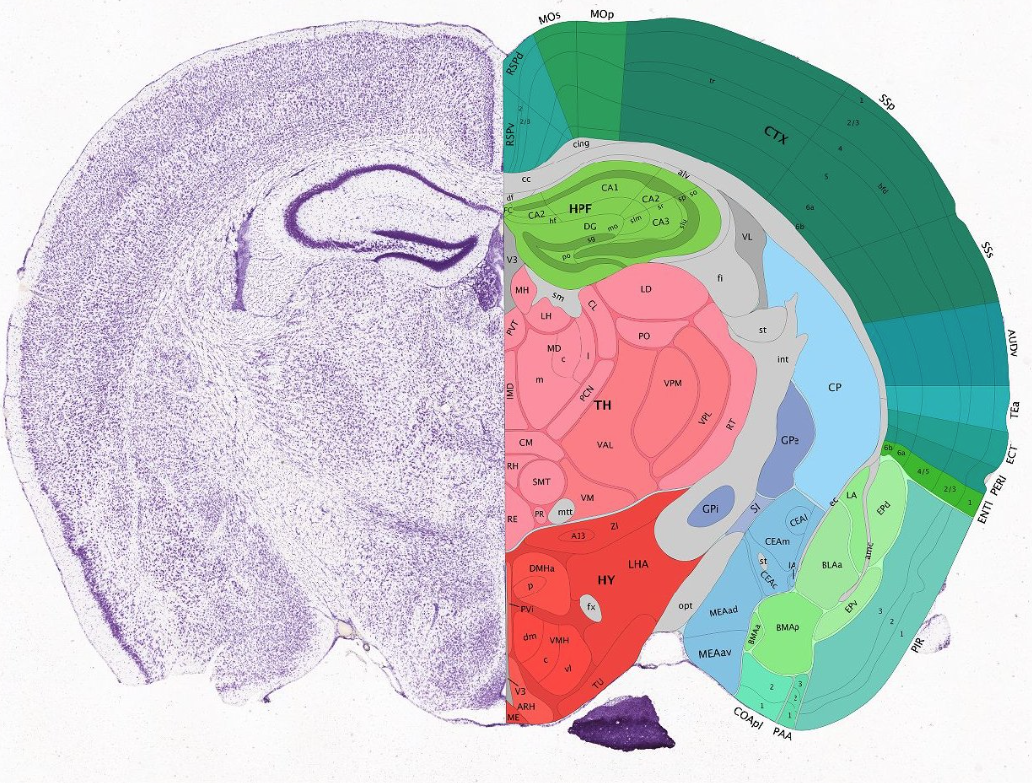


Sagittal Slices
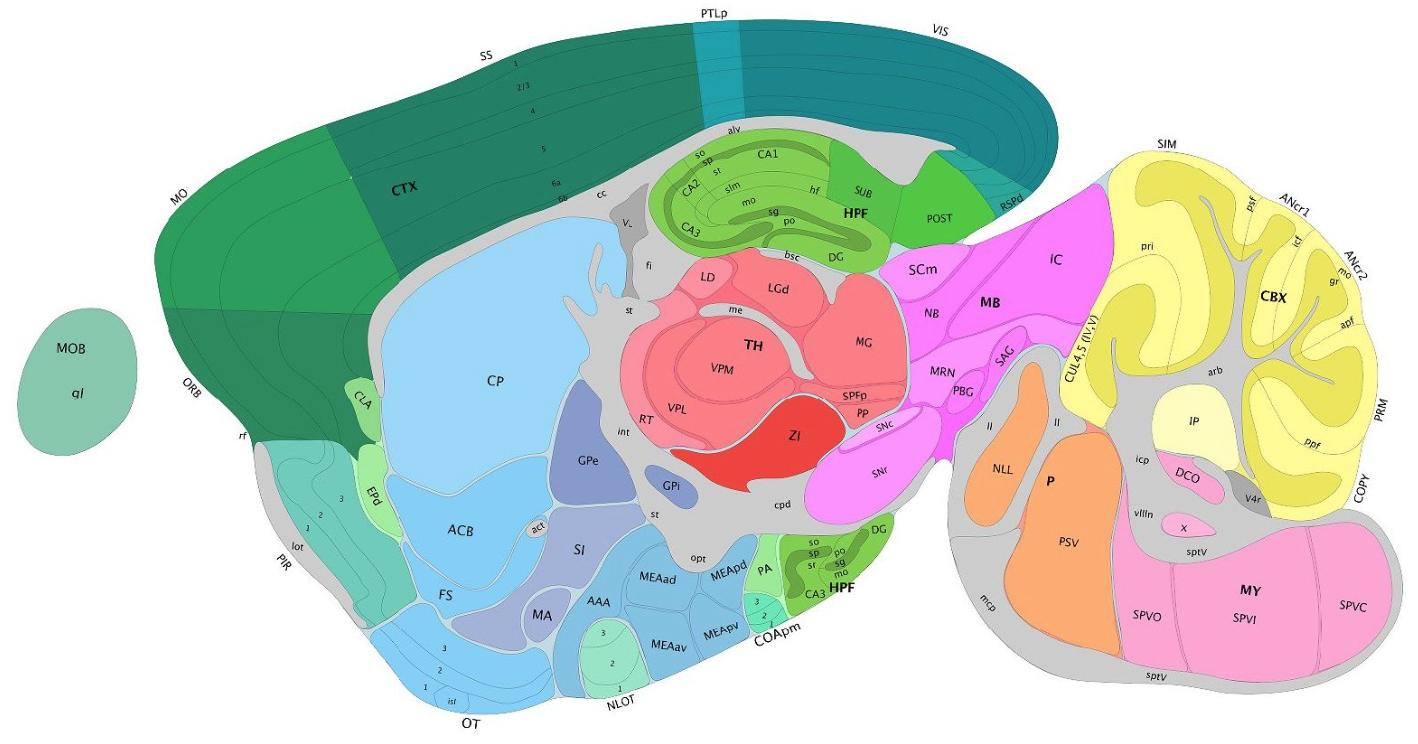


# PHF21A


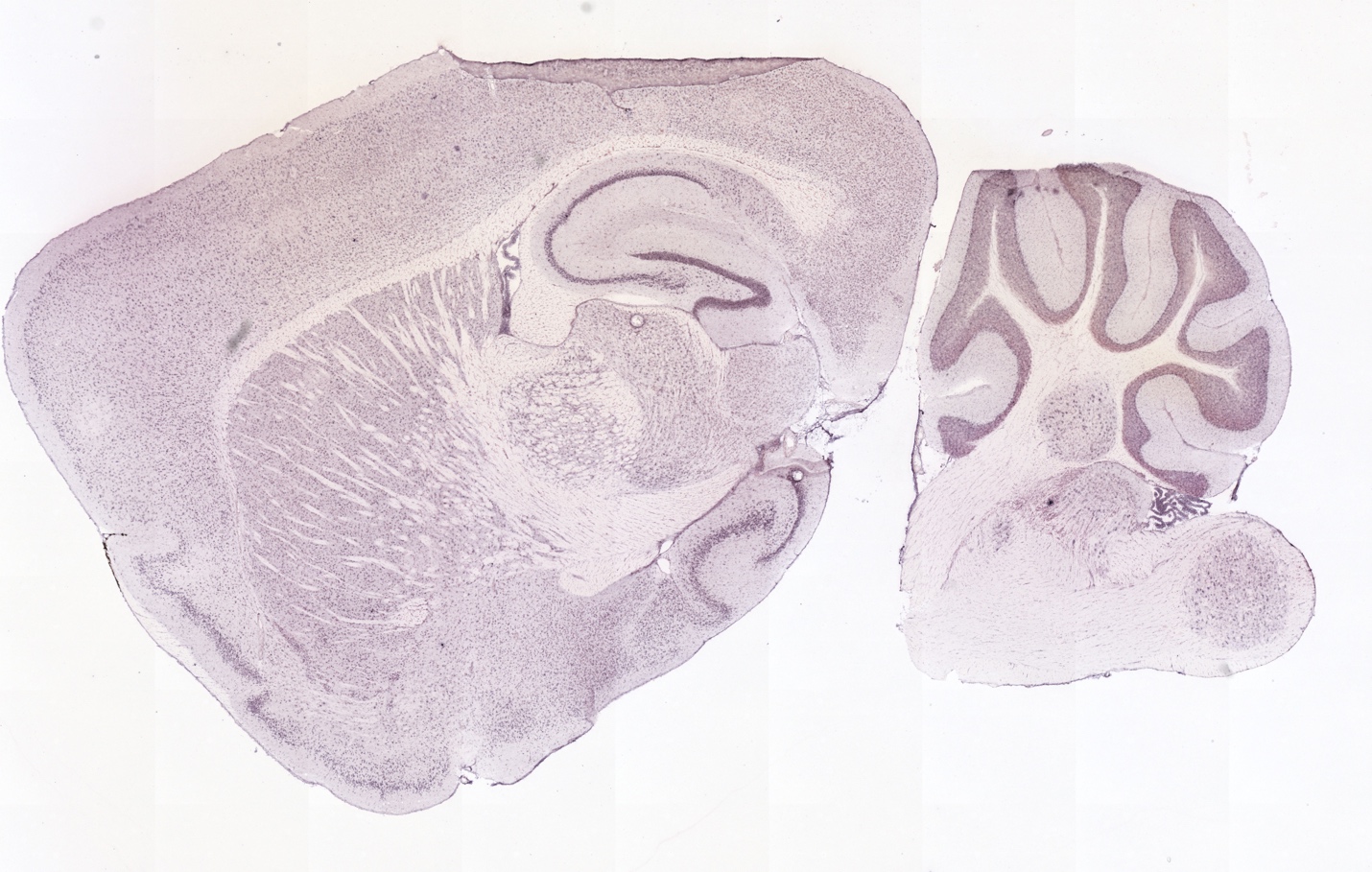


[Click Here to Return to Table of Contents](#_Table_of_Contents)

# KCNQ3


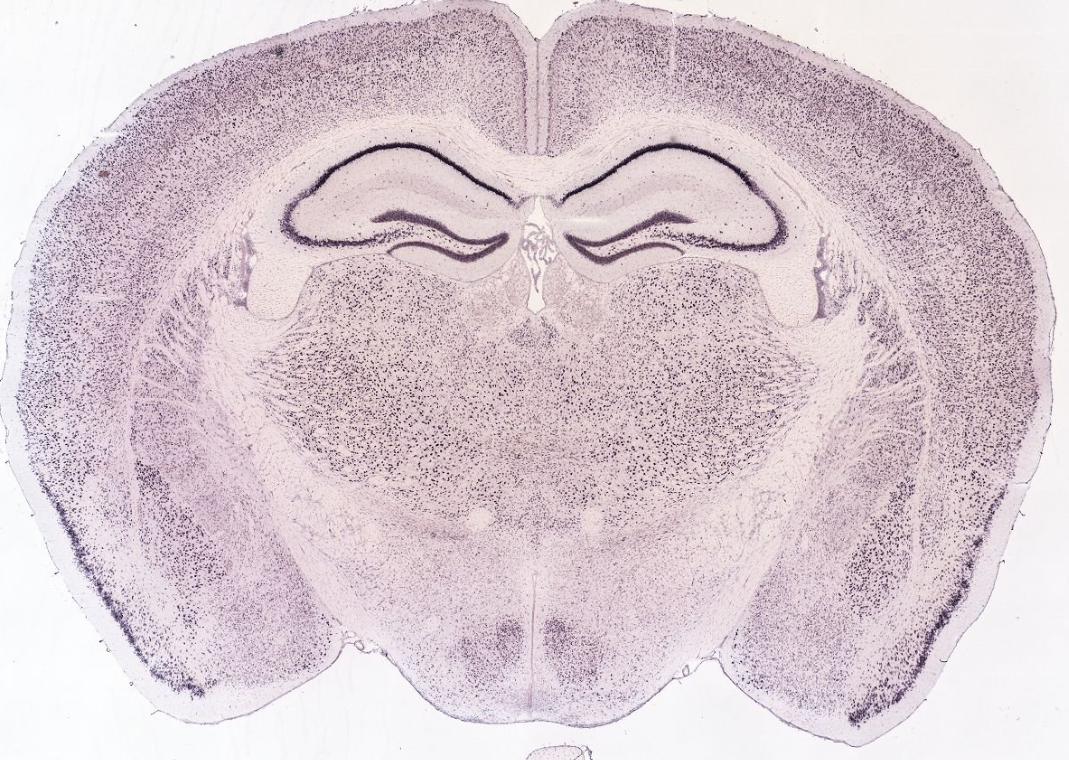


[Click Here to Return to Table of Contents](#_Table_of_Contents)

# GPC5


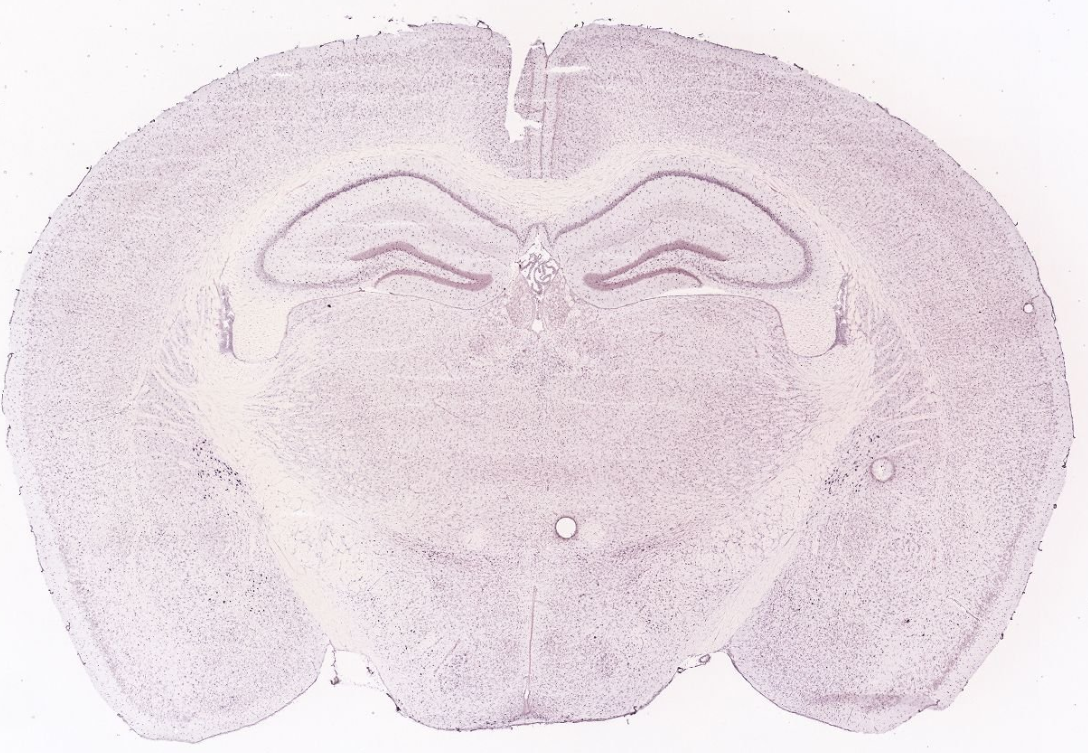


[Click Here to Return to Table of Contents](#_Table_of_Contents)

# RANBP17


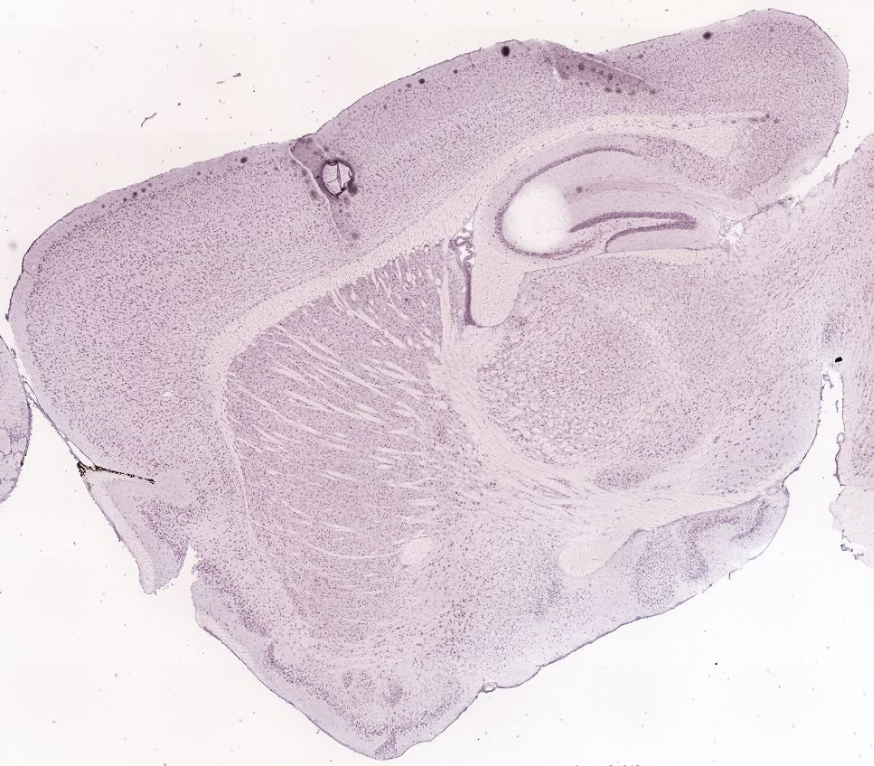


[Click Here to Return to Table of Contents](#_Table_of_Contents)

# ELP4


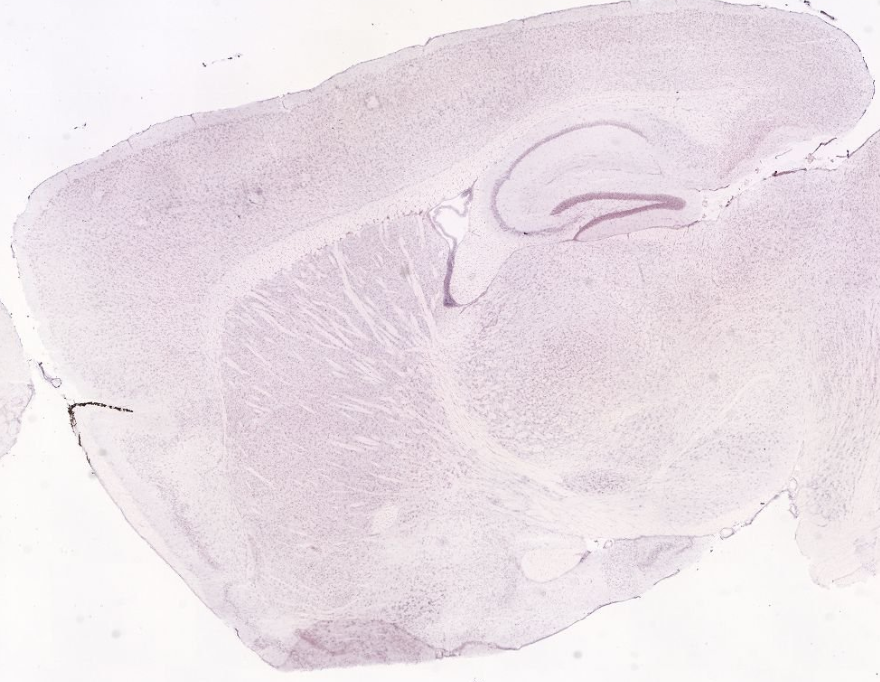


[Click Here to Return to Table of Contents](#_Table_of_Contents)

# CNKSR2


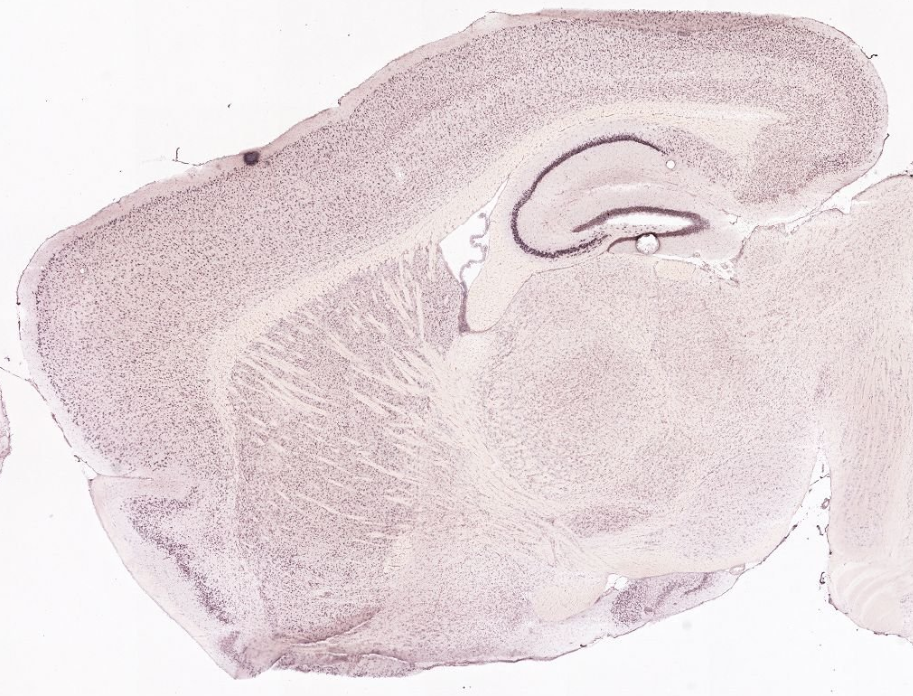


[Click Here to Return to Table of Contents](#_Table_of_Contents)

# NAV2


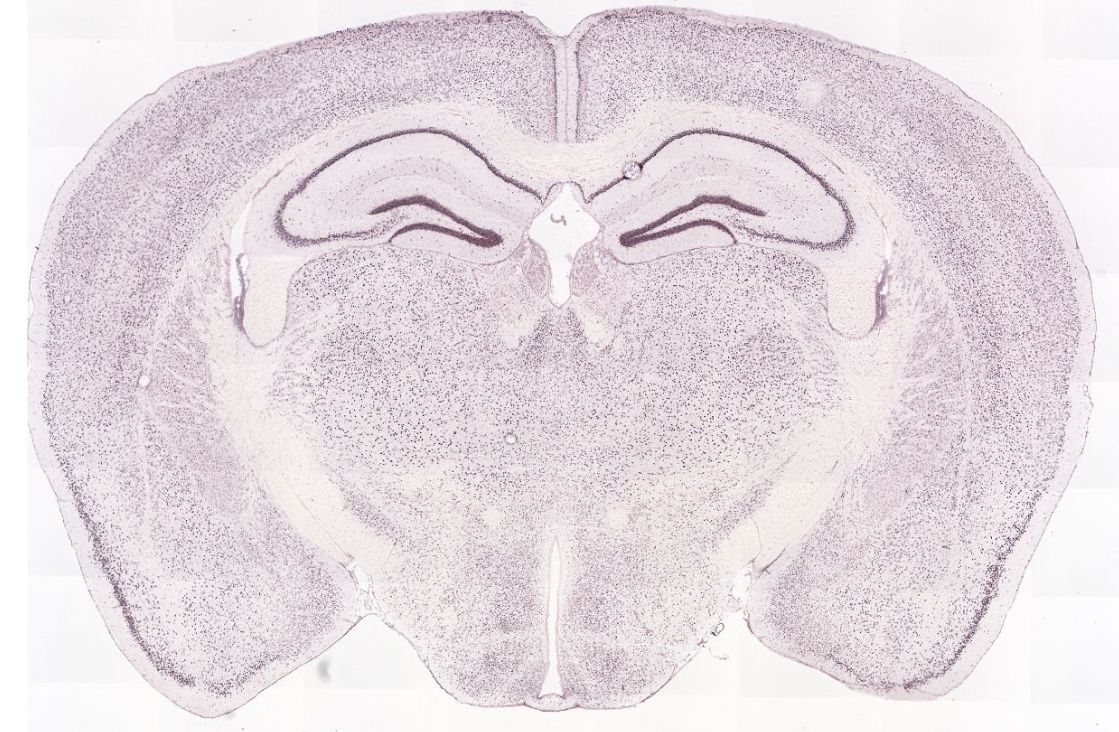


[Click Here to Return to Table of Contents](#_Table_of_Contents)

# GDI2


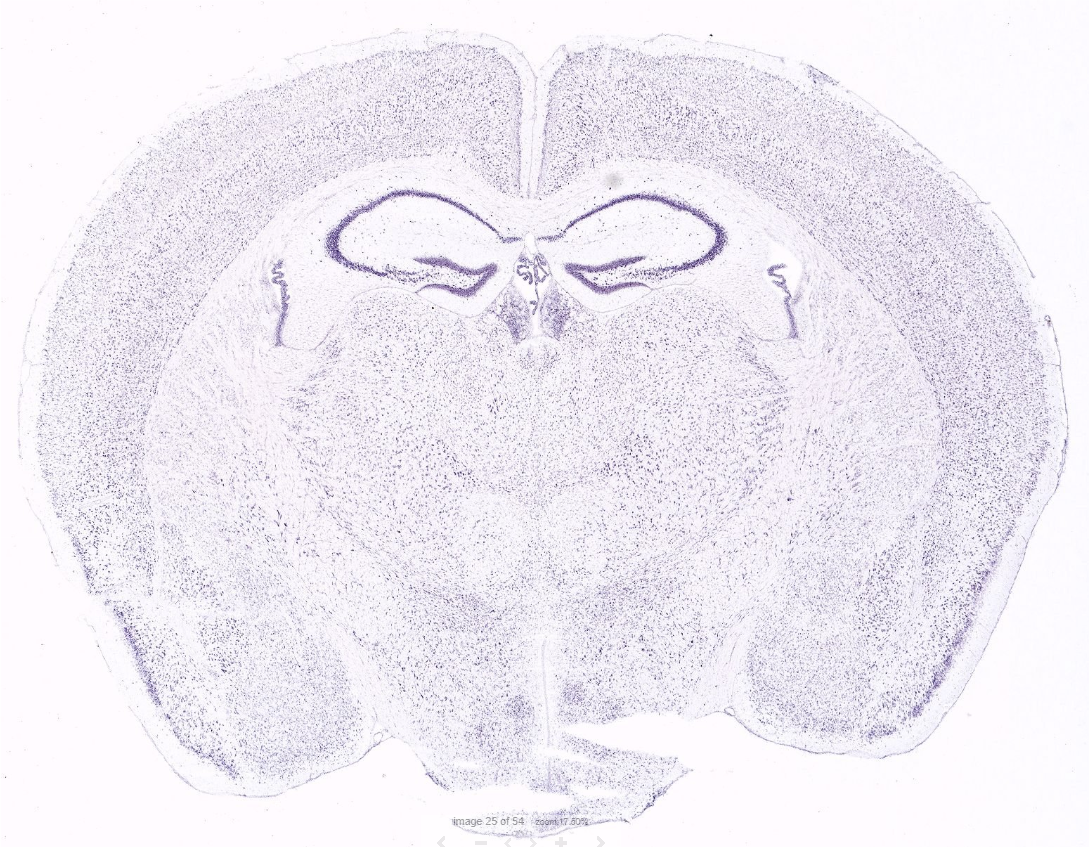


[Click Here to Return to Table of Contents](#_Table_of_Contents)

# EIF2B3


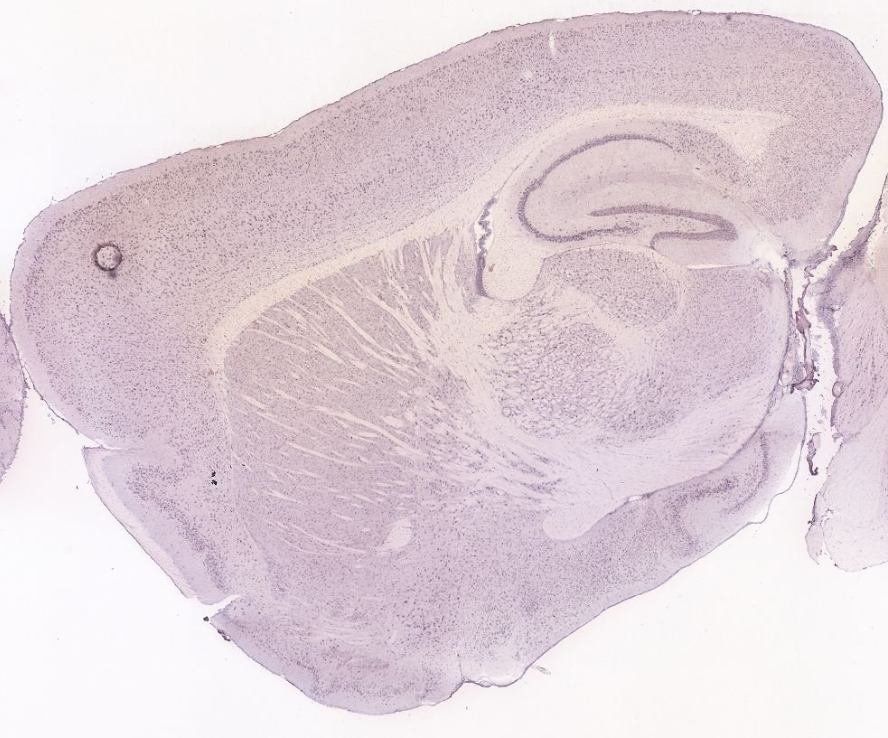


[Click Here to Return to Table of Contents](#_Table_of_Contents)

# ATP6V1E1


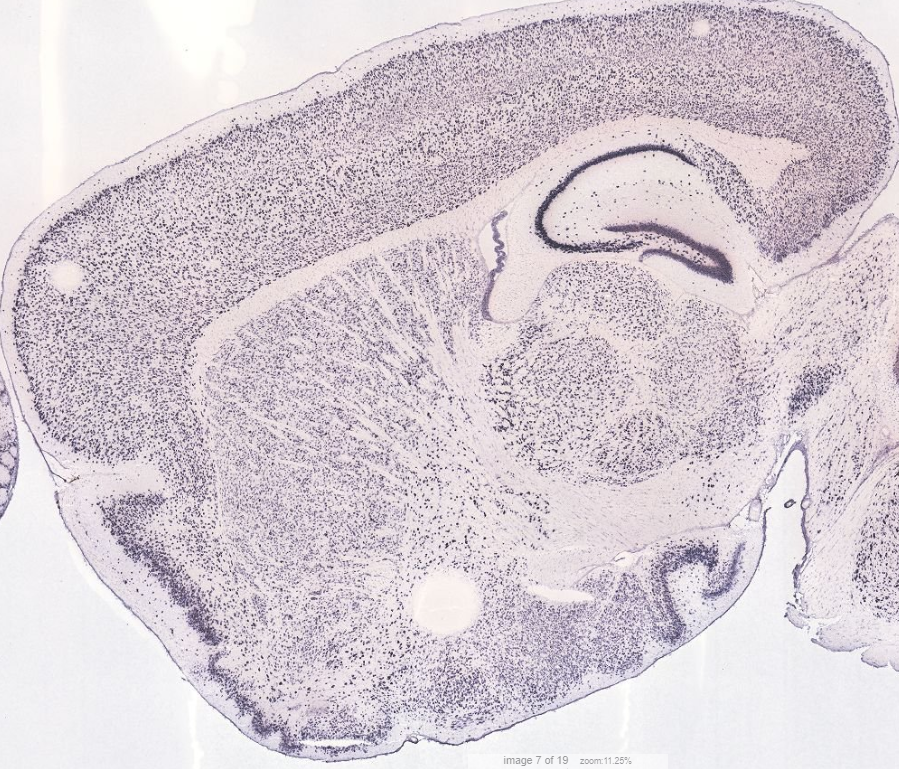


[Click Here to Return to Table of Contents](#_Table_of_Contents)

# PGAM 1


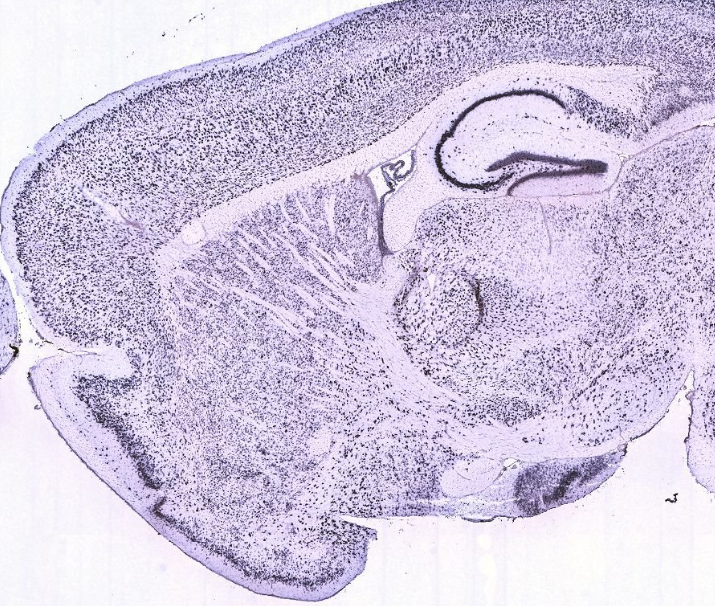


[Click Here to Return to Table of Contents](#_Table_of_Contents)

# CCK


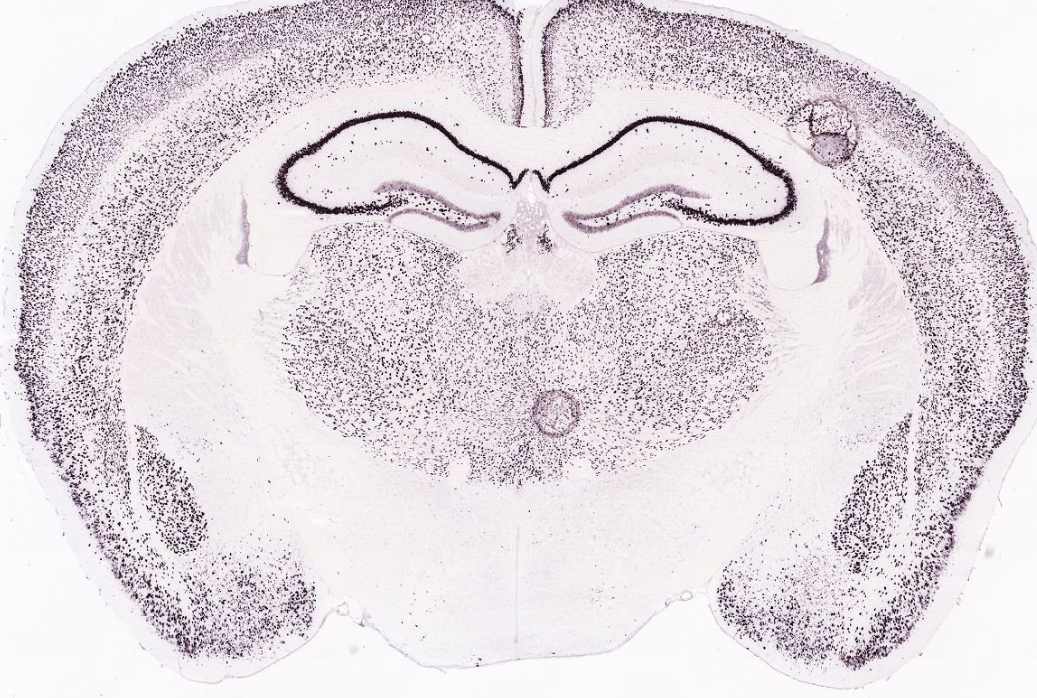


[Click Here to Return to Table of Contents](#_Table_of_Contents)

# ATP6V1D


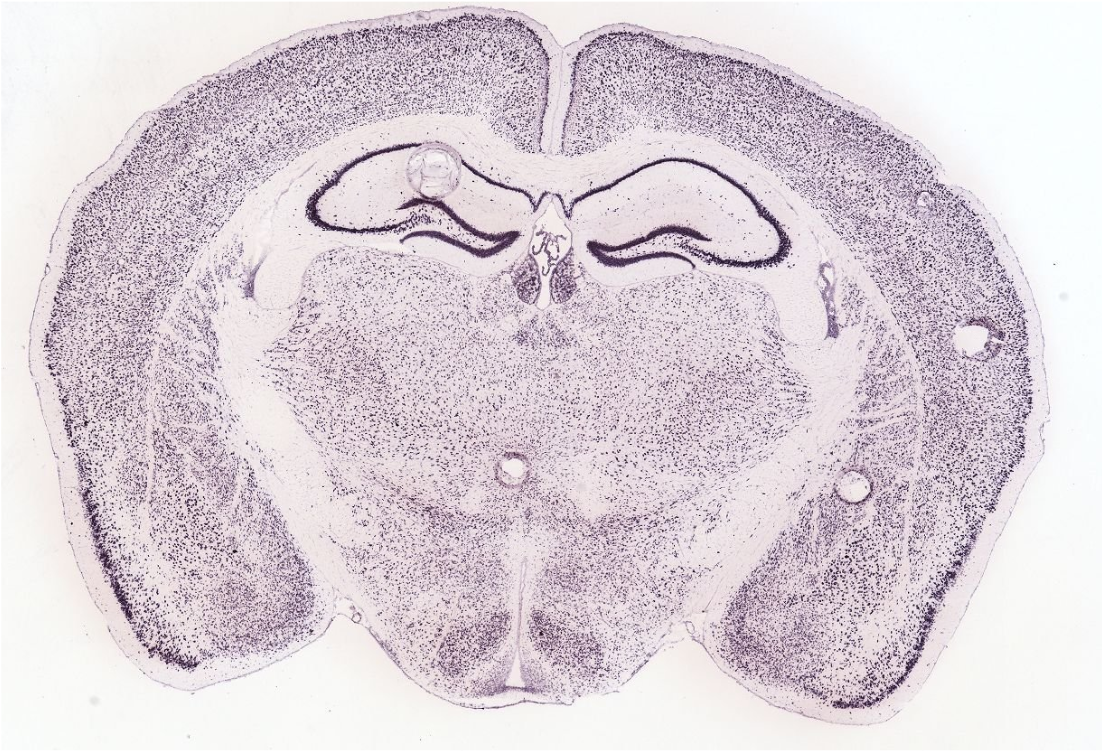


[Click Here to Return to Table of Contents](#_Table_of_Contents)

# RSRC2


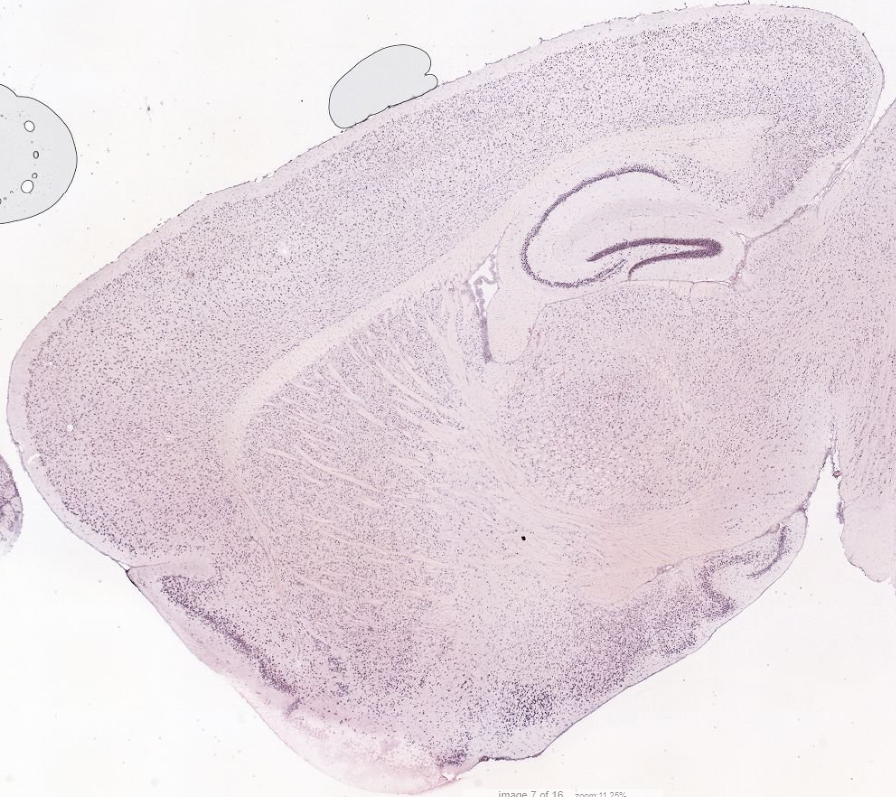


[Click Here to Return to Table of Contents](#_Table_of_Contents)

# SAP18


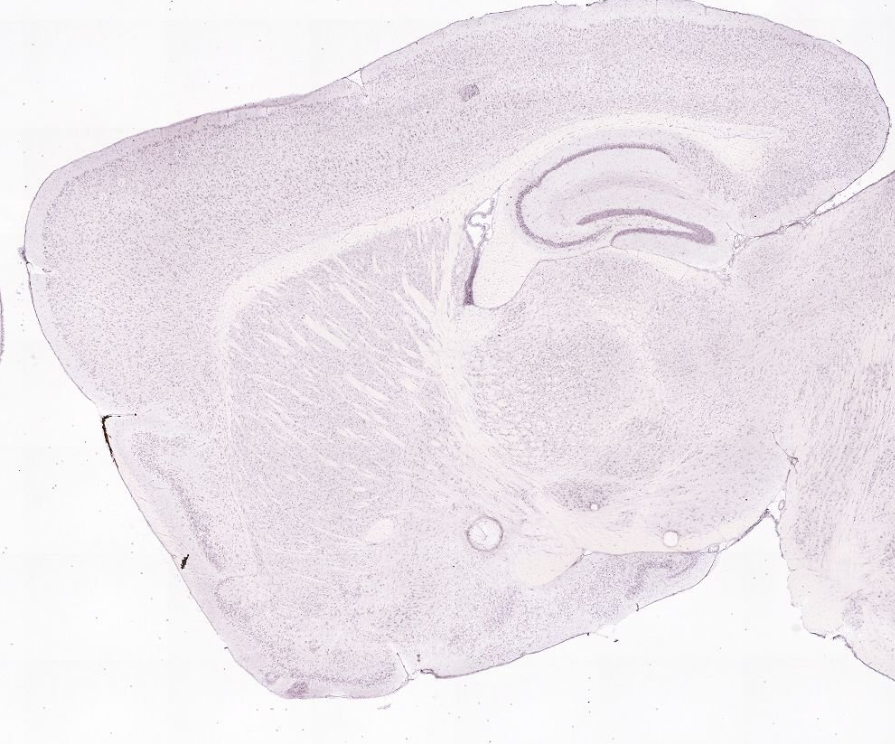


[Click Here to Return to Table of Contents](#_Table_of_Contents)

# GFAP


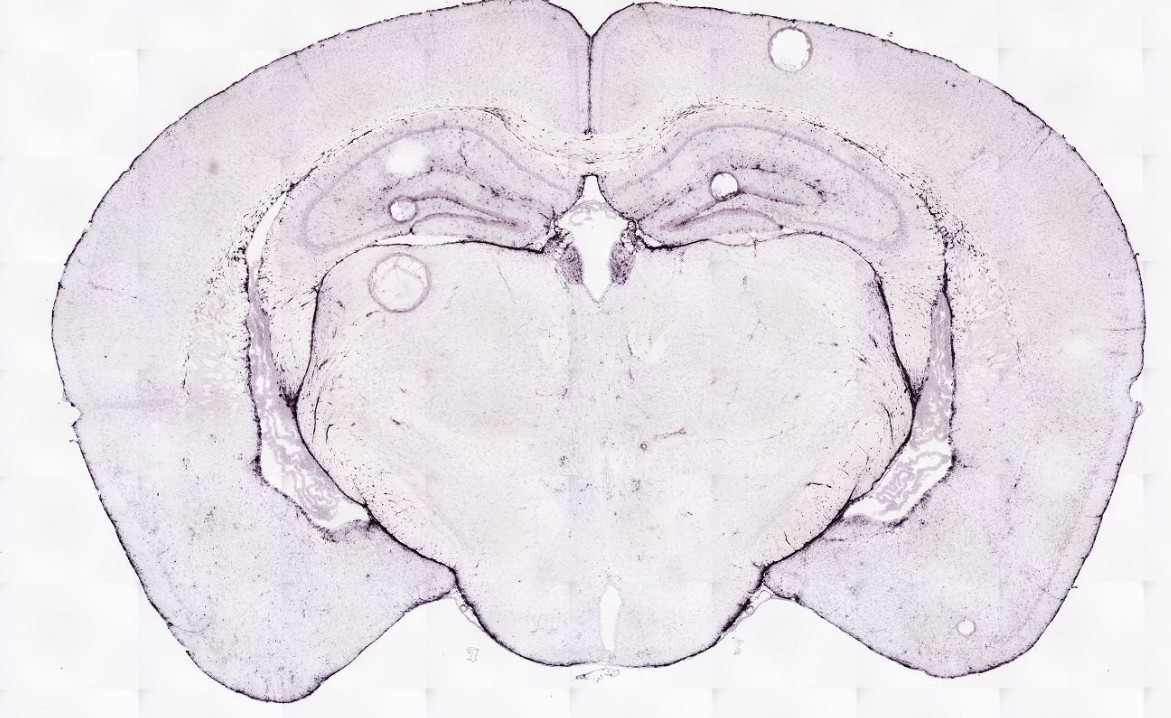


[Click Here to Return to Table of Contents](#_Table_of_Contents)

# ARRP19


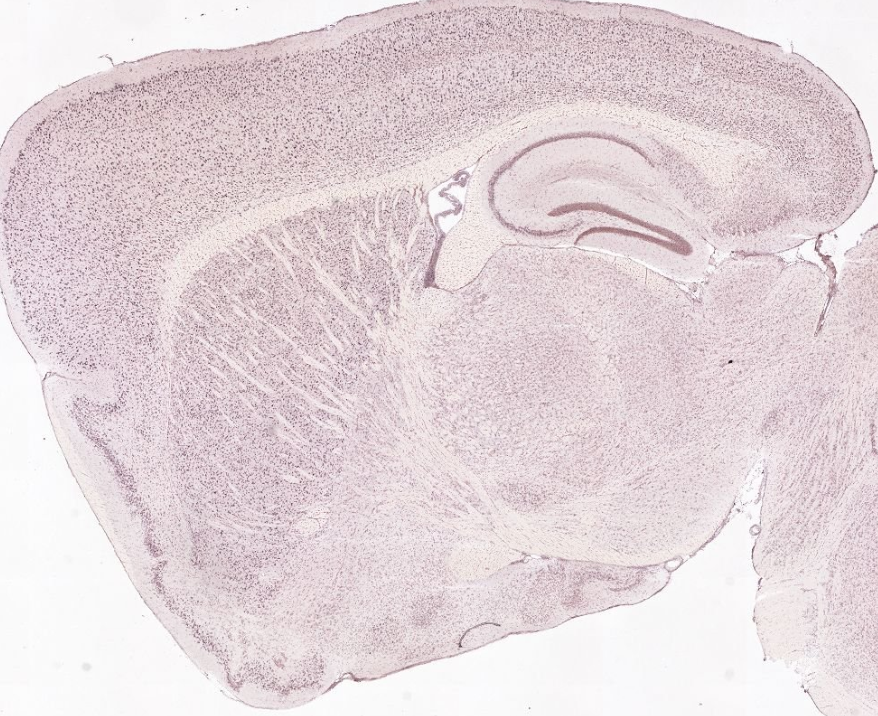


[Click Here to Return to Table of Contents](#_Table_of_Contents)

# EIF1B


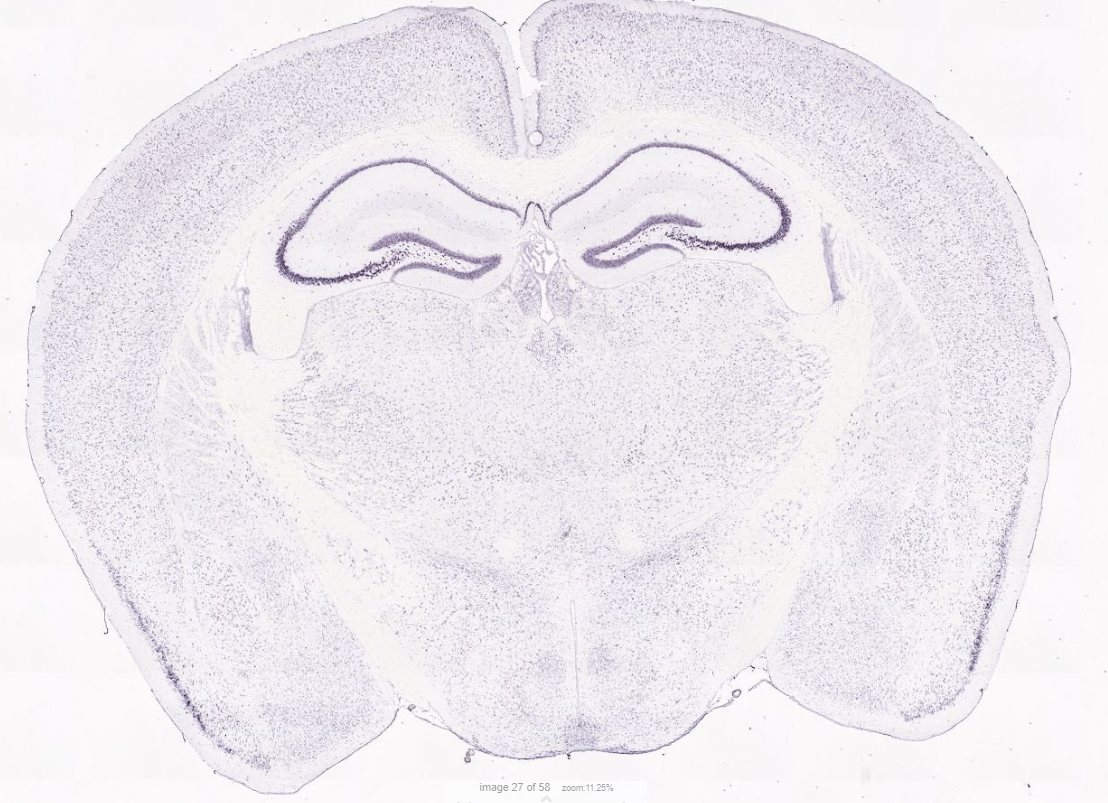


[Click Here to Return to Table of Contents](#_Table_of_Contents)

# PLP1


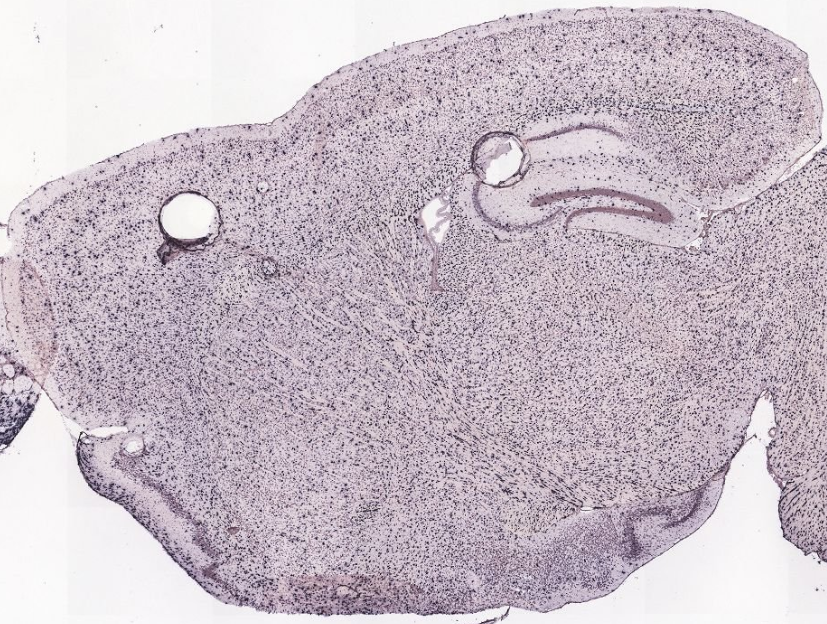


[Click Here to Return to Table of Contents](#_Table_of_Contents)

# PCP4


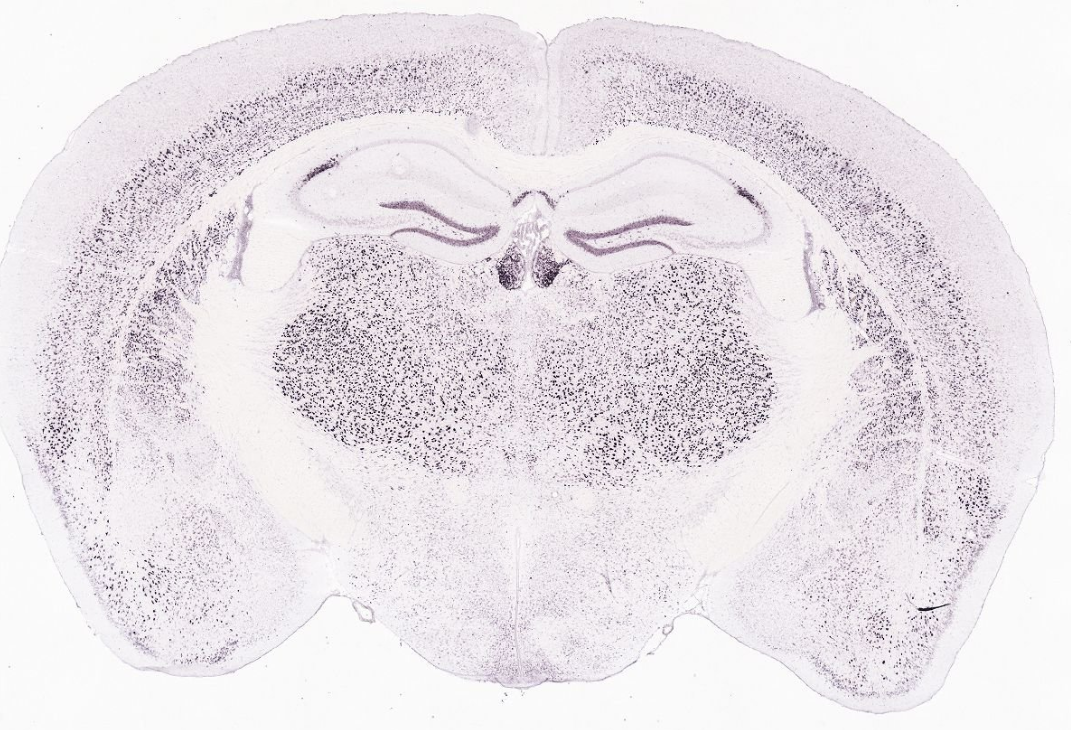


[Click Here to Return to Table of Contents](#_Table_of_Contents)

# GRID2


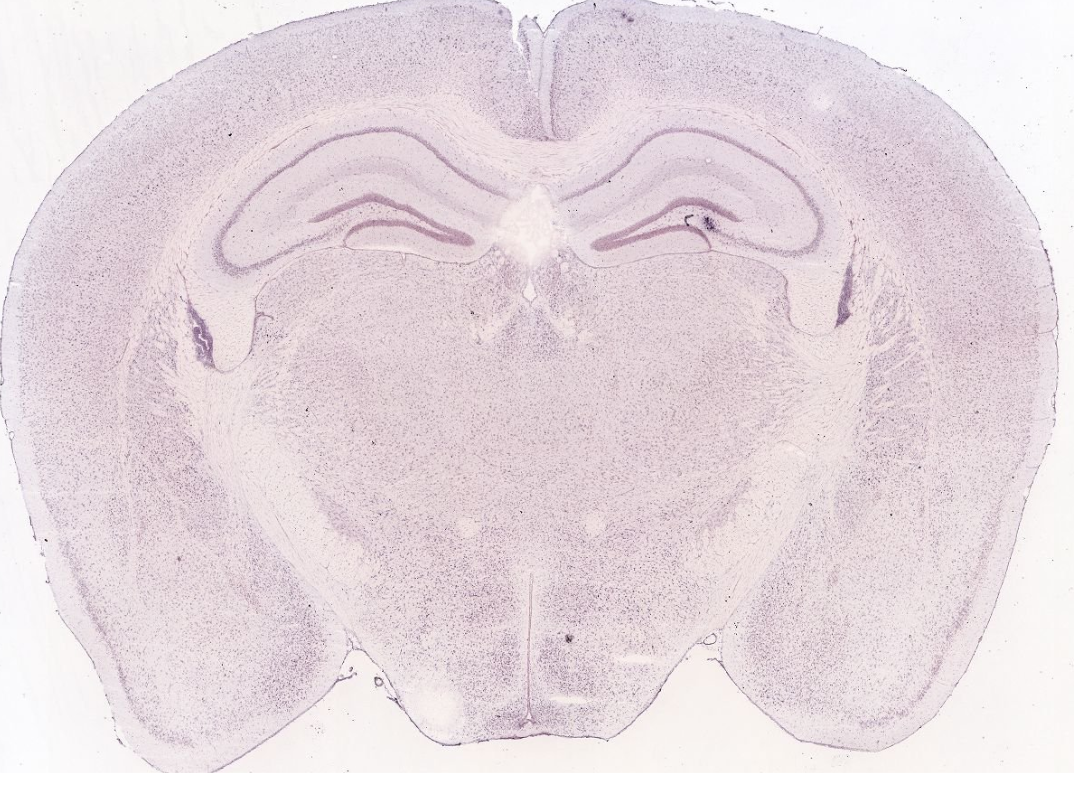


[Click Here to Return to Table of Contents](#_Table_of_Contents)

# PCDH7


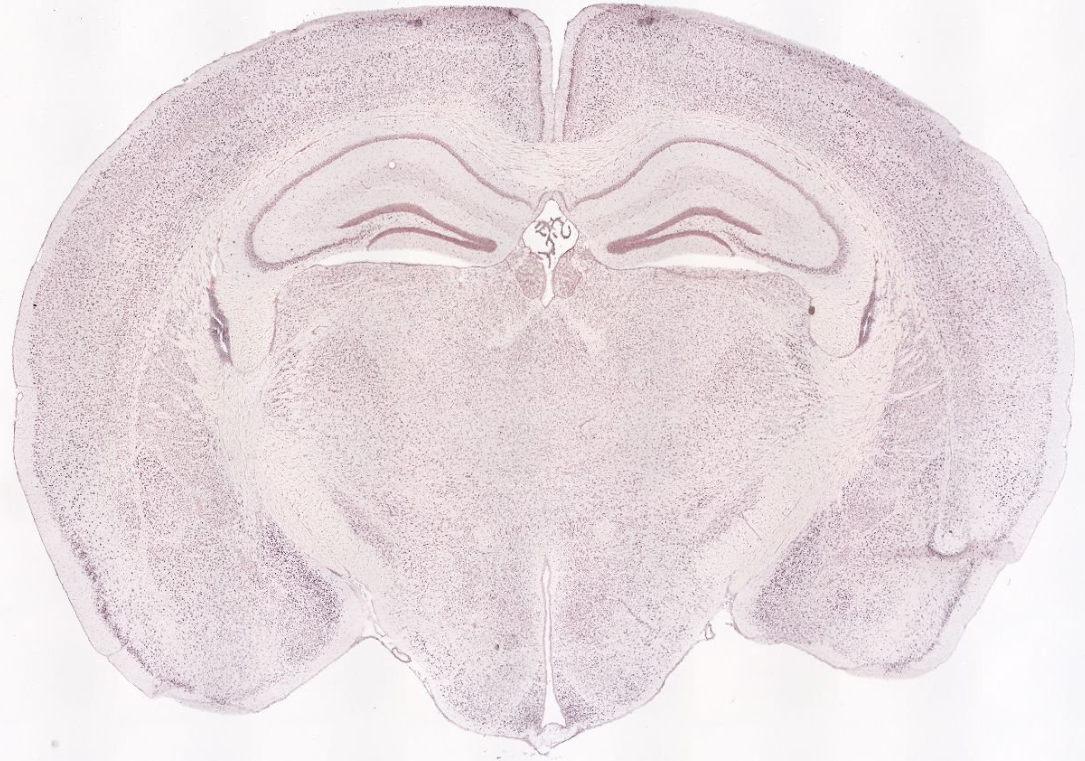


[Click Here to Return to Table of Contents](#_Table_of_Contents)

# SLC2A13


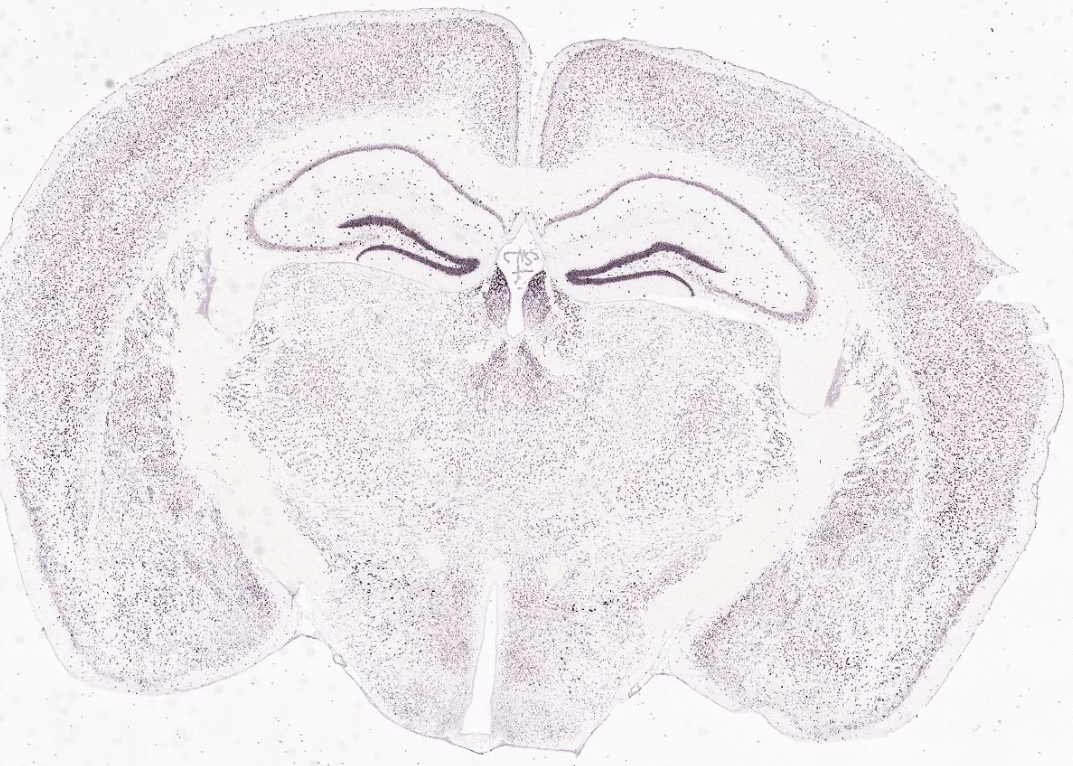


[Click Here to Return to Table of Contents](#_Table_of_Contents)

# RASGEF1B


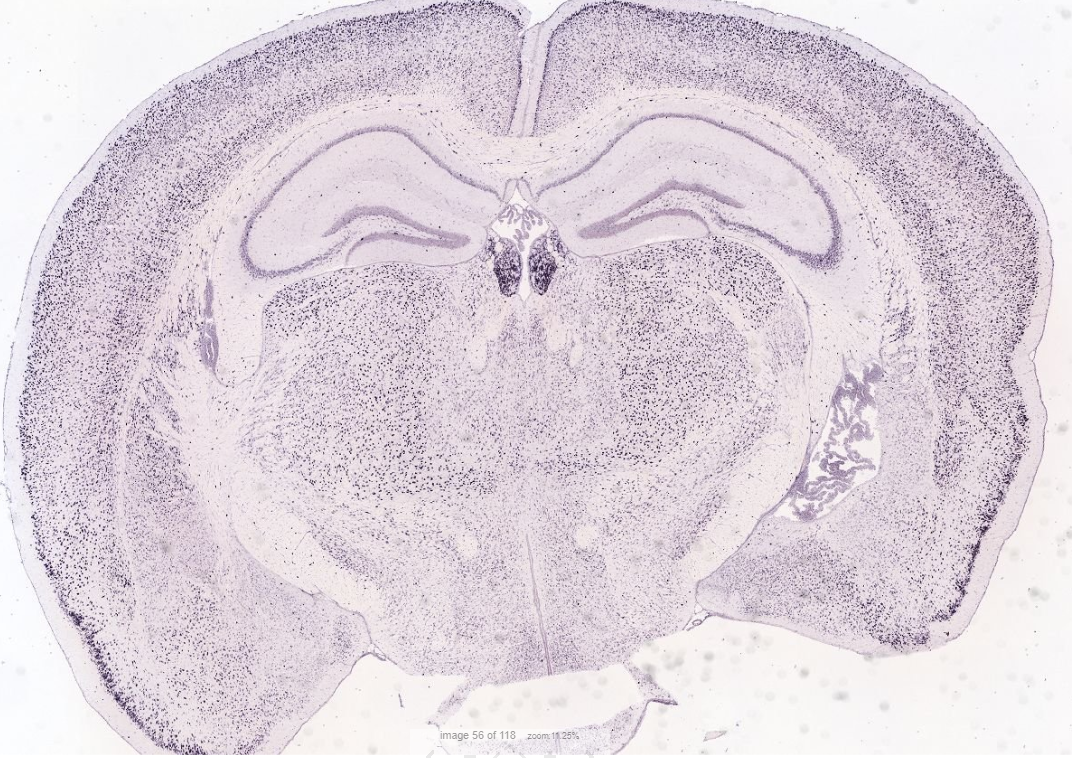


[Click Here to Return to Table of Contents](#_Table_of_Contents)

# LRP1B


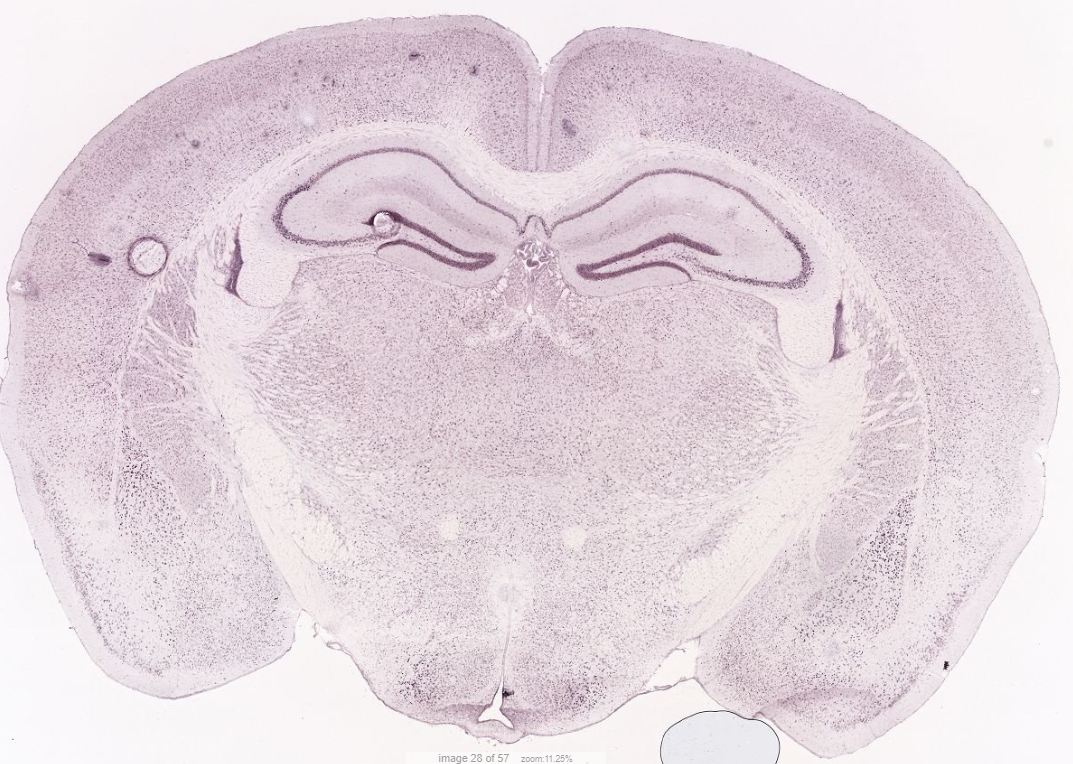


[Click Here to Return to Table of Contents](#_Table_of_Contents)

# CCSER1


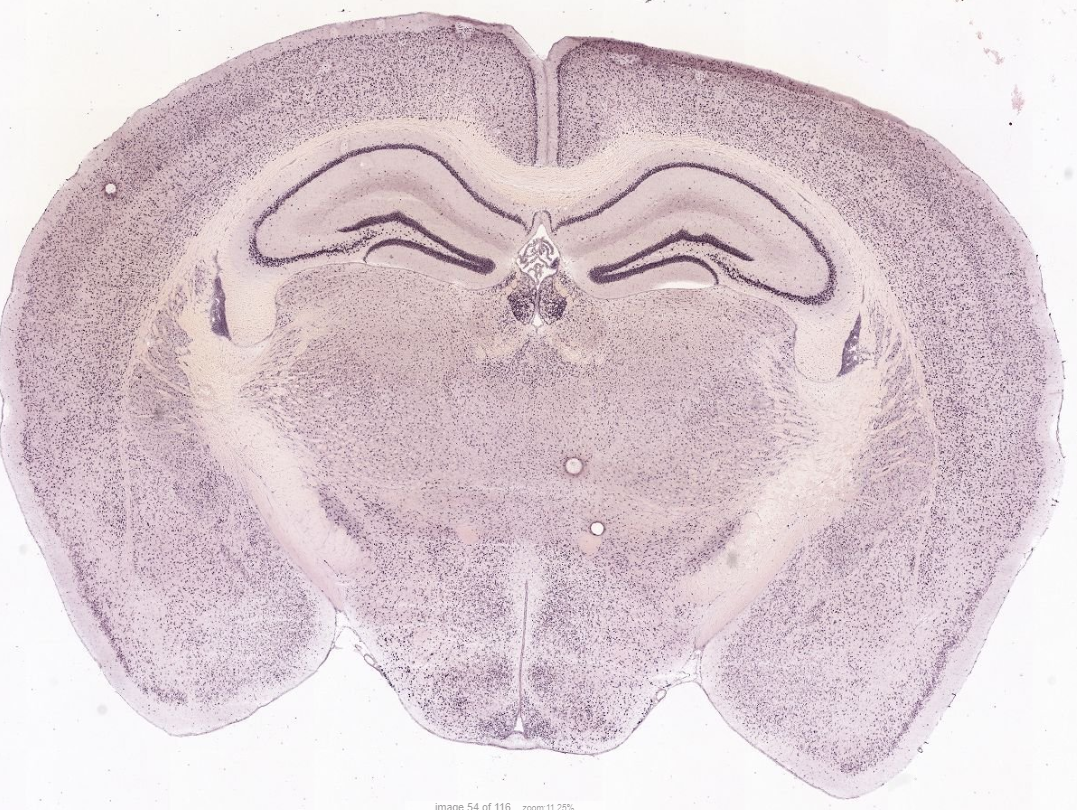


[Click Here to Return to Table of Contents](#_Table_of_Contents)

# SLC26A3


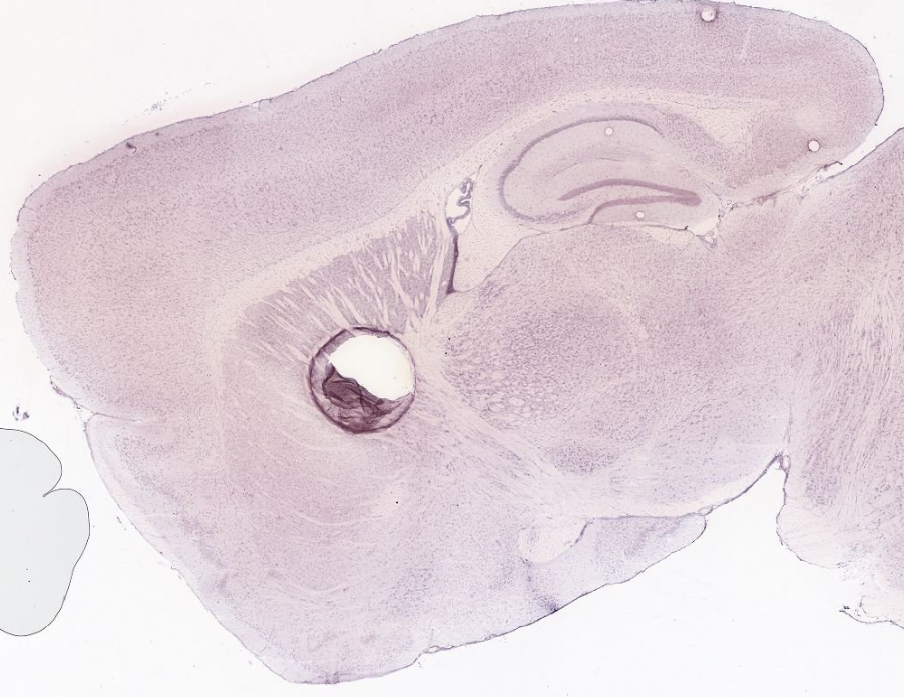


[Click Here to Return to Table of Contents](#_Table_of_Contents)
